# Computational reconstruction of evolutionary selection in human brain networks

**DOI:** 10.3389/fninf.2025.1623174

**Published:** 2026-01-26

**Authors:** Lukasz Piszczek, Clara Fazzari, Sophia Ulonska, Katja Bühler, Wulf Haubensak

**Affiliations:** 1 Department of Neuronal Cell Biology, Center for Brain Research, Medical University of Vienna, Vienna, Austria; 2 Department of Biology, University of Rome Tor Vergata, Rome, Italy; 3 Biomedical Image Informatics, VRVis GmbH, Vienna, Austria; 4 Research Institute of Molecular Pathology (IMP), Vienna Biocenter (VBC), Vienna, Austria

**Keywords:** adaptive evolution, archaic humans, brain evolution, computational neuroarcheology, evolutionary genetics, social brain evolution

## Abstract

**Introduction:**

The accumulation of genomic and brain data opens new opportunities for resource friendly, data driven brain exploration. A key challenge is to develop versatile and accessible strategies that integrate and mine multimodal datasets for novel neuroscientific insights. Here, we optimized an integrated workflow for mapping multigenic evolutionary traits in the human brain across cognitive, cellular, and molecular levels.

**Methods:**

At the input stage, the workflow fuses an evolutionary genetic dataset with searchable synthetic functional magnetic resonance imaging (fMRI) databases that are pre clustered into concise psychological domains for improved interpretability. At its core, a Genetic Algorithm for Generalized Biclustering (GABi) mines gene sets under evolutionary selection that also show high expression correlation with fMRI networks.

**Results:**

Applying this workflow, we identified evolutionary patterns spanning cognitive traits, brain cell types, and molecular mechanisms. Focusing on socio affective traits, the algorithm highlighted peaks in adaptive selection in networks for social interaction (language) and social concepts (theory of mind) across hominid, early hominin, and anatomically modern human (AMH) ancestry. These traits emerge from a broad spectrum of excitatory (glutamatergic) and inhibitory (GABAergic) neuronal, as well as non neuronal, cell types. The associated Gene Ontology (GO) terms were enriched for cell signaling, synaptic organization, and neuronal morphology.

**Discussion:**

Together, these findings demonstrate an integrated workflow for molecular to systems level exploration of the brain and provide new perspectives on the evolutionary history of human socio affective functions. This approach can be adapted to screen for functional traits in the context of mental disorders or applied to the brains of other phylogenies in a similar manner.

## 1 Introduction

### 1.1 Multimodal brain data: a new frontier for neuroscientific discovery

An increasing amount of brain data, along with neurogenetic information on brain development, cell types, and diseases, offers unprecedented opportunities for neuroscientific exploration and hypothesis generation. Moreover, these datasets provide footholds with problems that are costly or difficult to address through conventional experimental approaches.

At the neurogenetic level, multiple large-scale neurogenetic databases contain gene expression and genomic information across brain regions, developmental stages, and species, which are essential resources for understanding brain function. The Allen Brain Atlas offers genome-wide 3D maps of gene expression across mouse, human, and non-human primate brains, whereas the BrainSpan Atlas provides multidimensional genomic data across developing and adult human brain regions. Projects such as the Genotype-Tissue Expression (GTEx) project catalog genetic effects on gene expression across 49 human tissues, including multiple brain regions, providing cis- and trans-expression quantitative trait loci (eQTLs) and splicing QTLs that link genetic variation to functional outcomes. Initiatives such as BrainBase and databases such as OpenTargets and DisGeNE curate disease-gene associations and disorder-specific profiles.

At the system level, neuroimaging repositories span from primary data sources, such as the Human Connectome Project (HCP), which provides task-based and resting-state functional magnetic resonance imaging (fMRI) data from large cohorts, to synthetic databases, such as Neurosynth and NeuroQuery. These databases aggregate published neuroimaging findings to create probabilistic brain maps linked to cognitive functions.

With the growing neurogenetic and imaging datasets, the scientific community and funding systems have invested significantly in providing access to such multimodal resources. Both privately (e.g., Allen Brain Institute) and publicly (e.g., BRAIN Initiative, Human Brain Project) funded multimodal brain mapping initiatives provide regional and local (cellular) gene expression data, connectomics, and neuronal activity data, integrated by emerging spatial omics approaches (Bressan et al., 2023). The next frontier is the establishment of low-threshold, resource-friendly workflows with variable statistical search thresholds and approaches that mine these resources for insights into brain function (i.e., encompassing cognition and behavior in the context of this study) (Pfaff et al., 2019).

### 1.2 Integrating genetics with brain function

Among such approaches, pipelines linking genetics to brain function are gaining popularity, as they can be used to annotate and deconstruct the functional organization of the brain (Ganglberger et al., 2018; Richiardi et al., 2015; Dear et al., 2024; Fornito et al., 2019). One such prime example is a study linking genome-wide association studies (GWAS) to Diffusion Weighted Imaging (DWI) data, allowing for filtering significant single-nucleotide polymorphism (SNP)-connectome pairs to systematically identify associations between genetic variants and structural connectivity phenotypes. Finally, such associations can be then linked to cognitive traits (Chen et al., 2022). Moreover, advances in single-cell sequencing allow for the mapping of brain region-specific features down to individual cell types and the identification of gene markers that define circuit architecture, supporting trait mapping at the cellular resolution (Yao et al., 2025; Lee et al., 2025). These strategies collectively help pinpoint the brain regions and genetic mechanisms that underlie the trait-relevant network architecture (Arkhipov et al., 2025). Likewise, a cross-species resting-state fMRI imaging study using several mouse models of autism has successfully identified four etiologically relevant functional connectivity subtypes for this disorder (Zerbi et al., 2021). This demonstrates the power of computational strategies to integrate resting-state fMRI connectivity data across various mouse genetic models to identify specific brain networks underlying functional traits and psychiatric disorders.

Importantly, an increasing number of tools are available, allowing the mining of such complex data. These tools can be grouped into three approaches. Neuromaps (Markello et al., 2022) and abagene (Markello et al., 2021) integrate, transform and compare molecular, structural and functional maps of the brain by combining classical statistics (*t*-test, correlation, permutation test) and machine learning methods (regression, clustering). Neurosynth, Neuroquery and NiMARE (Salo et al., 2023) apply literature and data meta-analysis approaches to reconstruct brain-wide networks underlying specific functions. Finally, tools such as CellWhisperer (Schaefer et al., 2025) or CellTransformer (Lee et al., 2025), which are artificial intelligence (AI)-based workflows, currently allow the integration of single-cell expression brain data and deep data mining.

### 1.3 Evolutionary approaches: genetics, archeology, and comparative data

The longstanding quest to reconstruct the cognitive history of the human self is driven by our intrinsic curiosity about the origin of what makes us human and what sets us apart from our closest relatives, whether alive or extinct. Ultimately, our neurocognitive capacities have driven tool use and technologies, the emergence of complex societies, creative expression, human culture, and even the evolution and prevalence of psychiatric disorders. However, deeper insights into the evolution of specific cognitive traits are limited by the fact that ancestral brains are inaccessible to experimental or even archeological exploration.

Over the last decade, evolutionary anthropology has adopted a bottom-up approach, typically based on studies of archeological records, animal and organoid models, and genetic variants of extant and ancient organisms. Integrating evolutionary genetics with archeological evidence, particularly through the analysis of events spanning over 60 million years, has provided molecular timelines illuminating the origins of humanity as well as nuanced understanding of human evolution (Piszczek et al., 2023). This approach is especially powerful when combined with paleogenetic data from extinct human relatives, such as Neanderthals (Zeberg et al., 2024) and Denisovans (Green et al., 2010; Peyrégne et al., 2024). By comparing genome sequences across different organisms and hominin lineages, we can understand their molecular relationships (The Chimpanzee Sequencing and Analysis Consortium, 2005) and identify genetic variants and mutations that influence gene expression through copy number variations or cis-regulatory elements (Pollen et al., 2023). These analyses have revealed complex patterns of separation and interbreeding among hominin lineages, which are associated with their migration patterns, habitats, and archeological footprints (Skoglund and Mathieson, 2018).

Comparative genomic approaches have pinpointed specific genetic modifications associated with significant developments in brain structure and function, revealing the evolutionary pressures that have shaped the human brain (Dorus et al., 2004). Computational methods, such as PrediXcan, enable the identification of regulatory differences in primate brains using regression models applied to genotype-tissue expression (GTEx) project data (Colbran et al., 2019). Recent advanced computational approaches offer increasingly integrated views: comparative neuroscience datasets allow for the imputation of ancestral cortical structures, modeling of cortical evolution, and computational retracing of evolutionary adaptation to different environmental demands (Schwartz et al., 2023).

Together, archeology, genetics, organoid and animal models offer strong mechanistic insights and specific snapshots of human brain evolution, such as the mechanisms underlying brain size and cellular composition. However, a comprehensive reconstruction of the human mental past has proven to be a challenging task using either comparative neuroscience approaches alone (through analysis of endocasts or functional neuroanatomical extrapolations) or neurogenetics alone (by examining single-gene mutations influencing human traits, such as brain expansion and language capacity) (Piszczek et al., 2023). Thus, the field faces a critical gap: current methods excel at revealing isolated evolutionary changes but struggle to provide an integrated, systems-level understanding of how multiple genetic and brain network changes collectively shape human cognitive evolution.

### 1.4 From brain networks to ancestral selection

We explored a complementary strategy enabling the holistic reconstruction of ancestral functional brain states in a top-down manner, drawing from multigenic effects across cognitive traits. A recent pilot study (Kaczanowska et al., 2022) laid the groundwork by fusing publicly available multimodal brain data. This approach operates on two levels: first, a data-driven computational level operating on present-day human data, integrating genetic and functional information with evolutionary features; second, an evolutionary interpretation level relating the identified patterns to potential ancestral selection processes. This study used substitution ratio (*ω*) values and likelihood estimates of non-synonymous versus synonymous coding sequence changes as gene-based proxies of ancestral selection within a phylogenetic framework (Yang, 1998). These ω-values are computed from protein-coding DNA sequence (CDS) information from databases such as Ensembl, OMA, and ancient genome repositories, using computational tools such as *codeml* packages (Yang, 2007). The selection pressure values were projected gene-wise onto brain space according to expression sites from the Allen Human Brain Atlas (AHBA) (Hawrylycz et al., 2012), yielding brain-wide maps of ancestral selection pressure. This approach, used in imaging transcriptomics (Mandal et al., 2022), enables spatial mapping of genetic attributes onto the brain architecture. This spatially explicit selection dataset was subsequently fused with gene-wise brain-wide correlations to task-related functional brain networks (FNs) derived from the HCP data, with registration performed in the AHBA template space. This essentially bimodal data matrix, consisting of temporal genetic (evolution) and the correlation of the spatio-functional (FNs) domains to the gene expression, is mined in a straightforward manner using biclustering algorithms. Therefore, at a more abstract level, we correlated the genetic information *G(g,s)* for every gene *g* and biopsy site *s* to the functional information *F(n,s)* for every network *n* and biopsy site *s*, and concatenated it with information on genetic pressure *ω(g,b)* for gene *g* and key mammalian species *b*. On this matrix, we find biclusters as subsets of genes, branches, and networks. Although not used in brain research, this class of unsupervised learning methods is advantageous for uncovering patterns within complex, two-dimensional datasets (Cheng and Church, 2000). We specifically chose a customizable Genetic Algorithm for Biclustering (GABi) for our approach (Curry, 2014). In our context, the resulting biclusters represent gene sets that co-evolve, share similar *ω* selection profiles across evolutionary branches, and are co-expressed in spatial patterns that align with a specific functional brain network. By applying this algorithm, traces of ancestral selection pressures acting on cognitive functions can be systematically uncovered. Each identified bicluster links a cognitive function (via its associated FN) to a particular evolutionary time point and gene set, enabling the computational reconstruction of when and how specific traits, such as language capacity or strategic cognition, emerged within human ancestry.

#### 1.4.1 Proposed workflow and enhancement

**Figure 1:**
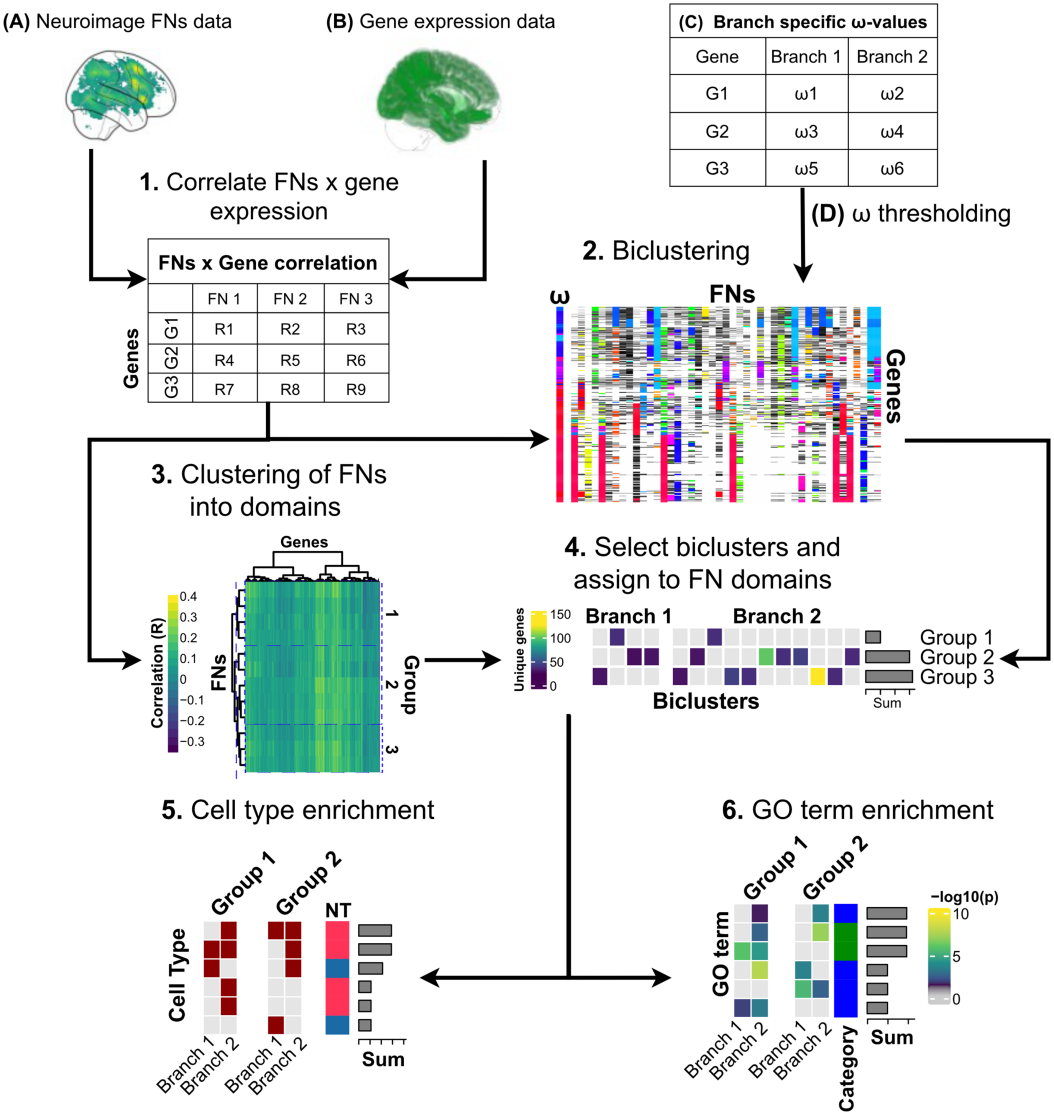
Workflow for mapping multigenic evolution across the cognitive, cellular, and molecular levels. The integrated pipeline processes three types of input data: **(A)** functional networks (FNs) derived from selected neuroimaging data sources (Neurosynth, HCP, Neurovault), **(B)** spatially resolved brain gene expression maps (utilizing ABA), and **(C)** branch-specific *ω*-values for selected gene sets. The workflow comprises six sequential steps that can be customized: (1) correlating FNs with regional gene expression; (2) employing GABi biclustering to identify coordinated gene-FN-evolution associations; (3) clustering FNs into groups (domains) concurrently; and (4) selecting significant biclusters through cluster-thresholding across phylogenetic branches. The resulting biclusters can then be analyzed in terms of: (5) cell-type enrichment, and (6) gene ontology term enrichment. Key workflow steps (1–6) and data sets **(A–D)** can be adapted to address specific scientific questions, for example, **(A)** integrating various fMRI databases (Neurosynth, HCP, Neurovault) for broader FNs coverage, (1) employing different correlation methods between FN profiles and gene expression patterns for specific network focus; **(C)** applying various methods and constraints for estimating gene evolutionary pressure for diverse evolutionary coverage; and **(D)** thresholding such estimates for tailored gene set selection, (2) exploring various GABi parameters for enhanced bicluster discovery, and (4) determining the bicluster selection criteria. Steps 5. and 6. can be adapted by using different sources for cell type and GO term enrichment analyses. Importantly, step 3 could be omitted, and the biclusters could be analyzed at a single FN level in the downstream analysis (steps 5–6).

In this context, we refined the aforementioned method for data mining and explored both the evolutionary temporal aspect and the functional spatial network dimension. Here, we present several key aspects to increase the applicability of this workflow across a broader spectrum of research fields and enhance its usability for the neuroscience community:

i) We adapted multiple aspects of the pipeline (Figure 1) to improve the overall accessibility and usability. To this end, we developed an integrated computational workflow with clearly defined instructions and a comprehensible code to jointly handle genetic data, brain gene expression maps, and fMRI connectivity data as inputs.
ii) We modified the handling of genetic data to enable the integration of a larger, more comprehensive gene set into the analysis pipeline, particularly by employing less conservative assumptions regarding incomplete *ω* data.
iii) We adapted FN handling to accommodate larger, user-defined task sets derived from synthetic imaging databases (such as neurosynth.org, Yarkoni et al., 2011), enabling greater flexibility in selecting cognitive domains for analysis.
iv) We enhanced the mining of the multimodal data space via the GABi algorithm through parameter optimization to ensure the robust identification of biologically and evolutionarily meaningful biclusters.
v) Finally, we added a specific structured output for adaptive evolution and purifying selection at the FN, cellular, and molecular levels, facilitating interpretation across biological scales.

Applying this optimized workflow to the evolution of socio-affective traits, we identified specific peaks in adaptive selection affecting a subset of higher-order socio-affective trait networks in anatomically modern humans (AMH). In summary, we demonstrate the versatility of our approach by targeting socio-affective brain networks and offering novel computational means for jointly assigning genetic and brain imaging data within the context of psychiatric and population genetics research. This framework provides a generalizable strategy for not only reconstructing the evolutionary forces that have shaped human brain organization and cognition, but could be expanded to other neuroscientific fields.

## 2 Materials and methods

### 2.1 Task-evoked functional brain activity data *F(n,s)*

In our pilot study (Kaczanowska et al., 2022), functional magnetic resonance imaging (fMRI) data were obtained from the Human Connectome Project (HCP), including gambling, emotional processing, and working memory. Here, we took advantage of the synthetic database, NeuroSynth (neurosynth.org, (Yarkoni et al., 2011)) as a source for task-related functional network representation (Figure 1A). The Neurosynth database allows for the straightforward assembly and retrieval of user keyword-defined task-related functional network information. Note that any other reference database (e.g., HCP, OpenfMRI) would work similarly. To focus on socio-affective functions, *e* obtained “uniformity test maps” for 45 *a priori* selected terms (see Supplementary Table 1) being the set of functional networks $N:=\left\{n_{1}\mathrm{,,}\ldots \mathrm{,,}n_{45}\right\}$ serving as input for the following steps. Uniformity test maps were provided as z-scores sampled in a discrete Montreal Neurological Institute (MNI) 152 2 mm space. These statistical inference maps scores indicate whether activation patterns in a given voxel were more or less frequent than expected by chance, based on the overall distribution of activations in the brain (see (Yarkoni et al., 2011) for details). Activation pattern visualization was performed using the *plot_glass_brain* function from the Nilearn package (Nilearn Contributors et al., 2025) using Python version 3.9.12.

The original method from (Kaczanowska et al., 2022) was upgraded to improve the way the network values *F(n,s)* were derived at the 3702 biopsy sites *s* of the gene expression dataset from the Allen Human Brain Atlas (see below)for each of the 45 functional networks *n*. Specifically, the radius for mapping the FN signal onto each biopsy site was contingent on the density of the biopsy sites in its region: if the distance for a given biopsy site to its nearest neighbors was equal to or below 5 of squared Euclidean distance, the direct z-value from the closest MNI point was taken; otherwise, a Gaussian-weighted normalization was applied (with *μ* and *σ* set to 0 and 20, respectively) to the z-value. This ensured that in areas with closely spaced biopsy sites, a smaller radius was used to prevent overlapping effects, whereas in regions with fewer biopsy sites, a larger radius was employed. This improvement over the previous approach allowed the biopsy site space to be filled with representative FN signals, particularly in areas where the biopsy sites were widely spaced.

### 2.2 Spatial gene expression data *G(g,s)*

Spatial gene expression data (oligo microarrays) for human protein-coding brain-expressed genes were reused from our pilot study (Kaczanowska et al., 2022), specifically Supplementary Data S6 (all_genes_expression_Human.mat). These data (Figure 1B) include expression information *G(g,s)* for the 8977 genes g at the 3,702 biopsy sites in the brain from the Allen Human Brain Atlas (AHBA) (Hawrylycz et al., 2012). These values were used in Section 2.4.1.

### 2.3 Substitution rate ratio data *ω(g,b)*

To explore the evolutionary path of brain-expressed protein-coding genes, we employed a dataset previously curated and calculated by Kaczanowska et al. (2022), specifically in Supplemental Table S2 and Supplemental Data S6. This dataset includes one-to-one orthologs for 8,977 genes *g* across key mammalian species *b* within the AHM phylogeny, as well as raw ω-values (Figure 1C). These ω-values *ω(g,b)* represent the maximum likelihood estimates for the ratios of nonsynonymous (dN) to synonymous substitutions (dS) in a gene’s coding sequence along a specific phylogenetic branch. In brief, Kaczanowska et al. obtained homologous coding sequences from the OMA orthology database or ancient genome repositories, filtering for the presence of gene expression in AHBA. They reconstructed phylogenetic trees and timelines from mitochondrial DNA (mt-DNA), and calculated raw *ω*-values using the codeml routine implemented in PAML v4.9i (Yang, 2007), see, e.g., https://github.com/abacus-gene/paml/wiki/Substitution-models#nucleotide-substitution-models for a detailed description. In the previous study by (Kaczanowska et al., 2022), these raw ω-values were conservatively filtered (Table 1, 2nd Column). To enhance the discovery potential of our approach, we relaxed three of the constraints to include a broader range of genes, particularly in recent branches (Table 1, 3rd Column; Supplementary Figure 2B).

**Table 1:** Handling constraints for ω-values.

PAML outcome	Conservative constraints (Kaczanowska et al., 2022)	Relaxed constraints (this study)	Evolutionary interpretation
Stable ω > 1	Value kept	Value kept	Adaptive selection
Stable ω 0–1	Value kept	Value kept	Neutral evolution
Stable ω ~ 0	Value kept	Value kept	Purifying selection
Unstable ω in 5 runs	Filtered out	Filtered out	Filtered out
Short edge, unstable	Filtered out	Filtered out	Filtered out
dN/dS = 0, (dN = 0)	NA	Set to 0	Purifying selection
dN/dS = ∞, (dS = 0)	NA	Set to maximum value of the species/branch	Adaptive selection
Identical sequences	NA	Set to 0	Neutral evolution

PAML predictions yield different outcomes (first column), which can be filtered and clipped pending conservative (second column) or more relaxed (third column) constraints. High, intermediate, or low ω-values can then be interpreted as signatures for adaptive selection, neutral evolution, or purifying selection, respectively (Jeffares et al., 2015). Importantly, these constraints operate on the original PAML output (here taken from Kaczanowska et al., 2022), and can be set by the researcher pending their specific use cases (e.g., when operating with different phylogenies, gene (sub)sets, etc.…).

The underlying phylogenetic tree and timeline were obtained from (Kaczanowska et al., 2022). In brief, we previously used an AMH-chimpanzee mean divergence of 7.8 Mya (SD 1.2 Mya), an Old World monkey-ape divergence of 28 Mya (SD 3 Mya), and a 60 Mya mean (SD 2.8 Mya) for the coalescence of the primate lineages. A maximum clade credibility tree was generated using the software Figtree 1.4 (Rambaut, 2018). The mt-derived phylogeny outcomes aligned with the primary gene tree topology and were used to annotate the timelines. The mt-DNA-derived phylogeny and main gene tree topology position Neanderthals closer to AMH (Meyer et al., 2014) and support the Chimp-to-Denisovan-Neanderthal-AMH species sequence used here. Note that the workflow (Figure 1) can be used with any other phylogeny and genetic feature other than the ω-value and the specific ω-table presented here.

### 2.4 Pattern mining through biclustering

#### 2.4.1 Correlating gene expression and functional networks

To identify genes linked to specific tasks or FNs, we mined co-evolving genes with high spatial correlations with these networks. As described above, we retrieved anatomically modern human (AMH) spatial gene expression data *G(g,s)* from 3,702 biopsy sites in the Allen Human Brain Atlas (ABA) for each of the 8,977 genes (see Section 2.2. Spatial gene expression data). Next, we set the network data *F(n,s)* of the 45 socio-affective networks (see 2.1. Task-evoked functional brain activity data sets) in context to the gene expression data *G(g,s)*: we did this by computing the Spearman’s rank correlation coefficients r_ij_: = *r*(*g*_i_,*n*_j_) = *ρ* (rank(*G*(*g*_i_,*s*)), rank(*F*(*n*_j_,*s*)) over the 3,702 biopsy sites *s* between every gene *g*_i_ and network *n*_j_ (Figure 1, Step 1) using the function “cor” from the “stats” packages in R with option “pairwise.complete.obs” (computation for all complete pairs) and the method “spearman.”

**Figure 2:**
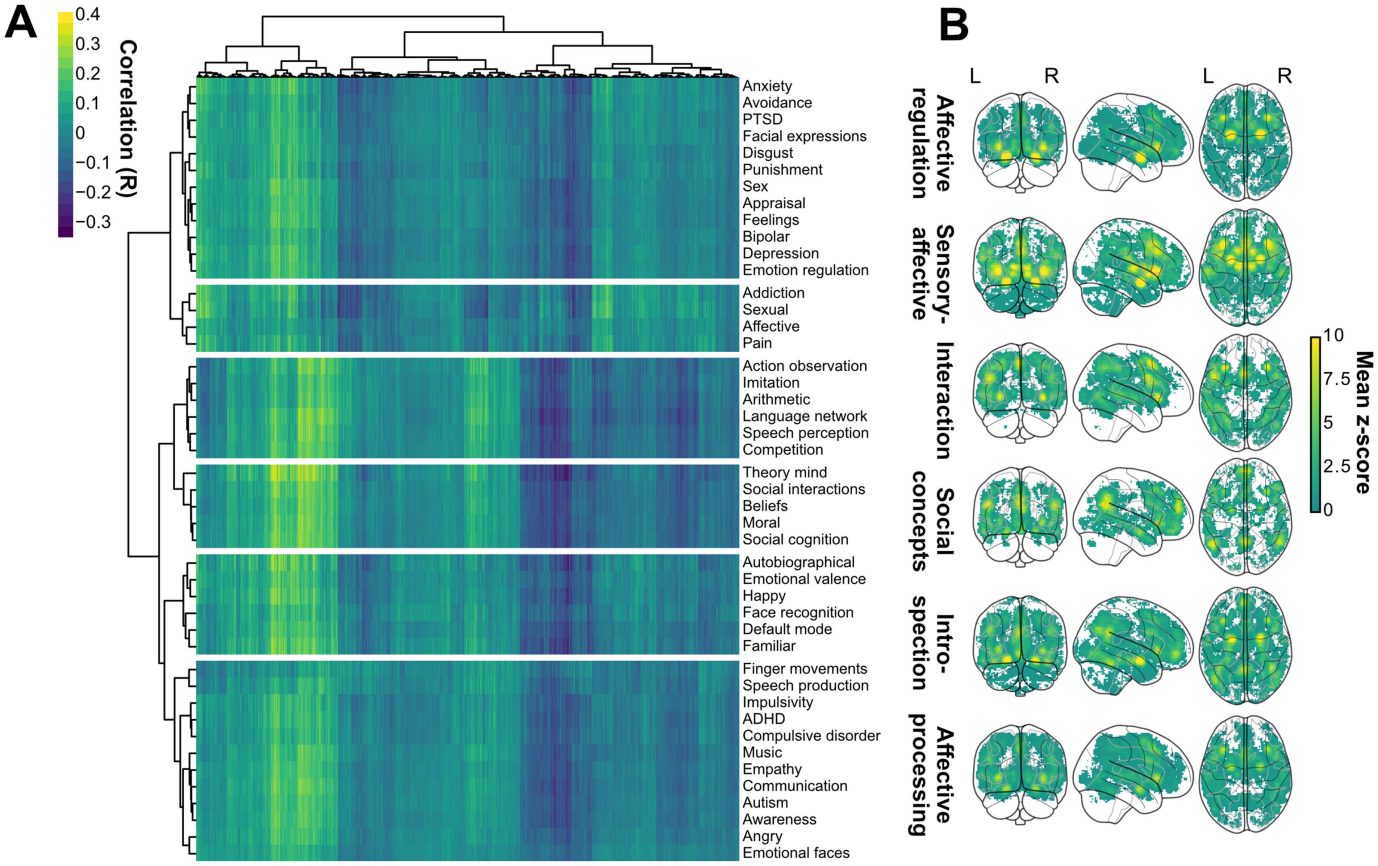
Data-driven classification of Neurosynth terms into socio-affective domains. **(A)** Hierarchical clustering of Neurosynth socio-affective FNs and ABA gene expression correlations across biopsy sites revealed six distinct socio-affective domains. **(B)** Brain-wide representation of the six socio-affective domains showing their spatial distinction. For each domain, a mean uniformity test z-score task-fMRI signal across the domain-specific Neurosynth terms was calculated and plotted using nilearn. Note that such data-driven clustering can be adapted to various combinations of Neurosynth terms or other task-fMRI datasets.

Next, to organize the FNs into meaningful socio-affective domains, a hierarchical clustering analysis was conducted on rows of the *r(g,n)* = ((*r*_ij_))*i*=1,...,45,*j*=1,...,8977) matrix, using the R implementation of Ward’s minimum variance method using squared Euclidean distance as the distance measure algorithm (Figures 1,2, Step 3; Supplementary Figure 1). This method was selected because of its effectiveness in minimizing within-cluster variance, which ensures that the resulting clusters are as cohesive and distinct as possible. This allowed grouping different individual FNs into overarching social and emotional domains for initial gross interpretation. The optimal number of clusters was selected using the silhouette average width method using *silhouette* function from *cluster* package in R. The overlap between the individual FNs and socio-affective domains was determined using both the Pearson correlation coefficient (using the *pearsonr* function from the *scipy* package (Virtanen et al., 2020) in Python version 3.9.12) and the Dice coefficient (using a self-written function using the *numpy* package (Harris et al., 2020) in Python 3.9.12, where the Dice coefficient 2*|A∩B|/(|A| + |B|), for A and B FNs signal patterns). The latter is a measure of the extent of overlap between the thresholded activation maps (Wilson et al., 2017). A Dice coefficient value of 0 implies no overlap at all between the given FNs activations, whereas a value of 1 implies perfect overlap. This gross interpretation can be extended to individual FNs within these clusters for a more detailed dissection. The activation patterns for the overlaps, differences, and mean signal in the socio-affective domains were visualized using the *plot_glass_brain* function from the Nilearn package (Nilearn Contributors et al., 2025) using Python version 3.9.12.

#### 2.4.2 Data preparation for biclustering

To calculate the evolutionary signatures within the AMH lineage, we used the aforementioned table *ω(g,b)* comprising 8,977 genes *g* (rows) and 11 branches *b* (columns); see (Kaczanowska et al., 2022), with a similar data processing workflow. In the first step (Figure 1C), the *ω*-values were rank-normalized for each branch (rank/number of genes), excluding undefined ω (dS = 0) values (Villanueva-Cañas et al., 2013) using the function “rank” in R with ties method “min”: let $\omega_{ij}:\;=\;(g_{i},b_{j})\;\mathrm{and}\;{\tilde{\omega }}_{ij}=\frac{1+|\left\{k\neq i:\omega_{kj}<\omega_{ij}\right\}|}{\left|\left\{k:\omega_{kj}\neq NA\;and\;\omega_{kj}>0\right\}\right|}$ its rank normalization Specifically, the $\tilde{\omega }$ -values approaching 1 correspond to the largest *ω* for a given branch, whereas rank-normalized $\tilde{\omega }$ -values near zero indicate the smallest values. Next, the full correlation matrix *(g,n)* was filtered for genes whose correlation with at least one network exceeded the selected threshold > 0.75. Similarly to the previous workflow, we pre-selected genes for each branch (Branch 1 to 7, Chimp, Denisovan, Neanderthal, and AMH) in the evolutionary tree from either high (>1) or low *ω* (~0) categories and harmonized the pool sizes of these high-*ω* and low-ω gene sets across branches.

Concurrently, and in accord with the previous approach, the fMRI-to-gene expression correlation matrix *r(g,n)* (here 8,978 genes as rows and 45 FNs as columns, Figure 1 Step 1) was rank-normalized to $\tilde{r}$ , with a range between 0 and 1 with gene-to-network values with the lowest correlations set to zero and those with the highest correlations set to 1 using the function *rank* in R with ties method “*max*”: $\;\tilde{r_{ij}}:=\frac{1+|\left\{k\neq j:r_{ik}<r_{ij}\right\}|}{\left|n\right|}$ . Subsequently, we set the minimum value per gene and the values for genes with a low overall correlation with all networks (i.e., <0.1) to 0.

Finally, we concatenated the normalized fMRI-to-gene-expression correlation matrix $\tilde{r}$ *(g,n)* with the table of normalized ω-ranked genes $\tilde{\omega }$ *(g,b)* for each evolutionary branch under investigation to the matrix [ $\tilde{\omega }$ *(g,b)* $\tilde{r}$ *(g,n)*].

#### 2.4.3 Subspace pattern mining for network evolution via biclustering

The center of our proposed workflow is the use of the customizable biclustering algorithm GABi (Curry, 2014) to mine the ω-fMRI-to-gene-expression data table [ $\tilde{\omega }$ *(g,b)* $\tilde{r}$ *(g,n)*]. Figure 1 Step 2, which was concatenated in the previous steps (2.4.2 Data preparation for biclustering). Here, we updated the previously used genetic algorithm pipeline (Kaczanowska et al., 2022) on several levels with respect to (1) input data, (2) adding an option for branch-specific *ω*-rank thresholds (Figure 1 Step 2; Supplementary Figure 2A), (3) improving the optimization strategy for finding biclusters, and (4) adapting the post-processing pipeline.

In terms of data input, we relaxed three constraints in the previously calculated ω-values, thereby enabling the processing of a broader range of genes (Table 1; Supplementary Figure 2B). To address this issue, a modification was introduced to the GABi pipeline, wherein thresholds were iteratively computed for each branch independently, setting branch-specific minimum and maximum *ω* -rank thresholds (Figure 2; Supplementary Figure 2A).

We improved the optimization strategy as follows: GABi uses a genetic algorithm to search for optimal biclusters. We define a bicluster as *B =* (*g*_sub_, [*b_sub_ n_sub_*]) with a sublist *g_sub_* of all 8,977 genes *g* (*g_sub_ ⊆ g*), a branch *b_sub_* of all 11 key mammalian species *b* (*b_sub_ ⊆ b*) and a list of networks *n_sub_* of all 45 networks *n* (*n_sub_ ⊆ n*). The algorithm has several customizable parameters related to the exploration of the fitness space (Supplementary Figures 2C,D); for a more detailed description, see (Curry, 2014). Genetic algorithms incorporate randomness into the initial part of the bicluster finding. Therefore, iteration steps are generally beneficial for increasing the possibility of finding a global optimum. We adapted the relevant parameters such as the amount of iterated bicluster searches “amountOfBiclusterSearches,” the number of isolated sub-populations “nsubpops” and the number of candidate solutions “popsize.” A higher value for the parameter “amountOfBiclusterSearches,” which defines the number of iterations of GABi, will increase the computation time but also lead to a higher number of found biclusters. This also holds for the parameter “popsize,” which defines the number of candidate solutions. The parameter “nsubpops” defines the split of the search space into subpopulations and leads to higher computational performance but less optimal solutions. These parameters were adapted to 20, 4, and 250 for (high-*ω*/1,200 for low-ω) gene sets, respectively. We also have the parameter “do_weighting,” which defines whether ω and the network table are weighted differently (can be True or False), and the parameter “variableClassNotOnTabuList,” indicating whether already found biclusters are set to zero by adding them to the tabu list, which can lead to more diverse solutions. To increase the chance of finding interesting solutions, we added the option “global_optim” (Supplementary Figures 2C,D), which looped for a given number of iterations (ideally set to a multiple of 4) through several combinations of these parameters “do_weighting” (set to True or False) and “variableClassNotOnTabuList” (set to (True, True) or (True, False) for (ω, n)). Generally, all parameter values were obtained heuristically, based on the parameter description given in (Curry, 2014) and iterative experiments, aiming to obtain a diverse set of clusters while considering the computational time. Finally, during each iterative run, several biclusters can be found with a substantial overlap between the runs. Therefore, we merged similar biclusters and recomputed the scores (=size) of the merged clusters. Biclusters were defined as similar, if their genes overlapped by more than 50% (normalized by smaller size) or had the same networks similar to the published approach (Kaczanowska et al., 2022).

#### 2.4.4 Data post-processing

**Figure 3:**
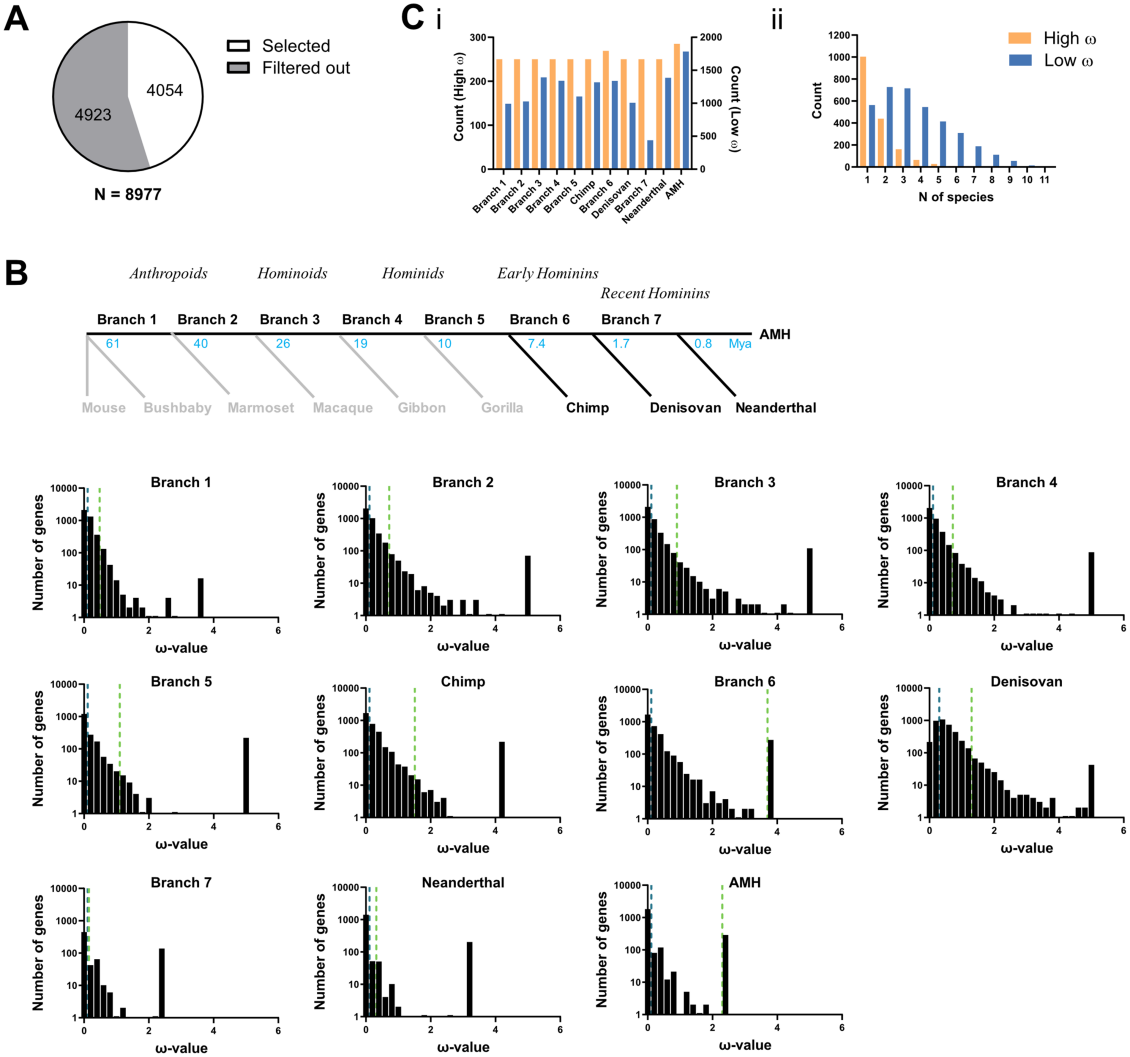
Tracing genetic selection in human phylogeny. **(A)** Spatial filtering. Full branch x gene ω table, filtered for spatially informative genes whose correlation to the fMRI networks exceeds a selected threshold at least once (specifically, R > 0.1). This left 45.1% of genes for tracing evolution in fMRI networks. **(B)** *Top*: Human phylogeny. Black lines indicate ancestral branches in human (AMH, Denisovan, and Neanderthal) ancestry used for computational predictions. Gray lines indicate branches not considered in this study (adapted from Kaczanowska et al., 2022). *Bottom*: Distribution of ω-values across branches of the selected brain-expressed genes. Dotted lines indicate the criteria for high (light green) or low (dark green) ω gene sets. **(C)** Evolutionary ranking. Filtered genes **(A,B)** that are either in the high and low ω gene sets per branch (i) and the number of times a gene was present in the high or low ω groups across the branches (ii). Note that the choice of gene selection is highly customizable, as step A can be omitted or set at different R thresholds. In addition, the branch-wise ω thresholds (step B) can be set more stringently or relaxed, and/or include additional splits (i.e., middle ranged ω genes) for further contrasts.

**Figure 4:**
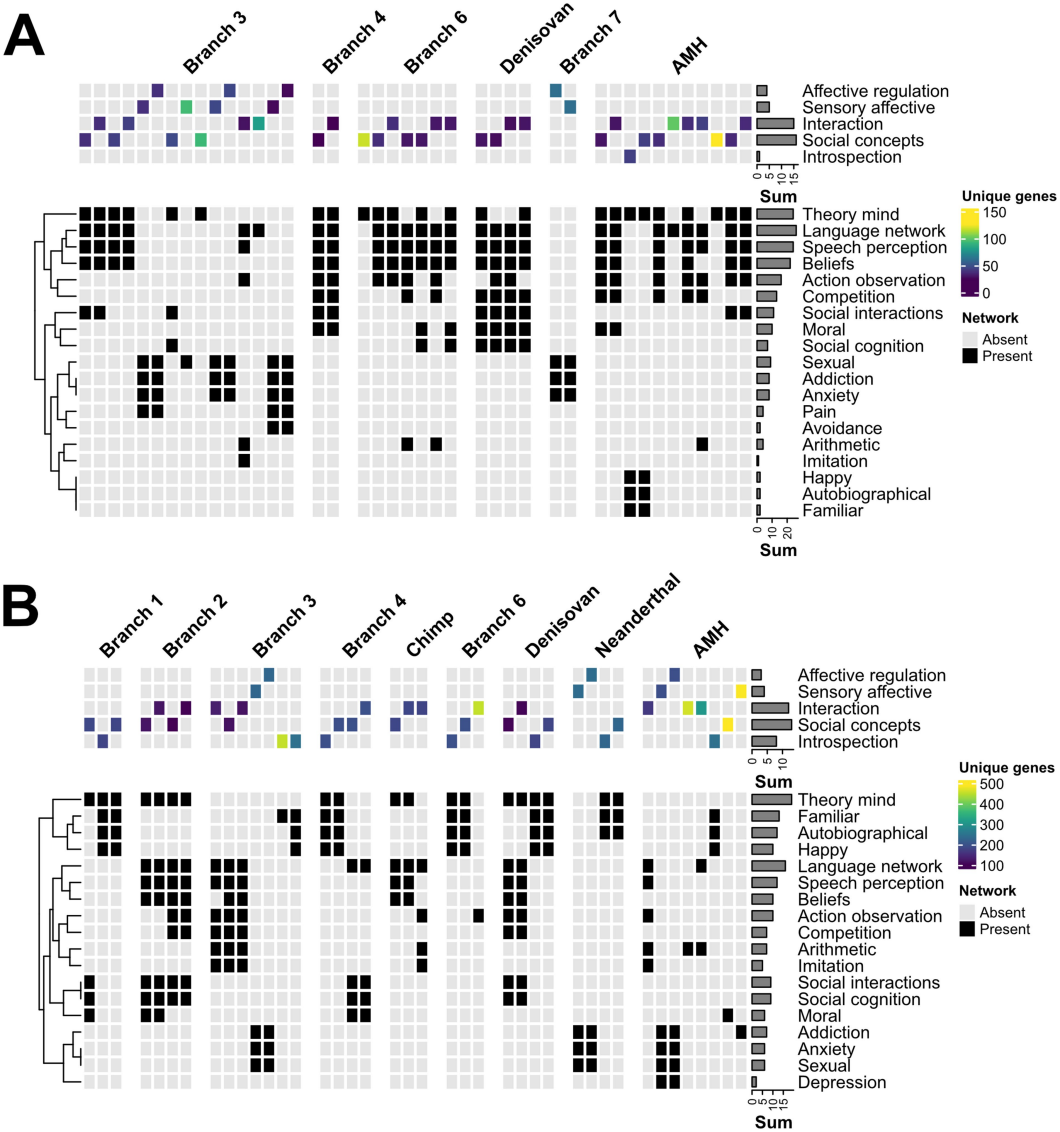
Mining the human socio-affective task space for evolutionary selection. Top 40 biclusters from GABi for either high ω **(A)** or low ω **(B)** gene input. *Top*, Identity and branch-wise number of unique genes within the biclusters of the main socio-affective domains. *Bottom*, Neurosynth network composition for each bicluster. For an extended representation, refer to Supplementary Figure 3. The biclusters assigned to Affective processing domain did not reach the selection threshold in either gene input case.

For further analysis, we chose either the top 40 overall (Figure 3) or the top 20 biclusters (Figure 4) per branch according to their bicluster scores. Next, each bicluster was assigned to the respective socio-affective domain (as shown in Figure 1, Step 4), depending on the FNs composition of the bicluster. If a given bicluster FNs composition spanned more than one domain, it was assigned to all the belonging domains.

### 2.5 Single-cell gene expression for cell-type enrichment

For cell-type enrichment, we relied on a previously published cell-type mean gene expression matrix at the cluster level, with 461 cell clusters present in the dataset (Siletti et al., 2023). Next, we matched the genes of this data with the *ω*-ranked table, generating an 8,977 × 461 gene-to-cluster expression matrix. For every column, we set the gene expressions that were not in the top 90 percentile to 0 to filter out noise and focus only on a clear signal with highly expressed genes. These data were then related to the branch and socio-affective domain-specific gene sets obtained from biclustering using the correlations (Figure 1 Step 5; Supplementary Data 1). Next, we normalized the correlation values across all cell types/clusters (z-scored) within a given branch to identify the cell types with the highest representation in each branch. A cell type was classified as enriched for this branch with a critical z-score of Z > 1.96. Finally, the data were collapsed for presence in a supercluster, auto-annotated class, and auto-annotated neurotransmitter (see Siletti et al., 2023 for details). The neuronal and non-neuronal cell types were split for plotting.

### 2.6 GO annotation analysis

To identify the biological pathways and processes associated with the GABi-derived biclusters (Figure 1 Step 6), we utilized the Metascape tool (Zhou et al., 2019) on cumulative, unique gene lists for a set of biclusters belonging to each group. All biclusters belonging to high and low ω-ranked genes, branches, and each socio-affective domain were run separately. Specifically, for each iteration, we selected annotations for “Biological Processes,” “Cellular Component,” and “Molecular Function” within the “Function/Location” category. In the “Membership” section, we further refined the analysis to include genes associated with specific Gene Ontology (GO, (Ashburner et al., 2000)) categories: “Molecular Functions,” “Biological Processes,” and “Cellular Components.” Similarly, during the “Enrichment” selection, we opted to analyze the enrichment of these same GO categories, with a *p*-value cutoff set at 0.05 and minimal enrichment at 1.5. Finally, we selected only the top 10 terms (as defined by the highest -log_10_(*p*) value) per branch and socio-affective domain (Supplementary Data 2).

## 3 Results

### 3.1 Improving user-defined import and preprocessing of fMRI task space

The primary objective here was to develop on our initial strategy (Kaczanowska et al., 2022) into an embedded workflow, improve its core function and extend its versatility to new datasets, going beyond the cognitive-focused FNs offered by HCP data. The core of this workflow relies on a bilcustering genetic algorithm (GABi) for data mining and pattern search, which facilitates the exploration of both the evolutionary temporal dimension and the functional spatial network domains.

To increase the versatility and ease of access of our initial strategy (Kaczanowska et al., 2022), we first incorporated the integration of user-defined fMRI datasets into our workflow. The Neurosynth database (Yarkoni et al., 2011) allows for the straightforward assembly and retrieval of user keyword-defined task-related functional network information [note that any other reference database (e.g., 1,000 Functional Connectome Project)^1^ will work similarly]. Here, we selected task-specific brain activity maps related to socio-affective processing, a trait domain that has been extensively discussed in evolutionary anthropology, from the Neurosynth database (see Methods and Supplementary Table 1). The Neurosynth output data were merged in the MNI coordinate space with spatial gene expression data from the Allen Human Brain Atlas brain biopsy sites (Hawrylycz et al., 2012) using an adapted custom script (see Methods).

**Figure 5:**
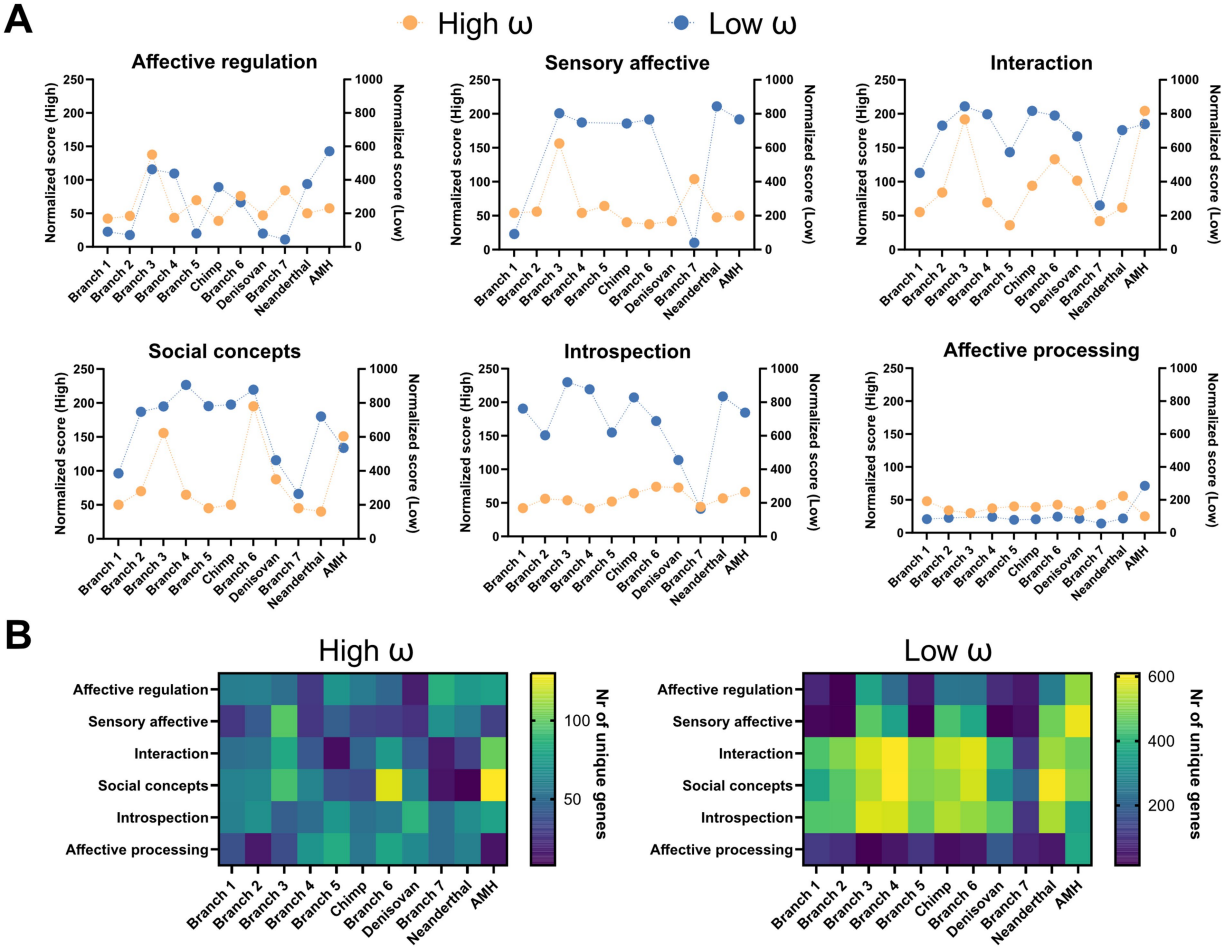
Evolutionary selection across socio-affective domains. **(A)** Top 20 biclusters per branch grouped into previously obtained socio-affective domains. Normalized score from all detected biclusters across each socio-affective domain. **(B)** Number of unique genes in all detected biclusters for a specific branch and socio-affective domains. For individual biclusters, see Supplementary Figure 3. This analysis can be adapted and optimized in the future by including (i) more biclusters detected by the genetic algorithm, (ii) focusing only on selected subsets, for example, the top 20 per branch as presented here, or (iii) applying even more stringent selection criteria.

Next, to increase the interpretability of larger fMRI datasets, we grouped the fMRI FNs into functional domains. This allows for a more straightforward initial interpretation of overall functional selection. Here, brain-wide gene expression profiles were correlated with 45 Neurosynth-derived fMRI FNs. We then grouped the task-specific brain activity maps with similar genetic correlation patterns using a hierarchical clustering. This step allowed for a more straightforward interpretation of the results. Here, we identified six functionally and spatially distinct socio-affective domains (Figures 5A,B, respectively; Supplementary Figure 1). These domain names (Figure 5A) reflect the characteristics and functions of the underlying FNs and serve as a basic set of categories to simplify the interpretation of human social-affective traits throughout the study. This gene matrix and the clusters served to subsequently compute and interpret the data, respectively.

### 3.2 Reconstructing ancient traits from the evolutionary genetic × fMRI dataspace

Evolutionary forces shape genomes and phenotypes through protein-coding mutations, including non-coding RNA modifications, regulatory sequence alterations, indels, species-specific gene duplications, and copy-number variations. These diverse changes ultimately underlie the same organism-level selective pressures and allow an approximation of evolutionary traits from homologous brain-expressed protein-coding genes as proxies. In our case, this reliably reflects the evolutionary selection pressure over time and is particularly useful for detecting adaptation in cases where population data are scarce, such as with extinct species. These coding sequences can be used to compute *ω*-values as a measure of evolutionary selection pressure with ω of >1 (high ω) and ~0 (low ω), indicating adaptive and purifying selection (Yang, 1998), respectively (Yang, 1998). The selection pressure on multigenic traits can then be approximated by combining ω-values across several genes (gene sets) sharing high or low ω-values; for instance, as cumulative ω-values across genes expressed in a given brain region.

To capture a broader range of genetic variability, we first relaxed the constraints of the ω-estimates of the initial dataset (Kaczanowska et al., 2022). This optional step increased the number of genes available for subsequent analyses (Supplementary Figure 2B). It also makes the approach more sensitive and comprehensive, and offsets the lost precision in *ω*-values of the subset of genes (Table 1). The genes in each branch were ranked and pre-selected according to their ω-values (Figures 2A,B, see Methods). As expected, there was an overall bias toward purifying selection in brain genes (Somel et al., 2014), indicated by a higher number of low than high-*ω* genes (Figure 2Ci). Interestingly, low-ω genes were distributed over a significantly broader set of branches than high-ω genes. This might reflect a constant background of purifying selection, interspersed with bouts of adaptive innovation driven by branch-specific sets of high-*ω* genes (Figure 2Cii).

Next, we used these gene sets to highlight evolutionary hotspots in the brain FN space. Here, we employed an updated version of the customized version of GABi (Kaczanowska et al., 2022), which iterates through the evolutionary genetic × FN space to identify large biclusters grouping co-evolving high or low-ω gene sets with a correlation to specific FNs. One optimization targeted the bicluster search: GABi uses a genetic algorithm (GA). GAs generally employ heuristic search techniques and depend on several tunable parameters for effective exploration of the search space. Without proper tuning, they may become trapped in local minima or require excessive computational resources (Curry, 2014). To address this, we optimized the bicluster search parameters, allowing for the exploration of a broader solution space while still considering the computation time (Supplementary Figure 2). Additionally, we applied biclustering separately for every branch with a branch-specific *ω*-rank threshold. Owing to the substantial variation in the distribution of ω-values across branches (Figure 3B), we expected that a fixed threshold (global) would lead to the under-detection of biclusters in branches with fewer positive ω-values (Supplementary Figure 2A).

The top 40 biclusters from the biclustering across all analyzed branches revealed distinct patterns within and between the high- and low-*ω* gene sets (Figure 3). Overall, the number of biclusters (total matrix count) was higher for high-ω (195 across six branches) than for low-ω (166 across nine branches), reflecting broad “exploration” in adaptive evolution and focused purifying selection of fixed traits in the latter group. The row counts highlight Language, Speech, Theory of Mind and Beliefs, as well as Interaction and Social concepts, as the evolutionarily most relevant FNs and socio-affective domains, respectively. A more detailed inspection revealed prominent biclusters: for Language in Branch 6, in line with earlier findings (Kaczanowska et al., 2022); for Theory of mind present more focused in AHM, while early hominoids (Branch 3) are characterized by more broadly distributed biclusters (Figure 3A). In contrast, low-ω clusters appeared in Branches 1 and 2, indicating early purifying selection within these FNs (Figure 3B).

We then looked at the bi-cluster distribution in all branches equally by selecting the top 20 biclusters from each of them (Figure 5; see Supplementary Figure 3 for individual biclusters). At the network level, the overall trends of socio-affective adaptive evolution and purifying selection can be readily visualized by the cumulative cluster scores over these branches and FNs (Figure 4A). This uncovered striking traces of ancient selection: a strong signature of adaptive evolution (high-*ω* traces) shaping high social functions, Interaction, and Social Concepts during hominoid (Branch 3), early hominin (Branch 6), and AMH, which is less apparent in the Neanderthal and Denisovan branches. In contrast, the low-*ω* traces were more monotonous across many domains, suggesting continuous pressure for broadly tuned, purifying selection. Note that some biclusters encompass more than one domain (Supplementary Figures 3, 4), likely reflecting complex periods of co-evolution across domains.

These trends were reflected in the number of unique genes across the biclusters per branch and FN (Figure 4B). Specifically, we found that the number of unique genes associated with each socio-affective domain was higher for Interaction and Social Concepts, with the values of AMH being particularly prominent in these domains and branch 6 distinguishing itself in the Social Concepts domain (Figure 4B, *left*). Moreover, the low-*ω* results demonstrate that AMH consistently exhibits a significantly higher number of unique genes across all socio-affective domains than other species or branches (Figure 4B, *right panel*). Among the domains, Interaction, Social Concepts, and Introspection consistently showed the highest number of unique genes, whereas Affective Processing exhibited the lowest (Figure 4B, *left and right panels*). Notably, Social Concepts were under both strong adaptive evolution and purifying selection in Branches 4 and 6 and the AMH, indicating the significance of this domain for human evolution during these periods.

Taken together, the most prominent patterns were the peaks for adaptive evolution in Branch 3, Branch 6, and AMH in FNs underlying higher socio-affective traits (Figure 4B, *left*), whereas the low-*ω* genes were much more widely distributed across branches and FNs (Figure 4B, *right*).

### 3.3 Cellular landscape of evolutionary selection in brain networks

**Figure 6:**
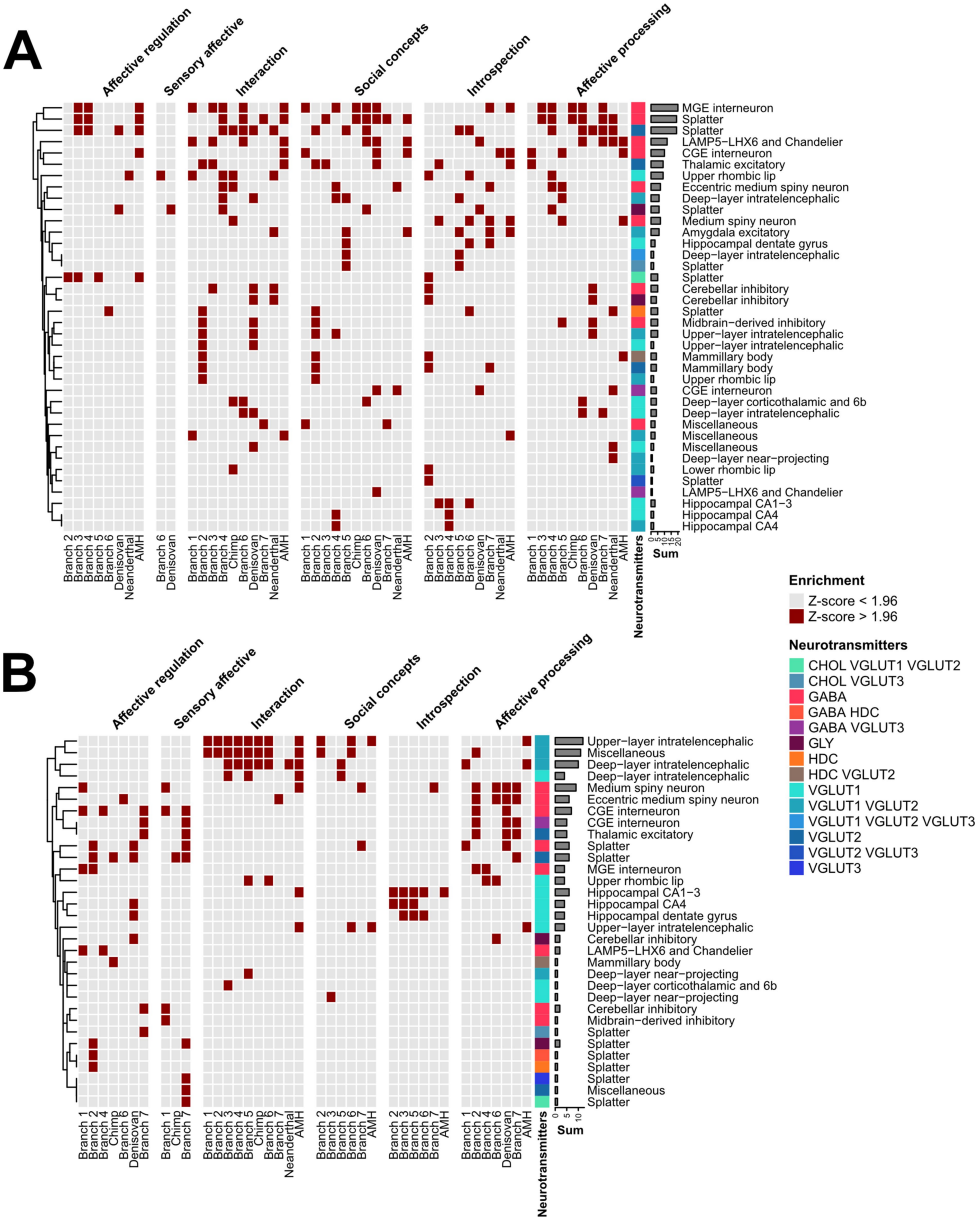
Evolutionary selection across neuronal cells. Phylogenetically ordered enrichment for high ω **(A)** or low ω ranked gene sets **(B)** in neuronal cell types. Gene sets from the top 20 biclusters were correlated with the gene expression of neuronal cell types and binarized column-wise critical z of 1.96 to highlight cell types with the highest correlation in a given branch and socio-affective domain. Counts represent row sums of the respective cell types and highlight evolutionary hotspots across the AMH phylogeny. The respective cell-type auto-annotated neurotransmitter class is highlighted as a color code. Such enrichment can be performed at the bicluster or branch-specific level. In addition, the critical-z can be set up as less stringent to include more cell types.

**Figure 7:**
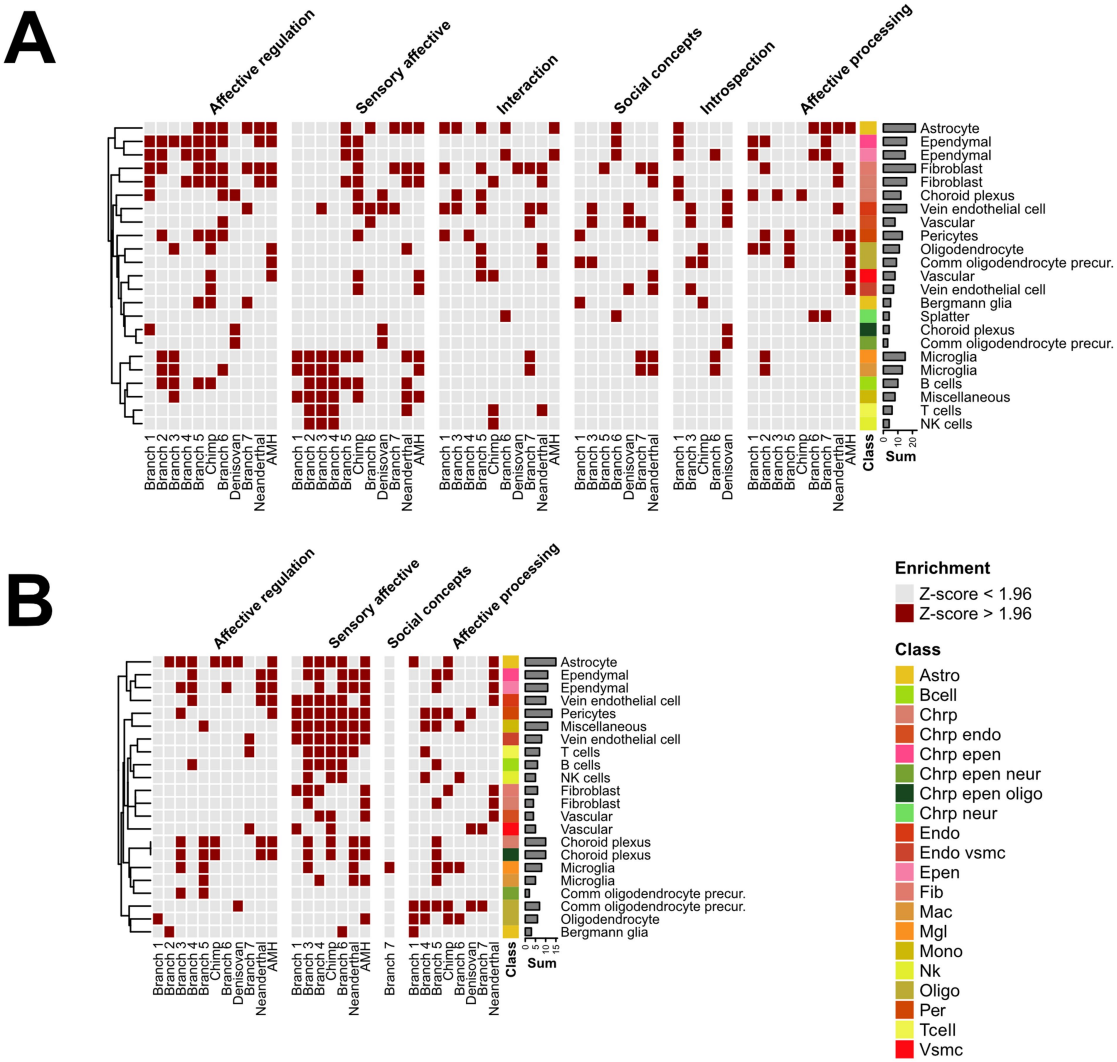
Evolutionary selection across non-neuronal cell types. Phylogenetically ordered enrichment for high ω **(A)** or low ω ranked gene sets **(B)** in non-neuronal cell types. Gene sets from the Top 20 biclusters were correlated with the gene expression of non-neuronal cell types and binarized column-wise at a critical z of 1.96 to highlight cell types with the highest correlation in a given branch and socio-affective domain. Counts represent row sums of the respective cell type and highlight evolutionary hot spots across AMH phylogeny. The respective auto-annotated cell class type is highlighted as a color code. Note that such enrichment analysis can be performed on a bicluster or branch level. Also, the critical-z can be set up as less stringent to include more cell types.

Which cell types drive most of these evolutionary changes? Here, the increasing amount of single-cell data across brain regions becomes the most informative, as it allows us to identify evolutionary hotspots across brain cell types. To identify evolutionary hotspots across brain cell types, we correlated the gene sets contained within each of the biclusters (Supplementary Data 1) with the gene expression profiles of the major brain cell types (Figure 1, Step 5). This should identify the cell types that mediate most of the evolutionary changes associated with each of these clusters for each branch. In our previous approach, we utilized a dataset allowing for 61 cell type/brain region combinations to be mined for such enrichment (Kaczanowska et al., 2022; Lake et al., 2018). Here, we specifically used (Siletti et al., 2023) cell expression data sampled throughout the entire human brain, allowing for a much deeper enrichment analysis across 461 cell clusters, significantly improving the available search space and covering both neuronal and non-neuronal cell types. Overall, the number of biclusters (total matrix count) for neuronal cells was more diverse for high-*ω* (210 matrix counts across 38 cell types) than for low-ω (126 matrix counts across 32 cell types) biclusters, reflecting diverse “exploration” in adaptive evolution and focused purifying selection of fixed traits in the latter group. Specifically, the row counts highlight the strong adaptive evolution of diverse excitatory and inhibitory neurons (Figure 6A, Splatter), whereas purifying selection favors intra-telencephalic projection neurons (Figure 6B, Upper and deep layer intra-telencephalic). For non-neuronal cell types, an overall similar pattern emerged with 247 matrix counts across 23 cell types for high-ω (Figure 7A) and over 167 matrix counts across 22 cell types for low-ω (Figure 7B). Row counts indicated that astrocytes and ependymal cells harbored most of the adaptive evolution (Figure 7A) and purifying selection (Figure 7B) events. Thus, at the cellular level, socio-affective traits, and perhaps brain function in general, seem to evolve through the interplay between adaptive evolution and purifying selection across a broad range of neuronal and non-neuronal cell types.

### 3.4 Molecular features underlying brain evolutionary adaptation

**Figure 8:**
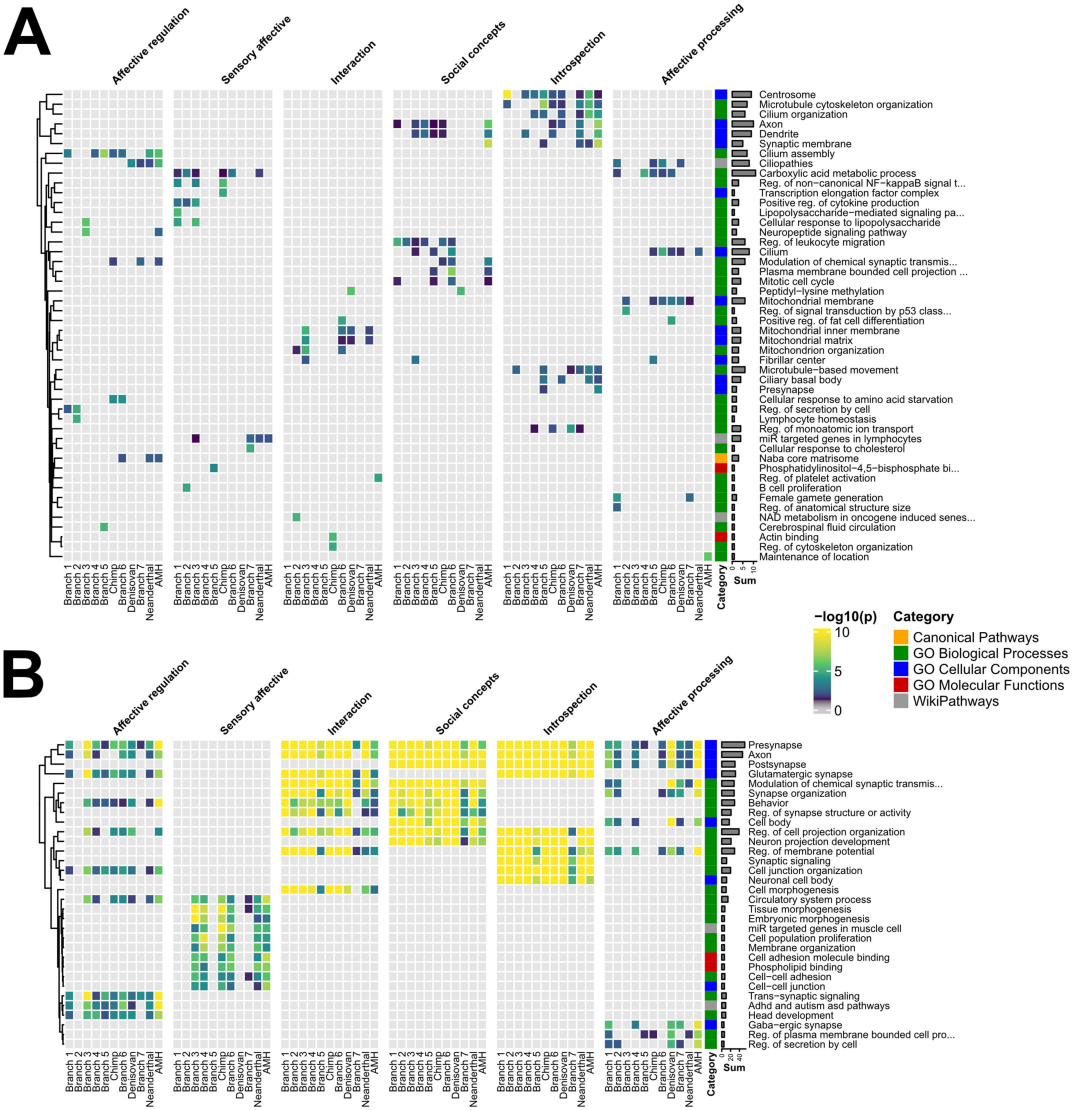
Evolutionary selection across molecular features. Phylogenetically ordered enrichment for high ω **(A)** or low ω ranked gene sets **(B)** for GO terms across different socio-affective domains. Gene sets from the Top 20 biclusters were screened for enrichment using Metascape, with significant hits (*p* < 0.05) for individual GO terms further filtered for the top 10 terms in a given branch and socio-affective domain. Counts represent row sums of the respective GO term and highlight evolutionary hot spots across the AMH phylogeny (i.e., high incidence of a molecular pathway in the biclusters is either positively/relaxed or fixed). The respective GO categories are highlighted in the row color code. Note that such enrichment can be performed at the level of biclusters or branches, as well as for different ω cutoffs.

The obtained bicluster datasets can be also mined for insights into molecular-level mechanisms under evolutionary selection (Figure 1, Step 6). Here, the total matrix count (Figure 8) for high-*ω* bicluster gene sets (177 counts across 48 GO terms) was lower than that for low-ω biclusters (538 counts across 32 GO terms). This likely reflects that purifying selection operates mostly over canonical neuronal pathways, where adaptive evolution spreads more within and between GO term boundaries.

Close inspection at the level of the top GO term counts (Figure 8, *Row sums*) and their respective genes (Supplementary Data 1) further supports this hypothesis. For example, high-ω sets were associated with a broad set of neuro-specific features, such as axonal and dendritic functions (GAP43, MAP6, EXOC6, CXADR, and DBN1) and neuronal receptor signaling (HRH1, HTR1A, HTR2A, and HTR4), to broad features such as signaling (DUSP6, EDN1, and MAPK1), metabolic processes (GGCX, NDUFA9, OXCT1, COQ3, and COQ7), and cell proliferation and brain development (NF2, CX3CL1, SLC16A2, TOX, and SPINT2) (Figure 8A; Supplementary Data 2). This indicates that adaptive selection engages brain expansions to metabolic support via a broad set of mechanisms, in parallel to fine-tuning neuronal communication and brain functions via neuro-specific mechanisms. Conversely, the low-ω set exhibited enrichment in more canonical neuronal processes, such as axonal (GAP43, MAP6, EXOC6, CXADR, and DBN1) and synaptic organization (PSD, CTNND1, BAIAP2, CDKL5, SH3GL3, and FAM81A) and signaling (GRIN1, GABRB3, HTR2A, SNAP25, RAB3A). Thus, the enrichment of genes with low-ω ratios in neural development and synaptic organization likely reflects the evolutionary constraints necessary to maintain core nervous system functions.

Together, these annotations complete a rather complex genetic (ω) to molecular/cellular features (GO) and systems-level (FNs) evolutionary landscape of the social brain. Perhaps most strikingly, it evolved not only through strictly neuronal but also through many non-neuronal cell types and molecular features.

## 4 Discussion

### 4.1 Data science pipelines in computational neuroarcheology

Fueled by brain and genomic initiatives, the wealth of genetic and brain data is increasing dramatically worldwide. Likewise, an ever-expanding set of algorithms is available to mine such data for insights, from partial least square methods (Hansen et al., 2021, 2022) to biclustering techniques (Kaczanowska et al., 2022) and finally to AI tools (Lee et al., 2025; Beebe-Wang et al., 2021). These algorithms facilitate the identification of subgroups or clusters that may not be readily apparent through conventional clustering methods because they concurrently optimize both the selection of pertinent features and the grouping of data points. This renders them invaluable tools in fields such as bioinformatics, where discerning meaningful non-linear patterns is often essential for comprehending underlying processes or making predictions. The current key challenge is to combine the right choice of multimodal datasets with a suitable algorithm into straightforward workflows for novel insights. While genetic datasets and individual algorithms are being generated, the next frontier will be to provide and popularize workflows that integrate multimodal data on one hand and mining algorithms on the other for easy-to-use neuroscientific insight.

To illustrate and address this need, we embedded an early pilot study (Kaczanowska et al., 2022) for low-access exploration of human brain evolution. Setting out, we identified two key limitations of our earlier approach: the need for more comprehensive genetic and FN datasets and improving the user-friendly adaptability of the workflows to other evolutionary or neurogenetic research questions. We addressed this by relaxing three constraints on *ω*-values to expand the genetic dataset for more robust later biclustering (Figures 1, 2). To increase our FN space, we transitioned our workflow to incorporate data from the Neurosynth database, an expanding open database that covers the entire spectrum of human tasks (Figure 1; Supplementary Figure 2). Simultaneously, integrating the Neurosynth database into our workflow allows for easy keyword-based querying and analysis of the selected task sets by the user. Moreover, we embedded this approach into a low-access, user-friendly workflow that reconstructs and links trait patterns at (i) the brain FN, (ii) cellular, and (iii) molecular levels (Figure 1).

### 4.2 Divergent evolutionary pressures on socio-affective traits

By integrating archaic genome data, human brain gene expression, and fMRI network data, this study uncovered evolutionary patterns that connect genetic changes to alterations in the socio-affective brain networks. This study builds upon a previous study (Kaczanowska et al., 2022) to reconstruct traces of adaptive selection across cognitive traits, offering a comprehensive perspective on the evolutionary pressures that shape diverse cognitive functions.

Comparative genomics has identified the key genetic factors underlying human brain evolution. Genes exhibiting accelerated evolution in humans frequently play roles in brain development, as well as neuronal function and cognition (Florio et al., 2015; Pinson et al., 2022; Enard et al., 2009). Furthermore, comparative studies of Neanderthal and Denisovan allelic variants and introgressions provide insights into the genetic basis of human cognitive traits and the potential impact of interbreeding (Zeberg et al., 2024). However, these approaches typically focus on single genes and only a limited number of brain functions (e.g., language, disease, and cultural evolution) (Zhou et al., 2024; Reilly et al., 2022; Enard et al., 2009; Rong et al., 2023; Fogarty et al., 2017). Here, we sought to address the need to complement these target approaches with a more holistic reconstruction of multigenic effects across a broad range of brain functional traits (Piszczek et al., 2023). Therefore, we built our approach on a genome-wide exploration of brain gene sets that have undergone adaptive evolution (high-*ω* ratios) and those that have been under a fixed selection pressure (low-ω ratios) within the human phylogenetic tree. Such evolutionary genetic approaches have been successful in approximating multigenic cognitive evolution (Piszczek et al., 2023). At the brain functional level, we sought to illustrate this approach by reconstructing the evolutionary history across social-affective traits, colloquially thought of as particularly “human” attributes.

At the brain functional level, our workflows identified six major functional groups of socio-affective domains with distinct FN compositions across human socio-affective tasks for simple interpretation (Figure 5; Supplementary Figure 1): Affective Regulation encompasses the neural processes involved in managing and modulating emotional responses (e.g., Facial expressions); Sensory-Affective captures the primary integration of sensory inputs with emotional states (e.g., Pain); Interaction domain groups brain networks for higher social engagement and communication (including for instance, Language); Social Concepts refers to the higher cognitive domains that underpin our understanding of social norms and relationships (e.g., Theory of Mind); Introspection reflects the brain’s capacity for self-reflection and awareness (e.g., Autobiographical); Affective Processing encompasses the neural mechanisms involved in recognizing and interpreting emotional stimuli (e.g., Emotional faces).

To mine these FNs for evolutionary traces, we explored relaxed criteria to increase the power of our approach. This yielded a harmonized ranking of high and low-*ω* values, reflecting adaptive and purifying selection, respectively (Figure 2). Projecting these into brain space by GABi biclustering, we found that, overall, low-ω, fixed biclusters have a rather broad distribution across six domains and branches. In contrast, clusters with high-ω values exhibited greater variability across the domains and branches (Figure 3). This scenario likely reflects a constant drive for purifying selection with interspecies periods of adaptive evolution by specific traits (Figure 4). Interestingly, these spurs of high-ω are particularly pronounced in the Interaction and Social concept domains, traits associated with high social functioning, including Language and Theory of Mind FNs, respectively, peaking in early hominoid, hominin, and particularly AHM evolution. These data provide a deeper understanding of the genetic foundations underlying human socio-affective networks, which influence critical aspects of human behavior. This may suggest that socio-affective functions have evolved in response to unique environmental or social challenges.

When correlating the bicluster gene sets to the cellular level, we found that the pattern was similar to that observed in the FNs, revealing that adaptive evolution engages a broad spectrum of neuronal (Figure 6A) and non-neuronal cell types (Figure 7A), whereas purifying selection accumulates in more specific cellular subsets (Figure 6B; Figure 7B). Interestingly, adaptive evolution in Social concepts and Interaction biclusters involves various cortical and subcortical inhibitory and excitatory cell types (Figure 6A, Interaction, Social concepts). In contrast, purifying selection, for instance, in biclusters for Introspection, precipitates in excitatory hippocampal principal neurons, known for their conserved function in episodic memory (Figure 6B, Introspection).

Next, we investigated the evolutionary pattern at the genetic level by integrating the Metascape results into our workflow. Surprisingly, we found that adaptive evolution shapes a broad class of canonical neuro-specific and non-neuronal features, whereas purifying selection primarily fixes canonical neuro-specific features. In part, adaptive evolution and purifying selection target the same GO features, sometimes alternating and sometimes simultaneously (Figure 8; Axon for Introspection), highlighting their exceptional importance.

For high *ω*-values, the most prominent findings included HTR1A (5-hydroxytryptamine receptor 1A) and HTR2A (5-hydroxytryptamine receptor 2A), which are well-known serotonin receptors. HTR1A acts as an inhibitory receptor in the serotonergic system and plays a significant role in regulating aggressive behavior (Pavlov et al., 2012; Fanselow and Wassum, 2016). HTR1A dysregulation has been linked to disorders such as depression and generalized anxiety disorder (Albert et al., 2011). In contrast, HTR2A is known for its excitatory effects on serotonergic signaling, which has been associated with various mental disorders, including schizophrenia, depression, and mood disorders (Drago and De Ronchi, 2007). However, there is notable enrichment in non-canonical neuronal features, such as pathways associated with metabolic processes, cellular structures, and mitochondrial components. An interesting example is OXCT1 (3-oxoacid CoA-transferase 1), which is involved in ketone body metabolism. This finding highlights the significance of alternative energy pathways, such as ketone metabolism, which is crucial for the utilization of ketone bodies as an energy source, particularly during periods of fasting or low carbohydrate intake when glucose availability is reduced, thereby facilitating adaptation to dietary variability. This process can influence cognitive function and other metabolic disorders, as ketone bodies play a vital role in supporting brain energy (Altayyar et al., 2022).

Conversely, genes with low-*ω* for synapse organization, neuronal cell body functions, and transport vesicle functions. A noteworthy finding is SYP (Synaptophysin), a synaptic vesicle membrane protein that plays a pivotal role in neurotransmitter release, synaptic vesicle trafficking, and synaptic plasticity. Moreover, genetic polymorphisms in the SYP gene have been associated with attention-deficit/hyperactivity disorder (ADHD) (Kim et al., 2023; Liu et al., 2013). Another prime example is GRIN1 (Glutamate Ionotropic Receptor NMDA Type Subunit 1), which is part of the glutamate receptor family. These receptors are complex protein structures composed of multiple subunits that form ligand-gated ion channels. NMDA receptors are essential for synaptic plasticity. Mutations in GRIN1 can result in GRIN1-related neurodevelopmental disorder (GRIN1-NDD), which manifest as a wide range of developmental and intellectual challenges. Affected individuals typically experience developmental delay or intellectual disability, along with other common symptoms, such as epilepsy, muscle weakness, movement disorders, spasticity, feeding difficulties, and various behavioral issues (Platzer and Lemke, 1993). Ultimately, these functions might have held this gene evolutionarily in a fixed state.

### 4.3 Conclusions on the proposed workflow

Together, our proposed workflow (Figure 1) illustrates the potential of utilizing synthetic databases and genetic evolution data to elucidate the evolutionary dynamics that shape the human socio-affective networks. By identifying specific genetic sets, this methodology offers a deeper understanding of how genetic changes contribute to the development and maintenance of socio-affective traits essential for social behavior, emotional regulation, and complex interpersonal interactions. These findings provide significant insights into the genetic foundations underlying human socio-affective networks, highlighting the intricate balance between adaptive evolution and the conservation of core biological functions. While certain genes evolve to enhance specific competencies crucial for human interaction, others are preserved to maintain stability in the essential neural processes. This balance underscores the complexity of the genetic architecture that governs both human social behavior and the foundational mechanisms of neural function. Ultimately, we encourage comprehensive workflows that trace evolutionary forces across multiple biological levels for a holistic molecular-to-systems-level exploration of human (socio-affective) evolution. This may complement focused mechanistic studies in evolutionary genetics and comparative neuroscience (e.g., with organoid models) (Piszczek et al., 2023).

Of course, the workflow presented here (Figure 1) makes two hopeful approximations. First, it relies on *ω*-values as proxies for selection pressure. However, other genomic features certainly also contribute to evolutionary adaptation, evolution of non-coding DNA (long non-coding RNA, enhancers), specific genes (human-specific genes), and Human Accelerator Regions, which might contribute to cognitive evolution and should be considered mining in a similar manner. Likewise, current workflows mine for traces of ancient evolution in extant AMH FNs. Interpolating ancestral brain features along human phylogeny and then mining for adaptive evolution or purifying selection in these (imputed) ancestral frameworks (e.g., expression profiles, FNs) would certainly yield additional complementary insights. Indeed, with increasingly sophisticated methods of interpolation of such ancestral functional neuroanatomical maps (Schwartz et al., 2023), this is now possible. Including this information into our workflows (e.g., projecting ancestral gene expression patterns onto these ancestral neuroanatomical maps) would be the next logical step.

### 4.4 Future perspectives on computational neuroarcheology

The findings and methodologies presented in this study offer several promising avenues for future research. A primary objective of computational neuroarcheology is to refine techniques for reconstructing the characteristics and functions of the brains of ancestral species. This endeavor involves the application of advanced computational tools, including machine learning, evolutionary modeling, and comparative neuroanatomy to elucidate the cognitive abilities, neural connectivity, and behavioral capacities of extinct species. Several components of the workflow could be adapted to facilitate novel insights: For example, the computation of the correlation between gene expression and FNs could be adapted, as proposed by (Komorowski et al., 2022), to obtain a more informative metric for the importance of a gene for an FN. Alternatively, tools such as Neuromaps could be used for more robust testing of gene expression to FN signal correlations using spin tests via the creation of “null” maps. Moreover, instead of ranking ω and the correlation matrix, one could convert them to probabilities using the soft-max function. Another idea is to focus on genes that are specific to the activated regions of the FNs, in contrast to the remaining brain, for example, computed by differential gene expression analysis. The reduced search space can aid in the identification of relevant genes. By integrating fossil evidence, brain endocast analyses, and neuroimaging data from extant species, researchers can develop high-resolution models of ancestral neural structures (Melchionna et al., 2025; Schwartz et al., 2023; Kochiyama et al., 2018). Furthermore, these reconstructions may facilitate interdisciplinary exploration in environmental, cultural, and ecological contexts, thereby providing deeper insights into the evolutionary pressures that have shaped brain development and functionality over time.

Finally, the workflow is easily adaptable to other genetic and FN database input types. For instance, it can be applied to psychiatric genetics using disease trait-related GWAS weighted genetic data (instead of ω-weighted genetic data) and clinical imaging datasets as inputs. Moreover, this strategy can be adopted for other phylogenies with existing reference brains and evolutionary genetics across the animal kingdom in similar ways.

## Data Availability

The original contributions presented in the study are included in the article/Supplementary material, further inquiries can be directed to the corresponding author.

## Footnotes

1
https://www.nitrc.org/projects/fcon_1000
