# Supplementary figures and images for: Computational reconstruction of evolutionary selection in human brain networks
Source: Front Neuroinform. 2026 Jan 26;19:1623174. doi: 10.3389/fninf.2025.1623174 (PMC12883834; doi:10.3389/fninf.2025.1623174)

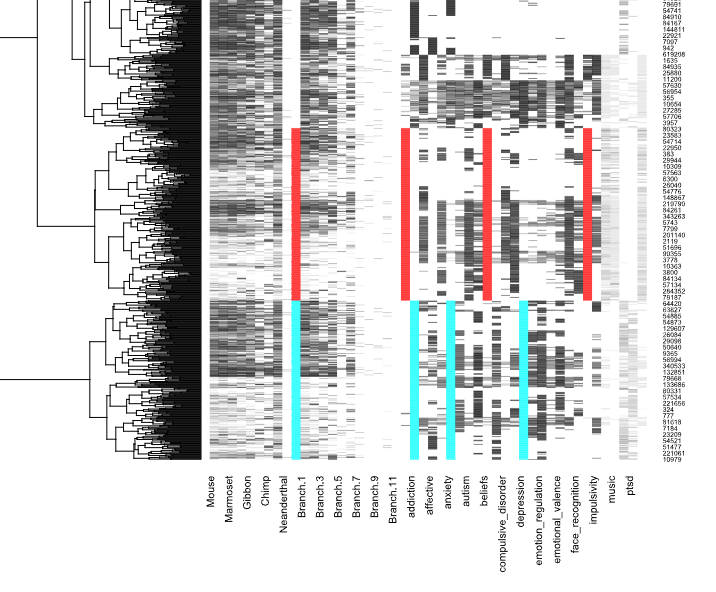

Supplement: Supplementary file 1 [file Data_Sheet_1.zip › Piszczek et al Genetic-Trait-Mining/snippet_clustering.PNG]

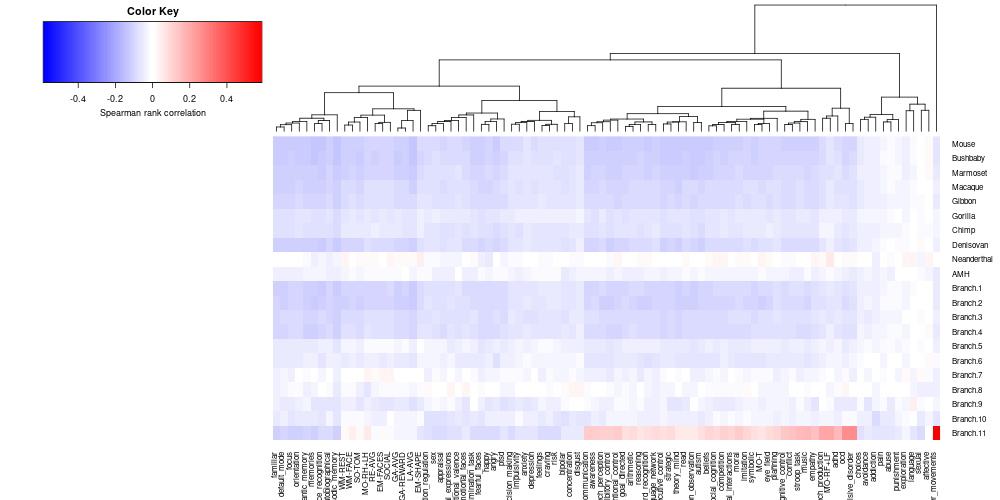

Supplement: Supplementary file 1 [file Data_Sheet_1.zip › Piszczek et al Genetic-Trait-Mining/storage/biclustering/DNDS_col_Network_col_cor.png]

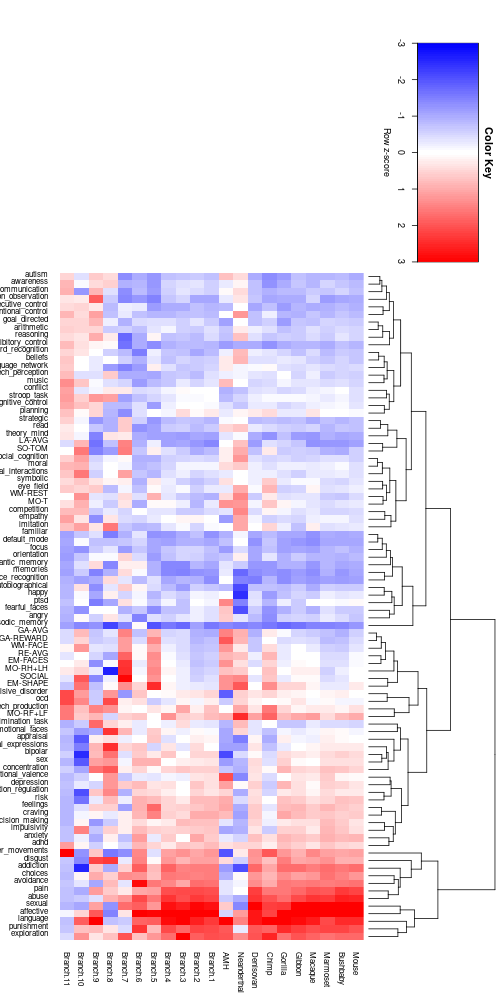

Supplement: Supplementary file 1 [file Data_Sheet_1.zip › Piszczek et al Genetic-Trait-Mining/storage/biclustering/DNDS_col_Network_col_cor_row_norm.png]

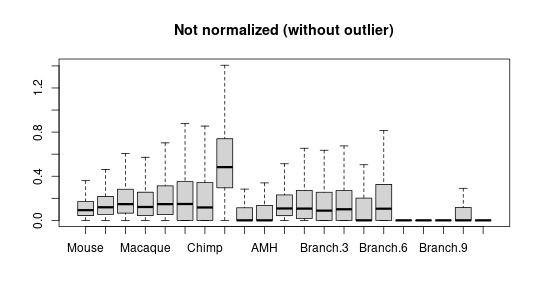

Supplement: Supplementary file 1 [file Data_Sheet_1.zip › Piszczek et al Genetic-Trait-Mining/storage/dnds_distribution_plots/dnds_boxplot.png]

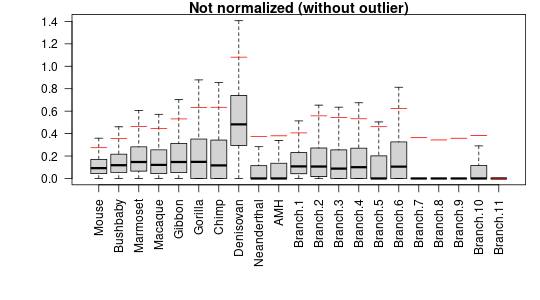

Supplement: Supplementary file 1 [file Data_Sheet_1.zip › Piszczek et al Genetic-Trait-Mining/storage/dnds_distribution_plots/dnds_boxplot_90percentile.png]

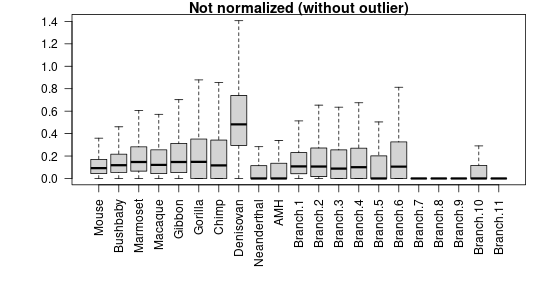

Supplement: Supplementary file 1 [file Data_Sheet_1.zip › Piszczek et al Genetic-Trait-Mining/storage/dnds_distribution_plots/dnds_boxplot_quantiles.png]

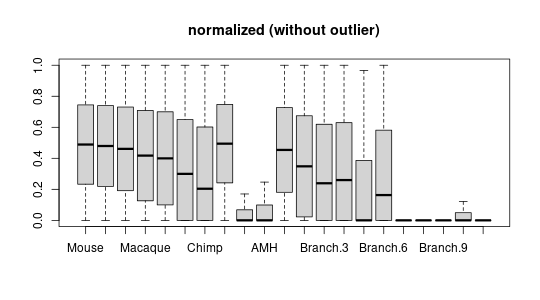

Supplement: Supplementary file 1 [file Data_Sheet_1.zip › Piszczek et al Genetic-Trait-Mining/storage/dnds_distribution_plots/normalized_dnds_boxplot_without_outlier.png]

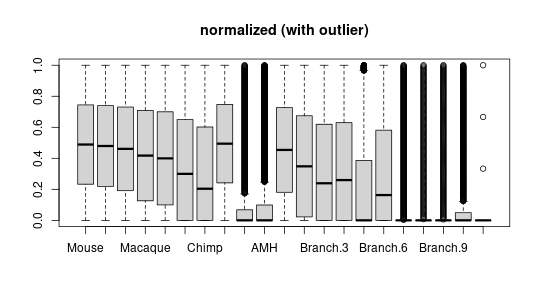

Supplement: Supplementary file 1 [file Data_Sheet_1.zip › Piszczek et al Genetic-Trait-Mining/storage/dnds_distribution_plots/normalized_dnds_boxplot_with_outlier.png]
