# Supplementary material for: Computational reconstruction of evolutionary selection in human brain networks
Source: Front Neuroinform. 2026 Jan 26;19:1623174. doi: 10.3389/fninf.2025.1623174 (PMC12883834; doi:10.3389/fninf.2025.1623174)
Supplement: Supplementary file 2 [file Supplementary_file_1.docx]

**Supplementary Material**

**
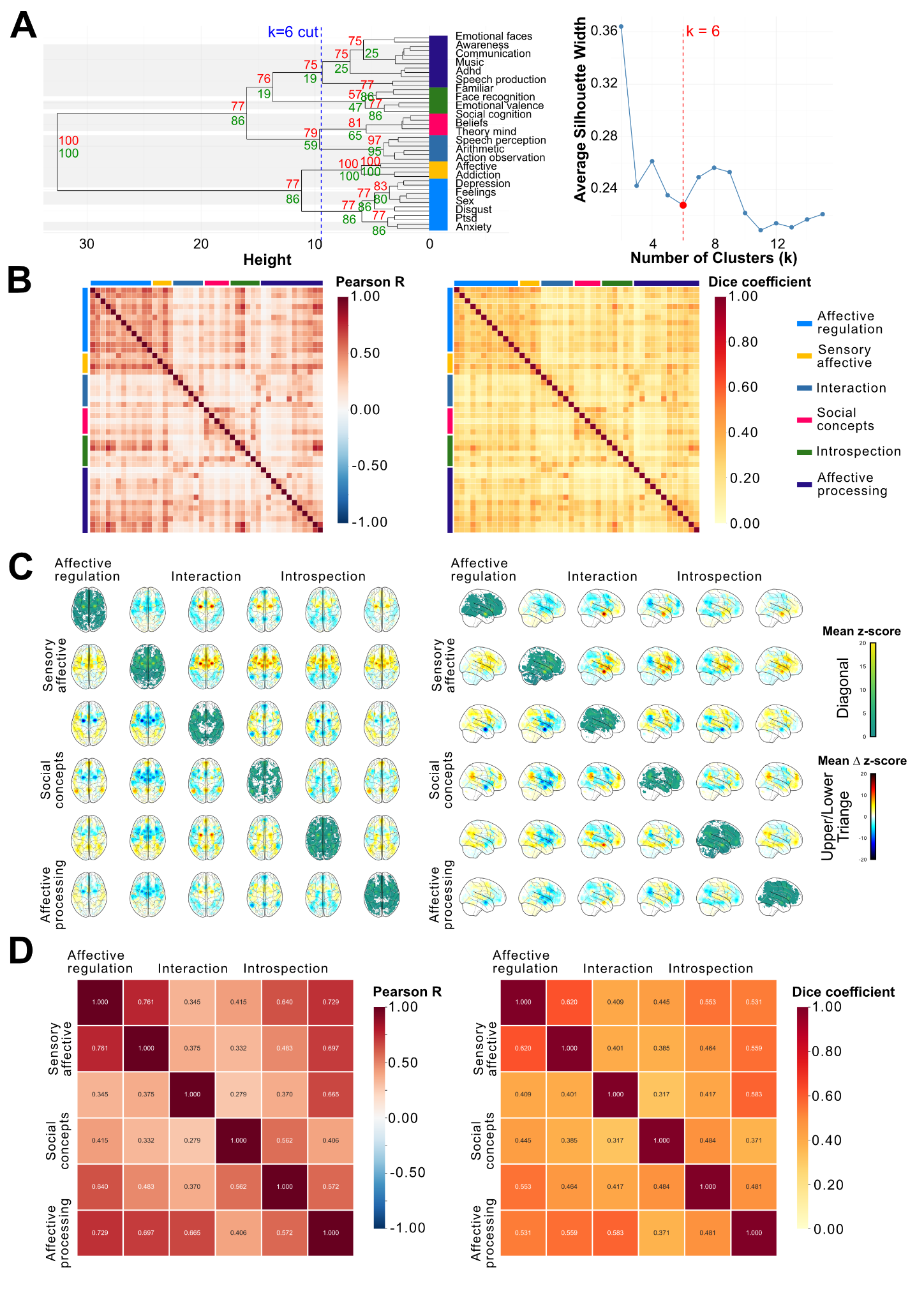
**

**Supplementary Figure 1. Classification of Neurosynth terms and domains similarity patterns**

**(A)** *Left:* **Evaluation of cluster stability** in the hierarchical clustering analysis of Neurosynth socio-affective FNs and ABA gene expression correlations across biopsy sites, organized into six socio-affective domains. The AU (Approximately Unbiased) p-value (%) is indicated in red, the BP (Bootstrap Probability) value (%) in green, and the height of the dendrogram cut for the chosen number of clusters is shown in blue. Clusters with an AU greater than 95% (thus, strongly supported by the data) are highlighted in gray. For text clarity, every second term is shown. *Right*, **determining the optimal number of clusters** using the Silhouette average width method across various cluster numbers. Here, k = 6 was selected.

**(B)** Pearson regression coefficient (*left*) and Dice coefficient (*right*) showing similarities in the patterns between the individual terms from Nerosynth.

**(C)** Axial (*left*) and sagittal (*right*) views of the average z-score task-fMRI signal within individual socio-affective domains (across diagonal) and the differential z-score task-fMRI signal between the domains (upper and lower triangles), calculated and plotted using nilearn.

**(D)** Pearson regression coefficient (*left*) and Dice coefficient (*right*) showing the similarities of the patterns between the socio-affective domains.


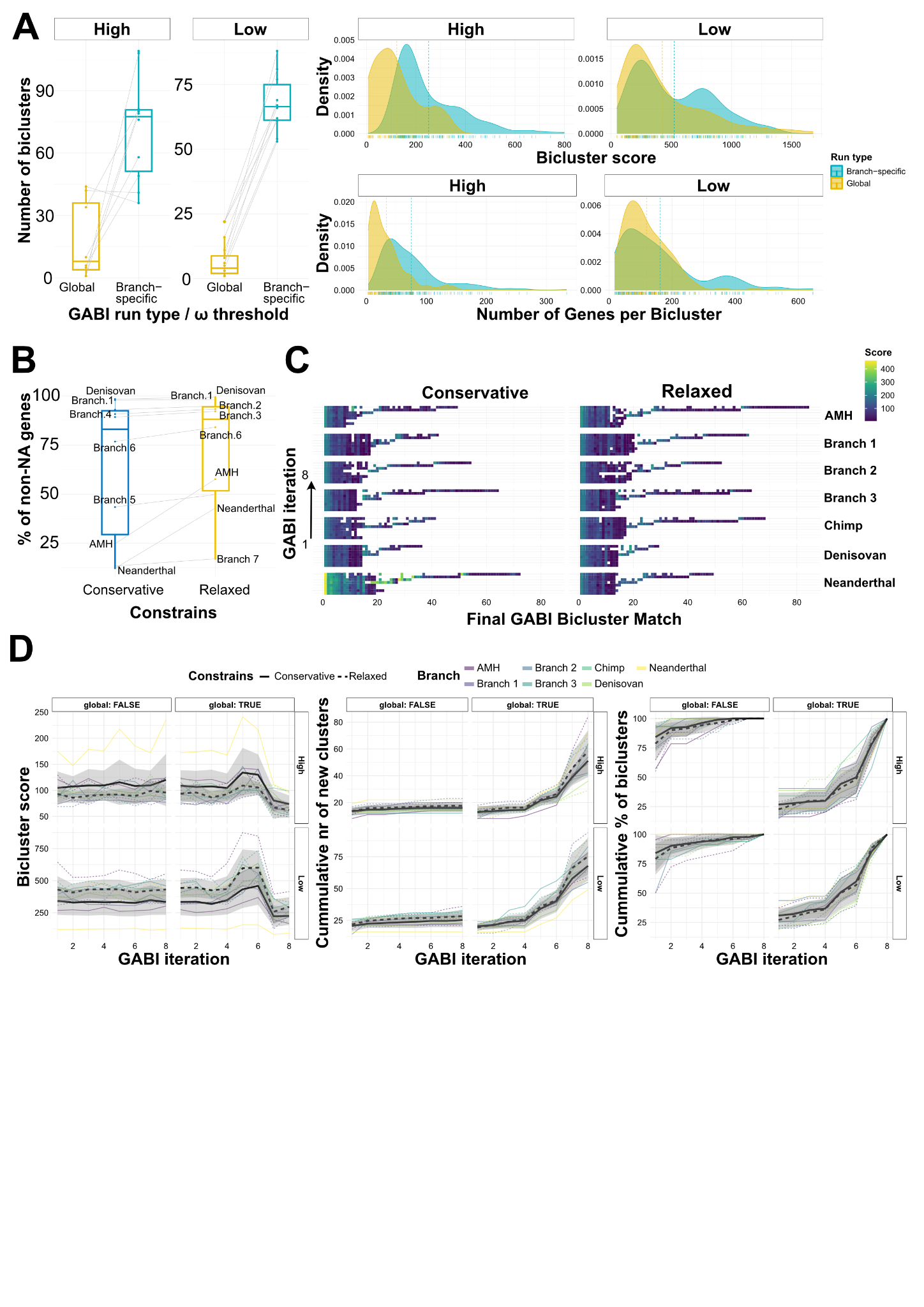


**Supplementary Figure 2. Computational workflow parameter space.**

**(A)** Impact of fixed (global) vs branch-specific ω thresholding using conservative constraints on selected Branches showing (*left*) number of detected GABI biclusters overall and (*right*) the distribution of bicluster scores and number of genes per bicluster in the top 20 biclusters per branch. **(B)** Increase in the number of genes that can be used for bicluster subspace mining upon constraint relaxation in selected Branches. (**C**) Heatmap showing the detection of new biclusters (each cell is a heatmap) with consecutive bicluster iterations in the selected branches of the high-ω gene sets. (**D**) Influence of the global optimization (“global_optim”) option on bicluster scores of detected biclusters (*left*), number (*middle*), and percent (*right*) of detected biclusters in selected branches in both high and low ω gene sets.


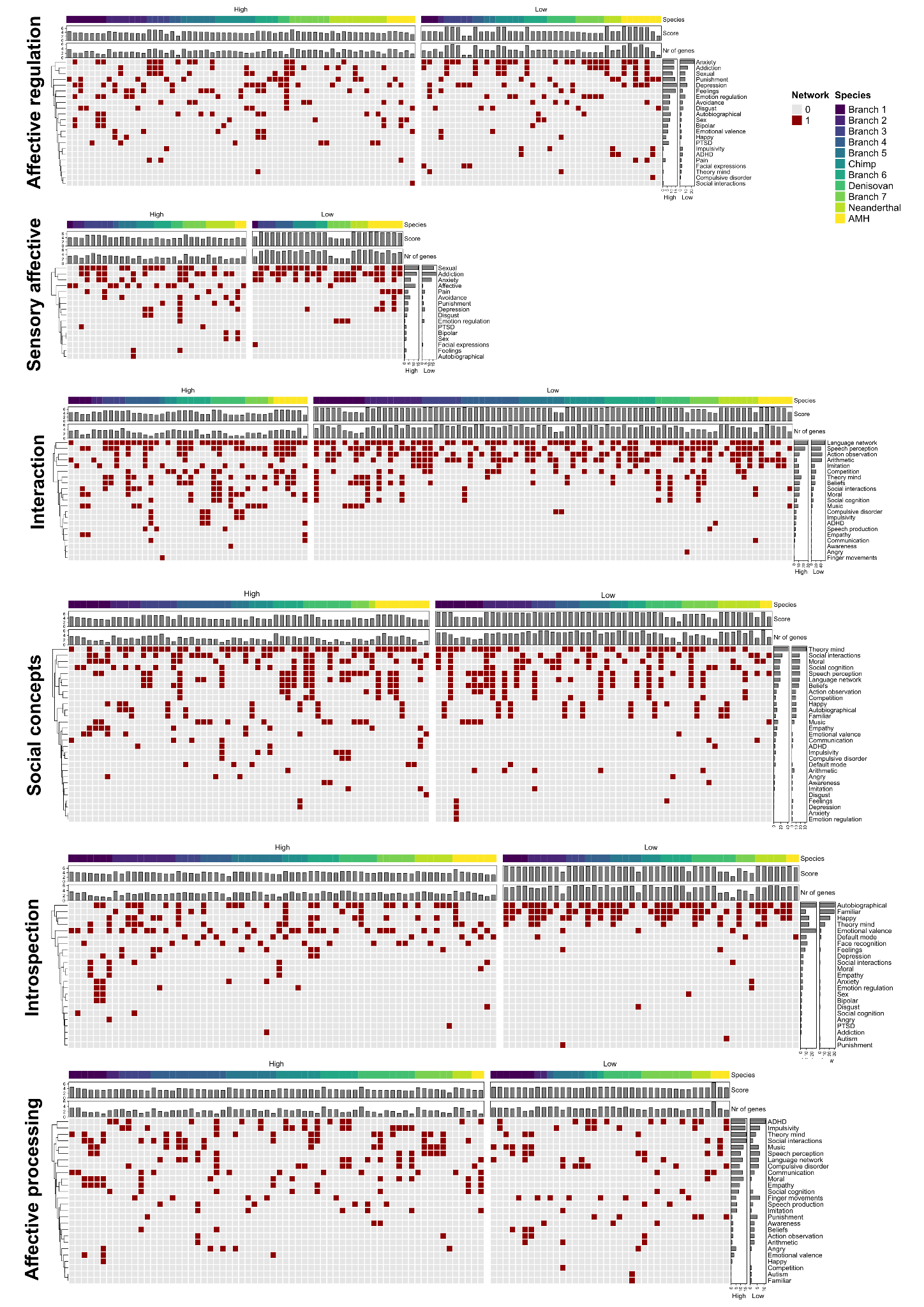


**Supplementary Figure 3. Composition of the biclusters.**

Phylogenetically ordered top 20 biclusters for each branch, for high and low ω brain genes, split by socio-affective domains. The colors above the graphs indicate the branch for each bicluster, while the rows show the Neurosynth fMRI terms. Column counts show the GABi score and number of genes for each bicluster (in log scale), while row counts sum the usage of each Neurosynth term for all biclusters for either high or low ω ranked genes.

**
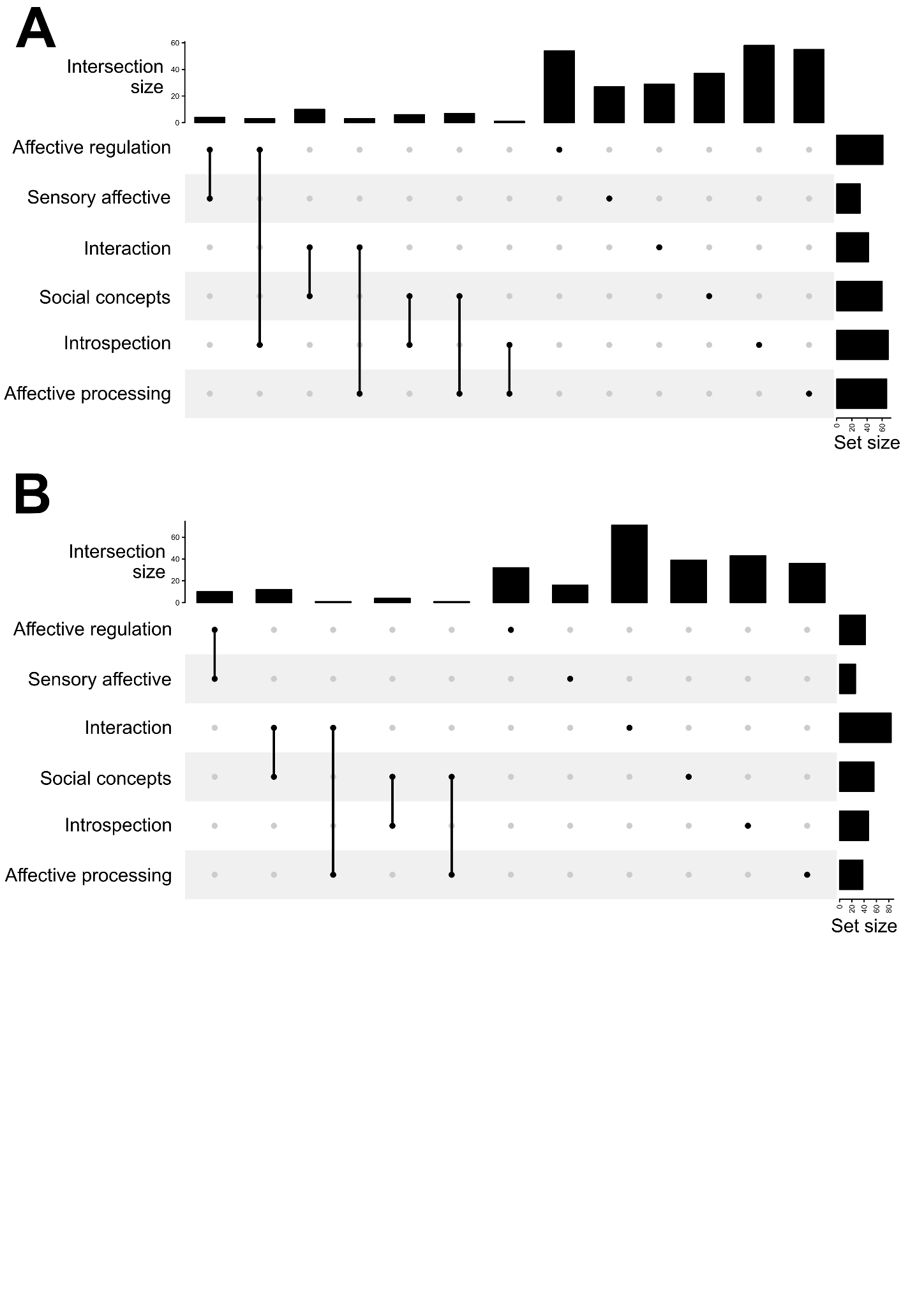
**

**Supplementary Figure 4. Overlap of FNs in the socio-affective domains in the biclusters.**

UpSet plot showing the number of unique and shared socio-affective domains across the top 20 biclusters per species for high (**A**) or low (**B**) ω genes.

**Supplementary Table 1: Socio-affective terms used in the Neurosynth search.**

| **Term** | **Description** |
| --- | --- |
| Action observation | The perception, encoding, and recognition of movements performed by others. |
| Addiction | A compulsive need for a substance or behavior, often pursued despite harmful consequences. Studies on addiction cover a range of topics, including substance abuse, gambling, drug and alcohol dependence, internet gaming, dopamine dependence, nicotine, sex, and internet pornography. |
| ADHD | A neurodevelopmental condition characterized by difficulties in attention, impulse control, and hyperactivity. The studies included in the analyses encompassed all age groups. |
| Affective | It is used to describe emotional responses or experiences. |
| Angry | Complex emotional state that activates specific brain networks associated with threat detection, social evaluation, and emotional memory. |
| Anxiety | These studies explored how anxiety impacts brain activity, showing that individual differences in traits and social anxiety affect neural responses to threat, attention, and emotion processing. |
| Appraisal | It refers to the brain’s process of evaluating and interpreting stimuli (such as emotions, actions, stressors, or personal memories) to inform responses and judgment. |
| Arithmetic | Processing functions, such as calculation, fact retrieval, and number representation, vary depending on developmental stage, mathematical competence, and specific syndromes. |
| Autism | Refers to studies that have highlighted atypical brain activation and connectivity in areas involved in visuospatial processing, emotion and face recognition, working memory, and motor function. |
| Autobiographical | Refers to personal memories and narratives about an individual's own experiences, characterized by emotional significance and social context, involving the retrieval of specific details from past events that influence self-identity and personal storytelling. |
| Avoidance | The behaviors and cognitive strategies that individuals use to evade unpleasant stimuli or situations, often in response to fear or anxiety, involve neural mechanisms, including brain regions related to fear processing and decision-making. |
| Awareness | Conscious recognition and understanding of internal states, external stimuli, or actions encompass emotions, perception, and self-awareness. |
| Beliefs | Mental representations or convictions individuals hold about the world, including interpretations of others' thoughts and emotions, moral judgments, and perceptions of reality, influence behavior and decision-making and are shaped by personal experiences and social interactions. |
| Bipolar | Mental health conditions are characterized by significant mood fluctuations, including episodes of mania and depression, which impact daily functioning and the quality of life. Studies have explored neurobiological differences in brain structure, connectivity, and activation patterns. |
| Communication | The process of conveying information, emotions, and intentions through various modalities, including facial expressions, spoken language, and non-verbal cues, with studies exploring the neural mechanisms involved in producing and interpreting these signals and the cognitive and emotional components essential for effective interaction. |
| Competition | Cognitive processes are involved in resolving conflicting information or responses during tasks that require decision-making, language processing, or memory retrieval. |
| Compulsive disorder | Obsessive-Compulsive Disorder (OCD) is a mental health condition characterized by persistent intrusive thoughts (obsessions) and repetitive behaviors or mental acts (compulsions). Studies have investigated the neurobiological underpinnings, altered brain function, and activation patterns associated with this disorder compared to those of healthy controls. |
| Default mode | The default mode network (DMN), a set of brain regions that are more active when an individual is at rest and not focused on external tasks, is typically involved in self-referential thoughts and internal reflection. Studies have examined its connectivity and functioning under various conditions to understand its role in cognitive processes and clinical implications. |
| Depression | It is a mental health disorder characterized by persistent sadness and loss of interest in activities, with studies exploring its neural correlates, the effects of childhood maltreatment, and its relationships with anxiety, attachment, and suicidal ideation. |
| Disgust | An emotional response of revulsion towards offensive stimuli, including contamination and moral violations. This study explored how individual differences in disgust sensitivity influence neural activation and how experiences such as counterconditioning can modify this emotional response. |
| Emotion regulation | Neural mechanisms of emotion regulation, exploring brain regions involved in emotional processing and regulation (e.g., prefrontal cortex, amygdala), and how individual differences (e.g., sex, mental health, childhood adversity) affect these processes. It addresses both healthy and clinical populations and investigates the role of emotion regulation in conditions such as depression, phobias, and schizophrenia. |
| Emotional faces | Refers to how the brain processes emotional faces, exploring how factors such as childhood experiences, mental health, genetics, age, and medication influence the brain's response to emotional expressions, impacting recognition and emotional processing. |
| Emotional valence | How the brain processes emotional valence (positive and negative emotions), highlighting brain regions such as the amygdala, subcortex, and cortical structures. The papers referring to this term explore how emotional responses are affected by factors such as cross-modal integration, attitudes, memory, and psychological conditions (e.g., depression and Parkinson's disease). |
| Empathy | Neural mechanisms of empathy examine brain regions such as the amygdala and interoceptive areas. These studies explore how empathy is influenced by social signals, mental health conditions (e.g., schizophrenia, PTSD, alexithymia), and factors such as learning, in-group/out-group dynamics, and self-awareness. |
| Face recognition | Neural mechanisms of face recognition, particularly the role of regions such as the fusiform face area and hippocampus. They explore face processing in clinical populations (e.g., prosopagnosia, Alzheimer’s disease, andschizophrenia), the hierarchical network for face recognition, and the distinction between conscious and unconscious face processing. |
| Facial expressions | The neural basis of perceiving and processing emotional facial expressions, with a focus on functional connectivity, emotional learning, and social behavior, often has clinical implications. |
| Familiar | The brain recognizes and processes familiar faces and objects, highlighting the roles of areas such as the fusiform face area and temporo-parietal junction. The focus is on the influence of familiarity on perception, memory, and social interactions using techniques such as fMRI and ERP. |
| Feelings | The brain’s processing, regulation, and integration of emotional states across various contexts, such as social interactions, sensory experiences, and cognitive tasks, with an emphasis on the roles of key regions, such as the anterior insula and frontal-parietal networks, provides insights into the complexity of emotional and interoceptive processes. |
| Finger movements | The brain’s coordination and control of finger movements across various contexts, such as bimanual tasks, sensorimotor transformations, and motor learning, with an emphasis on key regions such as the supplementary motor area and premotor cortex, provide insights into the neural mechanisms underlying motor function and recovery. |
| Happy | The brain's response to happy faces, music, and emotion regulation. The term explores factors such as gender differences, individual variations, and conditions such as PTSD and depression. |
| Imitation | Refers to automatic and controlled imitation, the influence of top-down and bottom-up cues, and the roles of empathy and communication, including the effects of brain damage on imitative behavior. |
| Impulsivity | A tendency to act without adequate forethought, involving challenges in self-control, decision-making, and behavioral regulation. Impulsivity is a multifaceted trait encompassing subtypes such as trait impulsivity, proactive/reactive control, or context-specific impulsive behaviors, often linked to neural activity in regions involved in reward processing, decision-making, and inhibitory control. |
| Language network | The term encompasses functions such as language lateralization, phonological memory, and reading development, studied in contexts such as aging, learning, and neuropsychiatric conditions. |
| Moral | The cognitive and emotional processes behind ethical judgments, decision-making, and responses to moral dilemmas involve regions such as the prefrontal cortex and cortical midline structures and address aspects such as harm, justice, care, and virtue. |
| Music | Refers to auditory stimuli and experiences involving rhythm, melody, harmony and emotional expression. It encompasses processes such as aesthetic appreciation, rhythmic entrainment, emotional responses, and cultural influences on perception and memory. Music has been studied as a stimulus for neural activity in areas related to emotion, motor control, memory, and even language, highlighting its cognitive and emotional impact across diverse contexts. |
| Pain | Refers to both physical and emotional discomfort, focusing on how the brain processes pain in regions such as the anterior insula and orbitofrontal cortex, and how factors such as meditation or cognitive demand affect pain perception. |
| PTSD | Post-Traumatic Stress Disorder is a condition caused by trauma, characterized by altered brain function and structure, particularly in areas such as the insula, amygdala, and cingulate cortex. These studies examined changes in brain connectivity and structure in response to trauma and related symptoms. |
| Punishment | Refers to the neural and behavioral responses to negative consequences aimed at enforcing rules, deterring bad behavior, or promoting norm compliance. These studies have focused on brain areas such as the orbitofrontal cortex and amygdala, which are involved in processing different forms of punishment, including social, monetary, and third-party punishment. |
| Sex | Biological differences between males and females, including the effects of sex chromosomes and hormones on brain structure, function, and behavior (e.g., spatial memory and emotional processing). |
| Sexual | The term focuses on sexual behavior, desire, and responses to sexual stimuli. These studies examine how the brain processes sexual arousal, sexual orientation, and responses to erotic or sexual cues, often in the context of behaviors, emotional responses, or disorders related to sexuality (e.g., sexual desire, risky sexual behavior, and hypersexuality). |
| Social cognition | Brain processes are involved in understanding and responding to social information, such as emotion recognition, social decision-making, and social cues. These studies focus on how these processes are affected by conditions such as schizophrenia, anorexia nervosa, and borderline personality disorder, as well as training interventions. |
| Social interactions | Perceiving, evaluating, and responding to interpersonal behaviors and social cues such as trust, emotion recognition, and decision-making. These studies explore these processes in various contexts and conditions, such as dementia and antisocial behavior. |
| Speech perception | The brain processes spoken language by performing tasks such as decoding sounds, recognizing words, and integrating auditory and motor information. These studies explore the role of areas such as the cerebellum, premotor cortex, Broca's area, and prefrontal cortex in speech perception, considering factors such as aging, individual variation, and sensory influences. |
| Speech production | The neural processes that enable the production of spoken language, particularly focusing on motor areas such as the motor cortex and supplementary motor area. These studies investigated how brain regions, aging, and conditions such as stuttering affect speech articulation and phonation. |
| Theory of mind | The capacity to recognize and attribute mental states, such as beliefs and intentions, to oneself and others. These studies investigated the role of brain regions, such as the superior temporal sulcus and temporo-parietal junction, and explored how impairments in this ability manifest in conditions such as schizophrenia and progressive supranuclear palsy. They also examined how the theory of mind is involved in social interactions and understanding actions. |
