# Supplementary material for: Computational reconstruction of evolutionary selection in human brain networks
Source: Front Neuroinform. 2026 Jan 26;19:1623174. doi: 10.3389/fninf.2025.1623174 (PMC12883834; doi:10.3389/fninf.2025.1623174)
Supplement: Supplementary file 1 [file Data_Sheet_1.zip › Piszczek et al Genetic-Trait-Mining/R_package_sources/biomaRt_2.54.1.tar.gz!/biomaRt/inst/doc/accessing_ensembl.html]

Accessing Ensembl annotation with biomaRt


# Accessing Ensembl annotation with biomaRt

Mike L. Smith, Steffen Durinck, Wolfgang Huber

#### 20 March 2023

#### Package

biomaRt 2.54.1

# Contents

- 1 Introduction
- 2 Selecting an Ensembl BioMart database and dataset
  - 2.1 Step1: Identifying the database you need
  - 2.2 Step 2: Choosing a dataset
  - 2.3 Ensembl mirror sites
  - 2.4 Using archived versions of Ensembl
  - 2.5 Using Ensembl Genomes
- 3 How to build a biomaRt query
  - 3.1 Searching for filters and attributes
  - 3.2 Using predefined filter values
  - 3.3 Finding out more information on filters
    - 3.3.1 filterType
  - 3.4 Attribute Pages
  - 3.5 Using `select()`
- 4 Result Caching
- 5 biomaRt helper functions
  - 5.1 exportFASTA
- 6 Examples of biomaRt queries
  - 6.1 Annotate a set of Affymetrix identifiers with HUGO symbol and chromosomal locations of corresponding genes
  - 6.2 Annotate a set of EntrezGene identifiers with GO annotation
  - 6.3 Retrieve all HUGO gene symbols of genes that are located on chromosomes 17,20 or Y, and are associated with specific GO terms
  - 6.4 Annotate set of idenfiers with INTERPRO protein domain identifiers
  - 6.5 Select all Affymetrix identifiers on the hgu133plus2 chip and Ensembl gene identifiers for genes located on chromosome 16 between basepair 1100000 and 1250000.
  - 6.6 Retrieve all EntrezGene identifiers and HUGO gene symbols of genes which have a “MAP kinase activity” GO term associated with it.
  - 6.7 Given a set of EntrezGene identifiers, retrieve 100bp upstream promoter sequences
  - 6.8 Retrieve all 5’ UTR sequences of all genes that are located on chromosome 3 between the positions 185,514,033 and 185,535,839
  - 6.9 Retrieve protein sequences for a given list of EntrezGene identifiers
  - 6.10 Retrieve known SNPs located on the human chromosome 8 between positions 148350 and 148420
  - 6.11 Given the human gene TP53, retrieve the human chromosomal location of this gene and also retrieve the chromosomal location and RefSeq id of its homolog in mouse.
- 7 Connection troubleshooting
  - 7.1 *biomaRt* specific solutions
  - 7.2 Global connection settings
    - 7.2.1 Error: “SSL certificate problem”
    - 7.2.2 Error: “sslv3 alert handshake failure”
- 8 Session Info

# 1 Introduction

Accessing the data available in Ensembl is by far most frequent use of the *biomaRt* package. With that in mind *biomaRt* provides a number of functions that are tailored to work specifically with the BioMart instances provided by Ensembl. This vignette details this Ensembl specific functionality and provides a number of example usecases that can be used as the basis for specifying your own queries.

# 2 Selecting an Ensembl BioMart database and dataset

Every analysis with *biomaRt* starts with selecting a BioMart database to use. The commands below will connect us to Ensembl’s most recent version of the Human Genes BioMart.

```
library(biomaRt)
ensembl <- useEnsembl(biomart = "genes", dataset = "hsapiens_gene_ensembl")
```

If this your first time using *biomaRt* , you might wonder how to find the two arguments we supplied to the `useEnsembl()` command. This is a two step process, but once you know the setting you need you can use the version shown above as a single command. These initial steps are outlined below.

## 2.1 Step1: Identifying the database you need

The first step is to find the names of the BioMart services Ensembl is currently providing. We can do this using the function `listEnsembl()`, which will display all available Ensembl BioMart web services. The first column gives us the name we should provide to the `biomart` argument in `useEnsembl()`, and the second gives a more comprehensive title for the dataset along with the Ensembl version.

```
listEnsembl()
```

```
##         biomart                version
## 1         genes      Ensembl Genes 109
## 2 mouse_strains      Mouse strains 109
## 3          snps  Ensembl Variation 109
## 4    regulation Ensembl Regulation 109
```

The `useEnsembl()` function can now be used to connect to the desired BioMart database. The `biomart` argument should be given a valid name from the output of `listEnsembl()`. In the next example we will select the main Ensembl mart, which provides access to gene annotation information.

```
ensembl <- useEnsembl(biomart = "genes")
```

If we print the current `ensembl` object, we can see that the ENSEMBL\_MART\_ENSEMBL database 111 this is how Ensembl name the database on their server has been selected, but that no dataset has been chosen.

```
ensembl
```

```
## Object of class 'Mart':
##   Using the ENSEMBL_MART_ENSEMBL BioMart database
##   No dataset selected.
```

## 2.2 Step 2: Choosing a dataset

BioMart databases can contain several datasets. For example, within the Ensembl genes mart every species is a different dataset. In the next step we look at which datasets are available in the selected BioMart by using the function `listDatasets()`. *Note: here we use the function `head()` to display only the first 5 entries as the complete list has 213 entries.*

```
datasets <- listDatasets(ensembl)
head(datasets)
```

```
##                        dataset                           description     version
## 1 abrachyrhynchus_gene_ensembl Pink-footed goose genes (ASM259213v1) ASM259213v1
## 2     acalliptera_gene_ensembl      Eastern happy genes (fAstCal1.2)  fAstCal1.2
## 3   acarolinensis_gene_ensembl       Green anole genes (AnoCar2.0v2) AnoCar2.0v2
## 4    acchrysaetos_gene_ensembl       Golden eagle genes (bAquChr1.2)  bAquChr1.2
## 5    acitrinellus_gene_ensembl        Midas cichlid genes (Midas_v5)    Midas_v5
## 6    amelanoleuca_gene_ensembl       Giant panda genes (ASM200744v2) ASM200744v2
```

The `listDatasets()` function will return every available option, however this can be unwieldy when the list of results is long, involving much scrolling to find the entry you are interested in. *biomaRt* also provides the functions `searchDatasets()` which will try to find any entries matching a specific term or pattern. For example, if we want to find the details of any datasets in our `ensembl` mart that contain the term ‘*hsapiens*’ we could do the following:

```
searchDatasets(mart = ensembl, pattern = "hsapiens")
```

```
##                  dataset              description    version
## 79 hsapiens_gene_ensembl Human genes (GRCh38.p13) GRCh38.p13
```

To use a dataset we can update our `Mart` object using the function `useDataset()`. In the example below we choose to use the *hsapiens* dataset.

```
ensembl <- useDataset(dataset = "hsapiens_gene_ensembl", mart = ensembl)
```

As mentioned previously, if the dataset one wants to use is known in advance i.e. you’ve gone through this process before, we can select a both the database and dataset in one step:

```
ensembl <- useEnsembl(biomart = "genes", dataset = "hsapiens_gene_ensembl")
```

## 2.3 Ensembl mirror sites

To improve performance Ensembl provides several mirrors of their site distributed around the globe. When you use the default settings for `useEnsembl()` your queries will be directed to your closest mirror geographically. In theory this should give you the best performance, however this is not always the case in practice. For example, if the nearest mirror is experiencing many queries from other users it may perform poorly for you. You can use the `mirror` argument to `useEnsembl()` to explicitly request a specific mirror.

```
ensembl <- useEnsembl(biomart = "ensembl", 
                   dataset = "hsapiens_gene_ensembl", 
                   mirror = "useast")
```

Values for the mirror argument are: `useast`, `uswest`, `asia`, and `www`.

## 2.4 Using archived versions of Ensembl

It is possible to query archived versions of Ensembl through *biomaRt*, so you can maintain consistent annotation throughout the duration of a project.

*biomaRt* provides the function `listEnsemblArchives()` to view the available Ensembl archives. This function takes no arguments, and produces a table containing the name and version number of the available archives, the date they were first released, and the URL where they can be accessed.

```
listEnsemblArchives()
```

```
##              name     date                                 url version current_release
## 1  Ensembl GRCh37 Feb 2014          https://grch37.ensembl.org  GRCh37                
## 2     Ensembl 109 Feb 2023 https://feb2023.archive.ensembl.org     109               *
## 3     Ensembl 108 Oct 2022 https://oct2022.archive.ensembl.org     108                
## 4     Ensembl 107 Jul 2022 https://jul2022.archive.ensembl.org     107                
## 5     Ensembl 106 Apr 2022 https://apr2022.archive.ensembl.org     106                
## 6     Ensembl 105 Dec 2021 https://dec2021.archive.ensembl.org     105                
## 7     Ensembl 104 May 2021 https://may2021.archive.ensembl.org     104                
## 8     Ensembl 103 Feb 2021 https://feb2021.archive.ensembl.org     103                
## 9     Ensembl 102 Nov 2020 https://nov2020.archive.ensembl.org     102                
## 10    Ensembl 101 Aug 2020 https://aug2020.archive.ensembl.org     101                
## 11    Ensembl 100 Apr 2020 https://apr2020.archive.ensembl.org     100                
## 12     Ensembl 99 Jan 2020 https://jan2020.archive.ensembl.org      99                
## 13     Ensembl 98 Sep 2019 https://sep2019.archive.ensembl.org      98                
## 14     Ensembl 97 Jul 2019 https://jul2019.archive.ensembl.org      97                
## 15     Ensembl 96 Apr 2019 https://apr2019.archive.ensembl.org      96                
## 16     Ensembl 95 Jan 2019 https://jan2019.archive.ensembl.org      95                
## 17     Ensembl 94 Oct 2018 https://oct2018.archive.ensembl.org      94                
## 18     Ensembl 93 Jul 2018 https://jul2018.archive.ensembl.org      93                
## 19     Ensembl 92 Apr 2018 https://apr2018.archive.ensembl.org      92                
## 20     Ensembl 80 May 2015 https://may2015.archive.ensembl.org      80                
## 21     Ensembl 77 Oct 2014 https://oct2014.archive.ensembl.org      77                
## 22     Ensembl 75 Feb 2014 https://feb2014.archive.ensembl.org      75                
## 23     Ensembl 54 May 2009 https://may2009.archive.ensembl.org      54
```

Alternatively, one can use the http://www.ensembl.org website to find an archived version. From the main page scroll down the bottom of the page, click on ‘view in Archive’ and select the archive you need.

*You will notice that there is an archive URL even for the current release of Ensembl. It can be useful to use this if you wish to ensure that script you write now will return exactly the same results in the future. Using `www.ensembl.org` will always access the current release, and so the data retrieved may change over time as new releases come out.*

Whichever method you use to find the URL of the archive you wish to query, copy the url and use that in the `host` argument as shown below to connect to the specified BioMart database. The example below shows how to query Ensembl 95.

```
listEnsembl(version = 95)
```

```
##         biomart               version
## 1         genes      Ensembl Genes 95
## 2 mouse_strains      Mouse strains 95
## 3          snps  Ensembl Variation 95
## 4    regulation Ensembl Regulation 95
```

```
ensembl95 <- useEnsembl(biomart = 'genes', 
                       dataset = 'hsapiens_gene_ensembl',
                       version = 95)
```

## 2.5 Using Ensembl Genomes

Ensembl Genomes expands the effort to provide annotation from the vertebrate genomes provided by the main Ensembl project across taxonomic space, with separate BioMart interfaces for Protists, Plants, Metazoa and Fungi. 222 Note: Unfortunately there is no BioMart interface to the Ensembl Bacteria data. The number of bacterial genomes is in the tens of thousands and BioMart does not perform well when providing data on that scale.

You can use the functions `listEnsemblGenomes()` and `useEnsemblGenomes()` in similar fashion to the functions shown previously. For example first we can list the available Ensembl Genomes marts:

```
listEnsemblGenomes()
```

```
##               biomart                        version
## 1       protists_mart      Ensembl Protists Genes 56
## 2 protists_variations Ensembl Protists Variations 56
## 3          fungi_mart         Ensembl Fungi Genes 56
## 4    fungi_variations    Ensembl Fungi Variations 56
## 5        metazoa_mart       Ensembl Metazoa Genes 56
## 6  metazoa_variations  Ensembl Metazoa Variations 56
## 7         plants_mart        Ensembl Plants Genes 56
## 8   plants_variations   Ensembl Plants Variations 56
```

We can the select the Ensembl Plants database, and search for the dataset name for Arabidopsis.

```
ensembl_plants <- useEnsemblGenomes(biomart = "plants_mart")
searchDatasets(ensembl_plants, pattern = "Arabidopsis")
```

```
##              dataset                         description version
## 4   ahalleri_eg_gene Arabidopsis halleri genes (Ahal2.2) Ahal2.2
## 5    alyrata_eg_gene    Arabidopsis lyrata genes (v.1.0)   v.1.0
## 10 athaliana_eg_gene Arabidopsis thaliana genes (TAIR10)  TAIR10
```

We can then use this information to create our `Mart` object that will access the correct database and dataset.

```
ensembl_arabidopsis <- useEnsemblGenomes(biomart = "plants_mart", 
                                         dataset = "athaliana_eg_gene")
```

# 3 How to build a biomaRt query

Once we’ve selected a dataset to get data from, we need to create a query and send it to the Ensembl BioMart server. We do this using the `getBM()` function.

The `getBM()` function has three arguments that need to be introduced: *filters*, *values* and *attributes*.

*Filters* and *values* are used to define restrictions on the query. For example, if you want to restrict the output to all genes located on the human X chromosome then the filter *chromosome\_name* can be used with *value* ‘X’. The `listFilters()` function shows you all available filters in the selected dataset.

```
filters = listFilters(ensembl)
filters[1:5,]
```

```
##              name              description
## 1 chromosome_name Chromosome/scaffold name
## 2           start                    Start
## 3             end                      End
## 4      band_start               Band Start
## 5        band_end                 Band End
```

*Attributes* define the data we are interested in retrieving. For example, maybe we want to retrieve the gene symbols or chromosomal coordinates. The `listAttributes()` function displays all available attributes in the selected dataset.

```
attributes = listAttributes(ensembl)
attributes[1:5,]
```

```
##                            name                  description         page
## 1               ensembl_gene_id               Gene stable ID feature_page
## 2       ensembl_gene_id_version       Gene stable ID version feature_page
## 3         ensembl_transcript_id         Transcript stable ID feature_page
## 4 ensembl_transcript_id_version Transcript stable ID version feature_page
## 5            ensembl_peptide_id            Protein stable ID feature_page
```

The `getBM()` function is the primary query function in *biomaRt*. It has four main arguments:

- `attributes`: is a vector of attributes that one wants to retrieve (= the output of the query).
- `filters`: is a vector of filters that one wil use as input to the query.
- `values`: a vector of values for the filters. In case multple filters are in use, the values argument requires a list of values where each position in the list corresponds to the position of the filters in the filters argument (see examples below).
- `mart`: is an object of class `Mart`, which is created by the `useEnsembl()` function.

*Note: for some frequently used queries to Ensembl, wrapper functions are available: `getGene()` and `getSequence()`. These functions call the `getBM()` function with hard coded filter and attribute names.*

Now that we selected a BioMart database and dataset, and know about attributes, filters, and the values for filters; we can build a *biomaRt* query. Let’s make an easy query for the following problem: We have a list of Affymetrix identifiers from the u133plus2 platform and we want to retrieve the corresponding EntrezGene identifiers using the Ensembl mappings.

The u133plus2 platform will be the filter for this query and as values for this filter we use our list of Affymetrix identifiers. As output (attributes) for the query we want to retrieve the EntrezGene and u133plus2 identifiers so we get a mapping of these two identifiers as a result. The exact names that we will have to use to specify the attributes and filters can be retrieved with the `listAttributes()` and `listFilters()` function respectively. Let’s now run the query:

```
affyids <- c("202763_at","209310_s_at","207500_at")
getBM(attributes = c('affy_hg_u133_plus_2', 'entrezgene_id'),
      filters = 'affy_hg_u133_plus_2',
      values = affyids, 
      mart = ensembl)
```

```
##   affy_hg_u133_plus_2 entrezgene_id
## 1         209310_s_at           837
## 2           207500_at           838
## 3           202763_at           836
```

## 3.1 Searching for filters and attributes

The functions `listAttributes()` and `listFilters()` will return every available option for their respective types, which can produce a very long output where it is hard to find the value you are interested in. *biomaRt* also provides the functions `searchAttributes()` and `searchFilters()` which will try to find any entries matching a specific term or pattern, in a similar fashion to `searchDatasets()` seen previously. You can use these functions to find available attributes and filters that you may be interested in. The example below returns the details for all attributes that contain the pattern ‘*hgnc*’.

```
searchAttributes(mart = ensembl, pattern = "hgnc")
```

```
##               name        description         page
## 62         hgnc_id            HGNC ID feature_page
## 63     hgnc_symbol        HGNC symbol feature_page
## 93 hgnc_trans_name Transcript name ID feature_page
```

For advanced use, note that the *pattern* argument takes a regular expression. This means you can create more complex queries if required. Imagine, for example, that we have the string *ENST00000577249.1*, which we know is an Ensembl ID of some kind, but we aren’t sure what the appropriate filter term is. The example shown next uses a pattern that will find all filters that contain the terms ‘*ensembl*’ and ‘*id*’. This allows us to reduced the list of filters to only those that might be appropriate for our example.

```
searchFilters(mart = ensembl, pattern = "ensembl.*id")
```

```
##                             name                                                    description
## 54               ensembl_gene_id                       Gene stable ID(s) [e.g. ENSG00000000003]
## 55       ensembl_gene_id_version       Gene stable ID(s) with version [e.g. ENSG00000000003.15]
## 56         ensembl_transcript_id                 Transcript stable ID(s) [e.g. ENST00000000233]
## 57 ensembl_transcript_id_version Transcript stable ID(s) with version [e.g. ENST00000000233.10]
## 58            ensembl_peptide_id                    Protein stable ID(s) [e.g. ENSP00000000233]
## 59    ensembl_peptide_id_version     Protein stable ID(s) with version [e.g. ENSP00000000233.5]
## 60               ensembl_exon_id                              Exon ID(s) [e.g. ENSE00000000001]
```

From this we can compare *ENST00000577249.1* with the examples given in the description column, and see it is a Transcript ID with version. Thus the appropriate filter value to use with it is `ensembl_transcript_id_version`.

## 3.2 Using predefined filter values

Many filters have a predefined list of values that are known to be in the dataset they are associated with. A common example would be the names of chromosomes when searching a dataset at Ensembl. In this online interface to BioMart these available options are displayed as a list as shown in Figure 1.

Figure 1: The options available to the Chromosome/Scaffold field are limited to a pretermined list based on the values in this dataset

You can list this in an R session with the function `listFilterOptions()`, passing it a `Mart` object and the name of the filter. For example, to list the possible chromosome names you could run the following:

```
listFilterOptions(mart = ensembl, filter = "chromosome_name")
```

It is also possible to search the list of available values via `searchFilterOptions()`. In the two examples below, the first returns all chromosome names starting with “*GL*”, while the second will find any phenotype descriptions that contain the string “*Crohn*”.

```
searchFilterOptions(mart = ensembl, filter = "chromosome_name", 
                    pattern = "^GL")
```

```
##  [1] "GL000009.2" "GL000194.1" "GL000195.1" "GL000205.2" "GL000213.1" "GL000216.2" "GL000218.1"
##  [8] "GL000219.1" "GL000220.1" "GL000225.1"
```

```
searchFilterOptions(mart = ensembl, filter = "phenotype_description", 
                    pattern = "Crohn")
```

```
## [1] "INFLAMMATORY BOWEL DISEASE CROHN DISEASE 1"  "INFLAMMATORY BOWEL DISEASE CROHN DISEASE 10"
## [3] "INFLAMMATORY BOWEL DISEASE CROHN DISEASE 19" "INFLAMMATORY BOWEL DISEASE CROHN DISEASE 30"
## [5] "NON RARE IN EUROPE: Crohn disease"
```

## 3.3 Finding out more information on filters

### 3.3.1 filterType

Boolean filters need a value TRUE or FALSE in *biomaRt*. Setting the value TRUE will include all information that fulfil the filter requirement. Setting FALSE will exclude the information that fulfills the filter requirement and will return all values that don’t fulfil the filter. For most of the filters, their name indicates if the type is a boolean or not and they will usually start with “with”. However this is not a rule and to make sure you got the type right you can use the function `filterType()` to investigate the type of the filter you want to use.

```
filterType("with_affy_hg_u133_plus_2", ensembl)
```

```
## [1] "boolean_list"
```

## 3.4 Attribute Pages

For large BioMart databases such as Ensembl, the number of attributes displayed by the `listAttributes()` function can be very large.
In BioMart databases, attributes are put together in pages, such as sequences, features, homologs for Ensembl.
An overview of the attributes pages present in the respective BioMart dataset can be obtained with the `attributePages()` function.

```
pages = attributePages(ensembl)
pages
```

```
## [1] "feature_page" "structure"    "homologs"     "snp"          "snp_somatic"  "sequences"
```

To show us a smaller list of attributes which belong to a specific page, we can now specify this in the `listAttributes()` function.333 The set of attributes is still quite long, so we use `head()` to show only the first few items here.

```
head(listAttributes(ensembl, page="feature_page"))
```

```
##                            name                  description         page
## 1               ensembl_gene_id               Gene stable ID feature_page
## 2       ensembl_gene_id_version       Gene stable ID version feature_page
## 3         ensembl_transcript_id         Transcript stable ID feature_page
## 4 ensembl_transcript_id_version Transcript stable ID version feature_page
## 5            ensembl_peptide_id            Protein stable ID feature_page
## 6    ensembl_peptide_id_version    Protein stable ID version feature_page
```

We now get a short list of attributes related to the region where the genes are located.

## 3.5 Using `select()`

In order to provide a more consistent interface to all annotations in Bioconductor the `select()`, `columns()`, `keytypes()` and `keys()` have been implemented to wrap some of the existing functionality above. These methods can be called in the same manner that they are used in other parts of the project except that instead of taking a `AnnotationDb` derived class they take instead a `Mart` derived class as their 1st argument. Otherwise usage should be essentially the same. You still use `columns()` to discover things that can be extracted from a `Mart`, and `keytypes()` to discover which things can be used as keys with `select()`.

```
mart <- useEnsembl(dataset = "hsapiens_gene_ensembl", biomart='ensembl')
head(keytypes(mart), n = 3)
```

```
## [1] "affy_hc_g110"        "affy_hg_focus"       "affy_hg_u133_plus_2"
```

```
head(columns(mart), n = 3)
```

```
## [1] "3_utr_end"   "3_utr_end"   "3_utr_start"
```

And you still can use `keys()` to extract potential keys, for a
particular key type.

```
k = keys(mart, keytype="chromosome_name")
head(k, n=3)
```

```
## [1] "1" "2" "3"
```

When using `keys()`, you can even take advantage of the extra
arguments that are available for others keys methods.

```
k = keys(mart, keytype="chromosome_name", pattern="LRG")
head(k, n=3)
```

```
## character(0)
```

Unfortunately the `keys()` method will not work with all key types because they are not all supported.

But you can still use `select()` here to extract columns of data that match a particular set of keys (this is basically a wrapper for `getBM()`).

```
affy=c("202763_at","209310_s_at","207500_at")
select(mart, keys=affy, columns=c('affy_hg_u133_plus_2','entrezgene_id'),
  keytype='affy_hg_u133_plus_2')
```

```
##   affy_hg_u133_plus_2 entrezgene_id
## 1         209310_s_at           837
## 2           207500_at           838
## 3           202763_at           836
```

So why would we want to do this when we already have functions like `getBM()`? For two reasons: 1) for people who are familiar
with select and it’s helper methods, they can now proceed to use *biomaRt* making the same kinds of calls that are already familiar to them and 2) because the select method is implemented in many places elsewhere, the fact that these methods are shared allows for more convenient programmatic access of all these resources. An example of a package that takes advantage of this is the *OrganismDbi* package. Where several packages can be accessed as if they were one resource.

# 4 Result Caching

To save time and computing resources *biomaRt* will attempt to identify when you are re-running a query you have executed before. Each time a new query is run, the results will be saved to a cache on your computer. If a query is identified as having been run previously, rather than submitting the query to the server, the results will be loaded from the cache.

You can get some information on the size and location of the cache using the function `biomartCacheInfo()`:

```
biomartCacheInfo()
```

```
## biomaRt cache
## - Location: ~/.cache/biomaRt
## - No. of files: 1104
## - Total size: 223.5 Mb
```

The cache can be deleted using the command `biomartCacheClear()`. This will remove all cached files.

The default location for the cache is specifc to your computer and operating system. If you want to use a specific location you can set this via the `BIOMART_CACHE` environment variable. You can either set this outside of R, or within R via a call to `Sys.setenv(BIOMART_CACHE = "</where/i/store/my/cache>")`. The code below gives an example where we change the location to a temporary file444 This would not be a sensible choice on your machine, but is convenient on the Bioconductor server. and then confirm that the location has changed.

```
Sys.setenv(BIOMART_CACHE = tempdir())
biomartCacheInfo()
```

```
## biomaRt cache
## - Location: /tmp/Rtmp8M725C
## - No. of files: 0
## - Total size: 0 bytes
```

# 5 biomaRt helper functions

This section describes a set of *biomaRt* helper functions that can be used to export FASTA format sequences, retrieve values for certain filters and exploring the available filters and attributes in a more systematic manner.

## 5.1 exportFASTA

The data.frames obtained by the `getSequence()` function can be exported to FASTA files using the `exportFASTA()` function. One has to specify the data.frame to export and the filename using the file argument.

# 6 Examples of biomaRt queries

In the sections below a variety of example queries are described. Every example is written as a task, and we have to come up with a *biomaRt* solution to the problem.

## 6.1 Annotate a set of Affymetrix identifiers with HUGO symbol and chromosomal locations of corresponding genes

We have a list of Affymetrix hgu133plus2 identifiers and we would like to retrieve the HUGO gene symbols, chromosome names, start and end positions and the bands of the corresponding genes. The `listAttributes()` and the `listFilters()` functions give us an overview of the available attributes and filters and we look in those lists to find the corresponding attribute and filter names we need. For this query we’ll need the following attributes: hgnc\_symbol, chromsome\_name, start\_position, end\_position, band and affy\_hg\_u133\_plus\_2 (as we want these in the output to provide a mapping with our original Affymetrix input identifiers. There is one filter in this query which is the affy\_hg\_u133\_plus\_2 filter as we use a list of Affymetrix identifiers as input. Putting this all together in the `getBM()` and performing the query gives:

```
affyids=c("202763_at","209310_s_at","207500_at")
getBM(attributes = c('affy_hg_u133_plus_2', 'hgnc_symbol', 'chromosome_name',
                   'start_position', 'end_position', 'band'),
      filters = 'affy_hg_u133_plus_2', 
      values = affyids, 
      mart = ensembl)
```

```
##   affy_hg_u133_plus_2 hgnc_symbol chromosome_name start_position end_position  band
## 1         209310_s_at       CASP4              11      104942866    104969366 q22.3
## 2           207500_at       CASP5              11      104994235    105023168 q22.3
## 3           202763_at       CASP3               4      184627696    184650062 q35.1
```

## 6.2 Annotate a set of EntrezGene identifiers with GO annotation

In this task we start out with a list of EntrezGene identiers and we want to retrieve GO identifiers related to biological processes that are associated with these entrezgene identifiers. Again we look at the output of `listAttributes()` and `listFilters()` to find the filter and attributes we need. Then we construct the following query:

```
entrez=c("673","837")
goids = getBM(attributes = c('entrezgene_id', 'go_id'), 
              filters = 'entrezgene_id', 
              values = entrez, 
              mart = ensembl)
head(goids)
```

```
##   entrezgene_id      go_id
## 1           673 GO:0005524
## 2           673 GO:0007165
## 3           673 GO:0006468
## 4           673 GO:0004672
## 5           673 GO:0043231
## 6           673 GO:0046872
```

## 6.3 Retrieve all HUGO gene symbols of genes that are located on chromosomes 17,20 or Y, and are associated with specific GO terms

The GO terms we are interested in are: **GO:0051330**, **GO:0000080**, **GO:0000114**, **GO:0000082**. The key to performing this query is to understand that the `getBM()` function enables you to use more than one filter at the same time. In order to do this, the filter argument should be a vector with the filter names. The values should be a list, where the first element of the list corresponds to the first filter and the second list element to the second filter and so on. The elements of this list are vectors containing the possible values for the corresponding filters.

```
 go=c("GO:0051330","GO:0000080","GO:0000114","GO:0000082")
 chrom=c(17,20,"Y")
 getBM(attributes= "hgnc_symbol",
        filters=c("go","chromosome_name"),
        values=list(go, chrom), mart=ensembl)
```

```
##   hgnc_symbol
## 1        E2F1
## 2     RPS6KB1
## 3        CDK3
```

## 6.4 Annotate set of idenfiers with INTERPRO protein domain identifiers

In this example we want to annotate the following two RefSeq identifiers: **NM\_005359** and **NM\_000546** with INTERPRO protein domain identifiers and a description of the protein domains.

```
refseqids = c("NM_005359","NM_000546")
ipro = getBM(attributes=c("refseq_mrna","interpro","interpro_description"), 
             filters="refseq_mrna",
             values=refseqids, 
             mart=ensembl)
ipro
```

```
##    refseq_mrna  interpro                                               interpro_description
## 1    NM_000546 IPR002117                                       p53 tumour suppressor family
## 2    NM_000546 IPR008967                         p53-like transcription factor, DNA-binding
## 3    NM_000546 IPR010991                                        p53, tetramerisation domain
## 4    NM_000546 IPR011615                                            p53, DNA-binding domain
## 5    NM_000546 IPR012346 p53/RUNT-type transcription factor, DNA-binding domain superfamily
## 6    NM_000546 IPR013872                                         p53 transactivation domain
## 7    NM_000546 IPR036674                        p53-like tetramerisation domain superfamily
## 8    NM_000546 IPR040926               Cellular tumor antigen p53, transactivation domain 2
## 9    NM_005359 IPR001132                                          SMAD domain, Dwarfin-type
## 10   NM_005359 IPR003619                                       MAD homology 1, Dwarfin-type
## 11   NM_005359 IPR008984                                        SMAD/FHA domain superfamily
## 12   NM_005359 IPR013019                                                  MAD homology, MH1
## 13   NM_005359 IPR013790                                                            Dwarfin
## 14   NM_005359 IPR017855                                       SMAD-like domain superfamily
## 15   NM_005359 IPR036578                                        SMAD MH1 domain superfamily
```

## 6.5 Select all Affymetrix identifiers on the hgu133plus2 chip and Ensembl gene identifiers for genes located on chromosome 16 between basepair 1100000 and 1250000.

In this example we will again use multiple filters: *chromosome\_name*, *start*, and *end* as we filter on these three conditions. Note that when a chromosome name, a start position and an end position are jointly used as filters, the BioMart webservice interprets this as return everything from the given chromosome between the given start and end positions.

```
getBM(attributes = c('affy_hg_u133_plus_2','ensembl_gene_id'), 
      filters = c('chromosome_name','start','end'),
      values = list(16,1100000,1250000), 
      mart = ensembl)
```

```
##    affy_hg_u133_plus_2 ensembl_gene_id
## 1                      ENSG00000260702
## 2            215502_at ENSG00000260532
## 3                      ENSG00000273551
## 4            205845_at ENSG00000196557
## 5                      ENSG00000196557
## 6                      ENSG00000260403
## 7                      ENSG00000259910
## 8                      ENSG00000261294
## 9          220339_s_at ENSG00000116176
## 10                     ENSG00000277010
## 11         215382_x_at ENSG00000197253
## 12         205683_x_at ENSG00000197253
## 13         210084_x_at ENSG00000197253
## 14         207134_x_at ENSG00000197253
## 15         216474_x_at ENSG00000197253
## 16         217023_x_at ENSG00000197253
## 17         215382_x_at ENSG00000172236
## 18         205683_x_at ENSG00000172236
## 19         210084_x_at ENSG00000172236
## 20         207134_x_at ENSG00000172236
## 21         216474_x_at ENSG00000172236
## 22         217023_x_at ENSG00000172236
```

## 6.6 Retrieve all EntrezGene identifiers and HUGO gene symbols of genes which have a “MAP kinase activity” GO term associated with it.

The GO identifier for MAP kinase activity is **GO:0004707**. In our query we will use *go\_id* as our filter, and *entrezgene\_id* and *hgnc\_symbol* as attributes. Here’s the query:

```
getBM(attributes = c('entrezgene_id','hgnc_symbol'), 
      filters = 'go', 
      values = 'GO:0004707', 
      mart = ensembl)
```

```
##    entrezgene_id hgnc_symbol
## 1         225689      MAPK15
## 2           5596       MAPK4
## 3           5594       MAPK1
## 4           6300      MAPK12
## 5           5600      MAPK11
## 6           5595       MAPK3
## 7           5602      MAPK10
## 8           5609      MAP2K7
## 9           5601       MAPK9
## 10          5599       MAPK8
## 11          5598       MAPK7
## 12          5603      MAPK13
## 13          5597       MAPK6
## 14          6885      MAP3K7
## 15          1432      MAPK14
## 16         51701         NLK
```

## 6.7 Given a set of EntrezGene identifiers, retrieve 100bp upstream promoter sequences

All sequence related queries to Ensembl are available through the `getSequence()` wrapper function. `getBM()` can also be used directly to retrieve sequences but this can get complicated so using `getSequence()` is provided as a general purpose function suitable for most situtations.

Sequences can be retrieved using the `getSequence()` function either starting from chromosomal coordinates or identifiers.

- *cdna*
- *peptide* for protein sequences
- *3utr* for 3’ UTR sequences
- *5utr* for 5’ UTR sequences
- *gene\_exon* for exon sequences only
- *transcript\_exon* for transcript specific exonic sequences only
- *transcript\_exon\_intron* gives the full unspliced transcript, that is exons + introns
- *gene\_exon\_intron* gives the exons + introns of a gene
- *coding* gives the coding sequence only
- *coding\_transcript\_flank* gives the flanking region of the transcript including the UTRs, this must be accompanied with a given value for the upstream or downstream attribute
- *coding\_gene\_flank* gives the flanking region of the gene including the UTRs, this must be accompanied with a given value for the upstream or downstream attribute
- *transcript\_flank* gives the flanking region of the transcript exculding the UTRs, this must be accompanied with a given value for the upstream or downstream attribute
- *gene\_flank* gives the flanking region of the gene excluding the UTRs, this must be accompanied with a given value for the upstream or downstream attribute

This task requires us to retrieve 100bp upstream promoter sequences from a set of EntrezGene identifiers. The *type* argument in `getSequence()` can be thought of as the filter in this query and uses the same input names given by `listFilters()`. In our query we use `entrezgene_id` for the type argument. Next we have to specify which type of sequences we want to retrieve, here we are interested in the sequences of the promoter region, starting right next to the coding start of the gene. Setting the *seqType* to `coding_gene_flank` will give us what we need. The *upstream* argument is used to specify how many bp of upstream sequence we want to retrieve, here we’ll retrieve a rather short sequence of 100bp. Putting this all together in `getSequence()` gives:

```
entrez=c("673","7157","837")
getSequence(id = entrez, 
            type="entrezgene_id",
            seqType="coding_gene_flank",
            upstream=100, 
            mart=ensembl)
```

```
##                                                                                      coding_gene_flank
## 1 TCCTTCTCTGCAGGCCCAGGTGACCCAGGGTTGGAAGTGTCTCATGCTGGATCCCCACTTTTCCTCTTGCAGCAGCCAGACTGCCTTCCGGGTCACTGCC
## 2 CCTCCGCCTCCGCCTCCGCCTCCGCCTCCCCCAGCTCTCCGCCTCCCTTCCCCCTCCCCGCCCGACAGCGGCCGCTCGGGCCCCGGCTCTCGGTTATAAG
## 3 CACGTTTCCGCCCTTTGCAATAAGGAAATACATAGTTTACTTTCATTTTTGACTCTGAGGCTCTTTCCAACGCTGTAAAAAAGGACAGAGGCTGTTCCCT
##   entrezgene_id
## 1          7157
## 2           673
## 3           837
```

One further thing to note is that, although we are searching for genes based on their NCBI Gene IDs, Ensembl BioMart doesn’t allow some ID types (including NCBI IDs) to be returned directly. To try and accommodate this *biomaRt* attempts to map the query IDs to Ensembl Gene IDs internally before finding the sequence information. If no such mapping exists (or at least isn’t found in Ensembl) then no sequence will be returned for the affected IDs.

## 6.8 Retrieve all 5’ UTR sequences of all genes that are located on chromosome 3 between the positions 185,514,033 and 185,535,839

As described in the previous task `getSequence()` can also use chromosomal coordinates to retrieve sequences of all genes that lie in the given region. We also have to specify which type of identifier we want to retrieve together with the sequences. Here we choose the NCBI Gene ID555 These were historically called “Entrezgene IDs”, hence the name given to the `type` argument.

```
utr5 = getSequence(chromosome=3, start=185514033, end=185535839,
                   type="entrezgene_id",
                   seqType="5utr", 
                   mart=ensembl)
utr5
```

```
##                                                                                                                    5utr
## 1                                                                               ATTCTTGTGAATGTGACACACGATCTCTCCAGTTTCCAT
## 2                               TGAGCAAAATCCCACAGTGGAAACTCTTAAGCCTCTGCGAAGTAAATCATTCTTGTGAATGTGACACACGATCTCTCCAGTTTCCAT
## 3                                                                                                  Sequence unavailable
## 4 ACCACACCTCTGAGTCGTCTGAGCTCACTGTGAGCAAAATCCCACAGTGGAAACTCTTAAGCCTCTGCGAAGTAAATCATTCTTGTGAATGTGACACACGATCTCTCCAGTTTCCAT
##   entrezgene_id
## 1        200879
## 2        200879
## 3        200879
## 4        200879
```

## 6.9 Retrieve protein sequences for a given list of EntrezGene identifiers

In this task the type argument specifies which type of identifiers we are using.
To get an overview of other valid identifier types we refer to the `listFilters()` function.

```
protein = getSequence(id=c(100, 5728),
                      type="entrezgene_id",
                      seqType="peptide", 
                      mart=ensembl)
protein
```

```
##                                                                                                                                                                                                                                                                                                                                                                                                                                                                                                                                                                                              peptide
## 1                                                                                                                                                                               MTAIIKEIVSRNKRRYQEDGFDLDLTYIYPNIIAMGFPAERLEGVYRNNIDDVVRFLDSKHKNHYKIYNLCAERHYDTAKFNCRVAQYPFEDHNPPQLELIKPFCEDLDQWLSEDDNHVAAIHCKAGKGRTGVMICAYLLHRGKFLKAQEALDFYGEVRTRDKKGVTIPSQRRYVYYYSYLLKNHLDYRPVALLFHKMMFETIPMFSGGTCNPQFVVCQLKVKIYSSNSGPTRREDKFMYFEFPQPLPVCGDIKVEFFHKQNKMLKKDKMFHFWVNTFFIPGPEETSEKVENGSLCDQEIDSICSIERADNDKEYLVLTLTKNDLDKANKDKANRYFSPNFKVKLYFTKTVEEPSNPEASSSTSVTPDVSDNEPDHYRYSDTTDSDPENEPFDEDQHTQITKV*
## 2                                                                                                                                                                                                                                                                                                                                                                                                                                                                                                                         MTAIIKEIVSRNKRRYQEDGFDLDLTYIYPNIIAMGFPAERLEGVYRNNIDDVVRFLDSKHKNHYKIYNLWGI*
## 3                                                                                                                                                                                                                                                                                                                                                                                                                                                                                                                                                                               Sequence unavailable
## 4  LERGGEAAAAAAAAAAAPGRGSESPVTISRAGNAGELVSPLLLPPTRRRRRRHIQGPGPVLNLPCAAAAPPVARAPEAAGGGSRSEDYSSSPHSAAAAARPLAAEEKQAQSLQPSSSRRSSHYPAAVQSQAAAERGASATAKSRAISILQKKPRHQQLLPSLSSFFFSHRLPDMTAIIKEIVSRNKRRYQEDGFDLDLTYIYPNIIAMGFPAERLEGVYRNNIDDVVRFLDSKHKNHYKIYNLCAERHYDTAKFNCRVAQYPFEDHNPPQLELIKPFCEDLDQWLSEDDNHVAAIHCKAGKGRTGVMICAYLLHRGKFLKAQEALDFYGEVRTRDKKGVTIPSQRRYVYYYSYLLKNHLDYRPVALLFHKMMFETIPMFSGGTCNPQFVVCQLKVKIYSSNSGPTRREDKFMYFEFPQPLPVCGDIKVEFFHKQNKMLKKDKMFHFWVNTFFIPGPEETSEKVENGSLCDQEIDSICSIERADNDKEYLVLTLTKNDLDKANKDKANRYFSPNFKVKLYFTKTVEEPSNPEASSSTSVTPDVSDNEPDHYRYSDTTDSDPENEPFDEDQHTQITKV*
## 5                                                                                                                                                                                                                                                                                                                                                                                                    MTAIIKEIVSRNKRRYQEDGFDLDLTYIYPNIIAMGFPAERLEGVYRNNIDDVVRFLDSKHKNHYKIYNLCAERHYDTAKFNCRVAQYPFEDHNPPQLELIKPFCEDLDQWLSEDDNHVAAIHCKAGKGRTGVMICAYLLHRGKFLKAQEALDFYGEVRTRDKKADPTGGIPDKGIIVIGDGSSMDVIAP*
## 6                                                                                                                                                                                                                                          MTAIIKEIVSRNKRRYQEDGFDLDLTYIYPNIIAMGFPAERLEGVYRNNIDDVVRFLDSKHKNHYKIYNLCAERHYDTAKFNCRVAQYPFEDHNPPQLELIKPFCEDLDQWLSEDDNHVAAIHCKAGKGRTGVMICAYLLHRGKFLKAQEALDFYGEVRTRDKKGVTIPSQRRYVYYYSYLLKNHLDYRPVALLFHKMMFETIPMFSGGTCNPQFVVCQLKVKIYSSNSGPTRREDKFMYFEFPQPLPVCGDIKVEFFHKQNKMLKKDKMFHFWVNTFFIPGPEETSEKVENGSLCDQEIDSICSIERADNDKEYLVLTLTKNDLDKANKDKANRYFSPNFKVS*
## 7                                                                                                                                                                                              MTAIIKEIVSRNKRRYQEDGFDLDLTYIYPNIIAMGFPAERLEGVYRNNIDDVVSCAERHYDTAKFNCRVAQYPFEDHNPPQLELIKPFCEDLDQWLSEDDNHVAAIHCKAGKGRTGVMICAYLLHRGKFLKAQEALDFYGEVRTRDKKGVTIPSQRRYVYYYSYLLKNHLDYRPVALLFHKMMFETIPMFSGGTCNPQFVVCQLKVKIYSSNSGPTRREDKFMYFEFPQPLPVCGDIKVEFFHKQNKMLKKDKMFHFWVNTFFIPGPEETSEKVENGSLCDQEIDSICSIERADNDKEYLVLTLTKNDLDKANKDKANRYFSPNFKVKLYFTKTVEEPSNPEASSSTSVTPDVSDNEPDHYRYSDTTDSDPENEPFDEDQHTQITKV*
## 8                                                                                                                                                                                                                                                                                                                                                                                                                                                                                                                                                     MRDGGGRGPEPLSAPVSSRGGSALGEPAGLRRRQRRRFSPPLRLF*
## 9                                                                                                                                                                                                                                                                                                                      XLDSKHKNHYKIYNLCAERHYDTAKFNCRVAQYPFEDHNPPQLELIKPFCEDLDQWLSEDDNHVAAIHCKAGKGRTGVMICAYLLHRGKFLKAQEALDFYGEVRTRDKKGVTIPSQRRYVYYYSYLLKNHLDYRPVALLFHKMMFETIPMFSGGTCNPQFVVCQLKVKIYSSNSGPTRREDKFMYFEFPQPLPVCGDIKVEFFHKQNKMLKKGQGLTMLPRLVSNSWAQAVLTLWHPKELELQDKMFHFWVNTFFIPGPEETSEKVENG
## 10                                                                                                                                                                                                                                                                                                                                                                                                                                                                                                                                                     MTAIIKEIVSRNKRRYQEDGFDLDLTCLFEEDSCVLWLIVKVLL*
## 11                                                                                                                                                                                                                                                                                                                                                                                                                     MTAIIKEIVSRNKRRYQEDGFDLDLTYIYPNIIAMGFPAERLEGVYRNNIDDVVRFLDSKHKNHYKIYNLCAERHYDTAKFNCRVAQYPFEDHNPPQLELIKPFCEDLDQWLSEDDNHVAAIHCKAGKGRTGVMICAYLLHRGKFLKAQEALDFYGEVRTRDKKILSLWSAS*
## 12                                                                                                                                                                                                                                                                                                                                                                                                                                                                                                                                                                              Sequence unavailable
## 13                                                                                                                                                                                                                                              MAQTPAFDKPKVELHVHLDGSIKPETILYYGRRRGIALPANTAEGLLNVIGMDKPLTLPDFLAKFDYYMPAIAGCREAIKRIAYEFVEMKAKEGVVYVEVRYSPHLLANSKVEPIPWNQAEGDLTPDEVVALVGQGLQEGERDFGVKARSILCCMRHQPNWSPKVVELCKKYQQQTVVAIDLAGDETIPGSSLLPGHVQAYQAVDILKTERLGHGYHTLEDQALYNRLRQENMHFEICPWSSYLTGAWKPDTEHAVIRLKNDQANYSLNTDDPLIFKSTLDTDYQMTKRDMGFTEEEFKRLNINAAKSSFLPEDEKRELLDLLYKAYGMPPSASAGQNL*
## 14                                                                                                                                                                                                                                                                                                                                                                                                     MAQTPAFDKPKVELHVHLDGSIKPETILYYGRRRGIALPANTAEGLLNVIGMDKPLTLPDFLAKFDYYMPAIALWTYSRQSGWDTATTPWKTRPFITGCGRKTCTSRLKNDQANYSLNTDDPLIFKSTLDTDYQMTKRDMGFTEEEFKRLNINAAKSSFLPEDEKRELLDLLYKAYGMPPSASAGQNL*
## 15                                                                                                                                                                                                                                                                                                                          MAQTPAFDKPKVELHVHLDGSIKPETILYYGRRRGIALPANTAEGLLNVIGMDKPLTLPDFLAKFDYYMPAIAGCREAIKRIAYEFVEMKAKEGVVYVEVRYSPHLLANSKVEPIPWNQAEGDLTPDEVVALVGQGLQEGERDFGVKARSILCCMRHQPNWSPKVVELCKKYQQQTVVAIDLAGDETIPGSSLLPGHVQAYQEAVKSGIHRTVHAGEVGSAEVVKEAVDILKTERLGHGYHTLEDQALYNRLRQENMHFEAQK*
## 16                                                                                                                                                                                                                                                                                                                                                                                                                                     MAQTPAFDKPKVELHVHLDGSIKPETILYYGRRRGIALPANTAEGLLNVIGMDKPLTLPDFLAKFDYYMPAIALWTYSRQSGWDTATTPWKTRPFITGCGRKTCTSRSAPGPATSLVPGSRTRSMQSFGSKMTRLTTRSTQMTRSSSSPPWTLITR*
## 17                                                                                                                                                                                                                                                                                                                                                                                                                                                                                                                      MAQTPAFDKPKVELHVHLDGSIKPETILYYGRRRGIALPANTAEGLLNVIGMDKPLTLPDFLAKFDYYMPAIARL*
## 18                                                                                                                                                                                                                                                                         MDKPLTLPDFLAKFDYYMPAIAGCREAIKRIAYEFVEMKAKEGVVYVEVRYSPHLLANSKVEPIPWNQAEGDLTPDEVVALVGQGLQEGERDFGVKARSILCCMRHQPNWSPKVVELCKKYQQQTVVAIDLAGDETIPGSSLLPGHVQAYQEAVKSGIHRTVHAGEVGSAEVVKEAVDILKTERLGHGYHTLEDQALYNRLRQENMHFEICPWSSYLTGAWKPDTEHAVIRLKNDQANYSLNTDDPLIFKSTLDTDYQMTKRDMGFTEEEFKRLNINAAKSSFLPEDEKRELLDLLYKAYGMPPSASAGQNL*
## 19                                                                                                                                                                                                                                               MAQTPAFDKPKVELHVHLDGSIKPETILYYGRRGIALPANTAEGLLNVIGMDKPLTLPDFLAKFDYYMPAIAGCREAIKRIAYEFVEMKAKEGVVYVEVRYSPHLLANSKVEPIPWNQAEGDLTPDEVVALVGQGLQEGERDFGVKARSILCCMRHQPNWSPKVVELCKKYQQQTVVAIDLAGDETIPGSSLLPGHVQAYQAVDILKTERLGHGYHTLEDQALYNRLRQENMHFEICPWSSYLTGAWKPDTEHAVIRLKNDQANYSLNTDDPLIFKSTLDTDYQMTKRDMGFTEEEFKRLNINAAKSSFLPEDEKRELLDLLYKAYGMPPSASAGQNL*
## 20                                                                                                                                                                MAQTPAFDKPKVELHVHLDGSIKPETILYYGRRGIALPANTAEGLLNVIGMDKPLTLPDFLAKFDYYMPAIAGCREAIKRIAYEFVEMKAKEGVVYVEVRYSPHLLANSKVEPIPWNQAEGDLTPDEVVALVGQGLQEGERDFGVKARSILCCMRHQPNWSPKVVELCKKYQQQTVVAIDLAGDETIPGSSLLPGHVQAYQEAVKSGIHRTVHAGEVGSAEVVKEAVDILKTERLGHGYHTLEDQALYNRLRQENMHFEICPWSSYLTGAWKPDTEHAVIRLKNDQANYSLNTDDPLIFKSTLDTDYQMTKRDMGFTEEEFKRLNINAAKSSFLPEDEKRELLDLLYKAYGMPPSASAGRFLSGLLGSCPVLAPVWLSVGLLARCPSILGQRHECVMTPWFLGPGWEQRLIRSVCFL*
## 21                                                                                                                                                                                            MSTPDRHLVLCPAPTECSVNGSSFVRQRYGGERQGAQVELHVHLDGSIKPETILYYGRRRGIALPANTAEGLLNVIGMDKPLTLPDFLAKFDYYMPAIAGCREAIKRIAYEFVEMKAKEGVVYVEVRYSPHLLANSKVEPIPWNQAEGDLTPDEVVALVGQGLQEGERDFGVKARSILCCMRHQPNWSPKVVELCKKYQQQTVVAIDLAGDETIPGSSLLPGHVQAYQEAVKSGIHRTVHAGEVGSAEVVKEAVDILKTERLGHGYHTLEDQALYNRLRQENMHFEICPWSSYLTGAWKPDTEHAVIRLKNDQANYSLNTDDPLIFKSTLDTDYQMTKRDMGFTEEEFKRLNINAAKSSFLPEDEKRELLDLLYKAYGMPPSASAGQNL*
## 22                                                                                                                                                                                                                                                                                                                                                                                                                                                                          VELHVHLDGSIKPETILYYGRRRGIALPANTAEGLLNVIGMDKPLTLPDFLAKFDYYMPAIAGCREAIKRIAYEFVEMKAKEGVVYVEVRYSPHLLANSKVEPIPWNQAELVPQGGGAV*
## 23                                                                                                                                                                                                                                                                                                                                                        MAQTPAFDKPKVELHVHLDGSIKPETILYYGRRRGIALPANTAEGLLNVIGMDKPLTLPDFLAKFDYYMPAIEAVKSGIHRTVHAGEVGSAEVVKEAVDILKTERLGHGYHTLEDQALYNRLRQENMHFEICPWSSYLTGAWKPDTEHAVIRLKNDQANYSLNTDDPLIFKSTLDTDYQMTKRDMGFTEEEFKRLNINAAKSSFLPEDEKRELLDLLYKAYGMPPSASAGQNL*
## 24                                                                                                                                                                                                                                                                                                                                                                                                                                                                    XLKNDQANYSLNTDDPLIFKSTLDTDYQMTKRDMGFTEEEFKRLNINAAKSSFLPEDEKRELLDLLYKAYGMPPSASAEPTSRAEPLKTPLLQAFTLWSHPNSVGLSNIFTFIPSKKTMISIVSY*
## 25                                                                                                                                                                                                                                                                                                                                   MAQTPAFDKPKVELHVHLDGSIKPETILYYGRRRGIALPANTAEGLLNVIGMDKPLTLPDFLAKFDYYMPAIAGCREAIKRIAYEFVEMKAKEGVVYVEVRYSPHLLANSKVEPIPWNQAEGDLTPDEVVALVGQGLQEGERDFGVKARSILCCMRHQPNWSPKVVELCKKYQQQTVVAIDLAGDETIPGSSLLPGHVQAYQEAVKSGIHRTVHAGEAVDILKTERLGHGYHTLEDQALYNRLRQENMHFEAQK*
## 26                                                                                                                                                                                                                                                                                                                                                      XWSPKVVELCKKYQQQTVVAIDLAGDETIPGSSLLPGHVQAYQAVDILKTERLGHGYHTLEDQALYNRLRQENMHFEICPWSSYLTGAWKPDTEHAVIRLKNDQANYSLNTDDPLIFKSTLDTDYQMTKRDMGFTEEEFKRLNINAAKSSFLPEDEKRELLDLLYKAYGMPPSASAGRFLSGLLGSCPVLAPVWLSVGLLARCPSILGQRHECVMTPWFLGPGWEQRLIRSVCFL*
## 27                                                                                                                                                                                                                                                                                                                                                                                                                                                        MDKPLTLPDFLAKFDYYMPAIALWTYSRQSGWDTATTPWKTRPFITGCGRKTCTSRLKNDQANYSLNTDDPLIFKSTLDTDYQMTKRDMGFTEEEFKRLNINAAKSSFLPEDEKRELLDLLYKAYGMPPSASAGQNL*
## 28                                                                                                                                                                                                                                                                                                                                                  MAQTPAFDKPKVELHVHLDGSIKPETILYYGRRRGIALPANTAEGLLNVIGMDKPLTLPDFLAKFDYYMPAIAGCREAIKRIAYEFVEMKAKEGVVYVEVRYSPHLLANSKVEPIPWNQAEGDLTPDEVVALVGQGLQEGERDFGVKARSILCCMRHQPNWSPKVVELCKKYQQQTVVAIDLAGDETIPGSSLLPGHVQAYQAVDILKTERLGHGYHTLEDQALYNRLRQENMHFEAQK*
## 29                                                                                                                                                                                                                       MAQTPAFDKPKVELHVHLDGSIKPETILYYGRRGIALPANTAEGLLNVIGMDKPLTLPDFLAKFDYYMPAIAGCREAIKRIAYEFVEMKAKEGVVYVEVRYSPHLLANSKVEPIPWNQAEGDLTPDEVVALVGQGLQEGERDFGVKARSILCCMRHQPNWSPKVVELCKKYQQQTVVAIDLAGDETIPGSSLLPGHVQAYQEAVKSGIHRTVHAGEVGSAEVVKEAVDILKTERLGHGYHTLEDQALYNRLRQENMHFEICPWSSYLTGAWKPDTEHAVIRLKNDQANYSLNTDDPLIFKSTLDTDYQMTKRDMGFTEEEFKRLNINAAKSSFLPEDEKRELLDLLYKAYGMPPSASAGQNL*
## 30                                                                                                                                                                                               MAQTPAFDKPKVELHVHLDGSIKPETILYYGRRRGIALPANTAEGLLNVIGMDKPLTLPDFLAKFDYYMPAIAGCREAIKRIAYEFVEMKAKEGVVYVEVRYSPHLLANSKVEPIPWNQAEGDLTPDEVVALVGQGLQEGERDFGVKARSILCCMRHQPNWSPKVVELCKKYQQQTVVAIDLAGDETIPGSSLLPGHVQAYQHGQGRACRGPPARAPMTWLSPFQEAVKSGIHRTVHAGEVGSAEVVKEAVDILKTERLGHGYHTLEDQALYNRLRQENMHFEICPWSSYLTGAWKPDTEHAVIRLKNDQANYSLNTDDPLIFKSTLDTDYQMTKRDMGFTEEEFKRLNINAAKSSFLPEDEKRELLDLLYKAYGMPPSASAGQNL*
## 31                                                                                                                                                                                  MAQTPAFDKPKVELHVHLDGSIKPETILYYGRRRGIALPANTAEGLLNVIGMDKPLTLPDFLAKFDYYMPAIAGCREAIKRIAYEFVEMKAKEGVVYVEVRYSPHLLANSKVEPIPWNQAEGDLTPDEVVALVGQGLQEGERDFGVKARSILCCMRHQPNWSPKVVELCKKYQQQTVVAIDLAGDETIPGSSLLPGHVQAYQAVKSGIHRTVHAGEVGSAEVVKEAVDILKTERLGHGYHTLEDQALYNRLRQENMHFEICPWSSYLTGAWKPDTEHAVIRLKNDQANYSLNTDDPLIFKSTLDTDYQMTKRDMGFTEEEFKRLNINAAKSSFLPEDEKRELLDLLYKAYGMPPSASAEPLKTPLLQAFTLWSHPNSVGLSNIFTFIPSKKTMISIVSY*
## 32                                                                                                                                                                                                                                                                                                                                                                                                                                                                                                                                                 MAQTPAFDKPKVELHVHLDGSIKPETILYYGSQLQARSGTVRTWRPQH*
## 33                                                                                                                                                                                                                      MAQTPAFDKPKVELHVHLDGSIKPETILYYGRRRGIALPANTAEGLLNVIGMDKPLTLPDFLAKFDYYMPAIAGCREAIKRIAYEFVEMKAKEGVVYVEVRYSPHLLANSKVEPIPWNQAEGDLTPDEVVALVGQGLQEGERDFGVKARSILCCMRHQPNWSPKVVELCKKYQQQTVVAIDLAGDETIPGSSLLPGHVQAYQEAVKSGIHRTVHAGEVGSAEVVKEAVDILKTERLGHGYHTLEDQALYNRLRQENMHFEICPWSSYLTGAWKPDTEHAVIRLKNDQANYSLNTDDPLIFKSTLDTDYQMTKRDMGFTEEEFKRLNINAAKSSFLPEDEKRELLDLLYKAYGMPPSASAGQNL*
## 34                                                                                                                                                                                                                                                                                                                                                                                                                                                               MAQTPAFDKPKVELHVHLDGSIKPETILYYGRRRGIALPANTAEGLLNVIGMDKPLTLPDFLAKFDYYMPAIAGCREAIKRIAYEFVEMKAKEGVVYVEVRYSPHLLANSKVEPIPWNQAELVPQGGGAV*
## 35                                                                                                                                                                                                                                                                                                                                                                                MAQTPAFDKPKVELHVHLDGSIKPETILYYGRRRGIALPANTAEGLLNVIGMDKPLTLPDFLAKFDYYMPAIAVDILKTERLGHGYHTLEDQALYNRLRQENMHFEICPWSSYLTGAWKPDTEHAVIRLKNDQANYSLNTDDPLIFKSTLDTDYQMTKRDMGFTEEEFKRLNINAAKSSFLPEDEKRELLDLLYKAYGMPPSASAGQNL*
## 36                                                                                                                                                                                                 MSSPPPPTPDELLQVLQATAGPPRKFLNYTIMVELHVHLDGSIKPETILYYGRRRGIALPANTAEGLLNVIGMDKPLTLPDFLAKFDYYMPAIAGCREAIKRIAYEFVEMKAKEGVVYVEVRYSPHLLANSKVEPIPWNQAEGDLTPDEVVALVGQGLQEGERDFGVKARSILCCMRHQPNWSPKVVELCKKYQQQTVVAIDLAGDETIPGSSLLPGHVQAYQEAVKSGIHRTVHAGEVGSAEVVKEAVDILKTERLGHGYHTLEDQALYNRLRQENMHFEICPWSSYLTGAWKPDTEHAVIRLKNDQANYSLNTDDPLIFKSTLDTDYQMTKRDMGFTEEEFKRLNINAAKSSFLPEDEKRELLDLLYKAYGMPPSASAGQNL*
## 37                                                                                                                                                                                             MAQTPAFDKPKTEFRSCCPGWSAMARPRLTATFASQVELHVHLDGSIKPETILYYGRRRGIALPANTAEGLLNVIGMDKPLTLPDFLAKFDYYMPAIAGCREAIKRIAYEFVEMKAKEGVVYVEVRYSPHLLANSKVEPIPWNQAEGDLTPDEVVALVGQGLQEGERDFGVKARSILCCMRHQPNWSPKVVELCKKYQQQTVVAIDLAGDETIPGSSLLPGHVQAYQEAVKSGIHRTVHAGEVGSAEVVKEAVDILKTERLGHGYHTLEDQALYNRLRQENMHFEICPWSSYLTGAWKPDTEHAVIRLKNDQANYSLNTDDPLIFKSTLDTDYQMTKRDMGFTEEEFKRLNINAAKSSFLPEDEKRELLDLLYKAYGMPPSASAGQNL*
## 38                                                                                                                                                                                                                                                                                                                                                                                     MAQTPAFDKPKVELHVHLDGSIKPETILYYGRRRGIALPANTAEGLLNVIGMDKPLTLPDFLAKFDYYMPAIAGCREAIKRIAYEFVEMKAKEGVVYVEVRYSPHLLANSKVEPIPWNQAELWTYSRQSGWDTATTPWKTRPFITGCGRKTCTSRSAPGPATSLVPGSRTRSMQSFGSKMTRLTTRSTQMTRSSSSPPWTLITR*
## 39                                                                                                                                                                                                                                                                                                                                                                                                                                                                                                                                            MAQTPAFDKPKTEFRSCCPGWSAMARPRLTATFASQVQVILLPQPPKWNCMST*
## 40                                                                                                                                                                                                                                                                                                                                                                                                               MAQTPAFDKPKVELHVHLDGSIKPETILYYGRRRGIALPANTAEGLLNVIGMDKPLTLPDFLAKFDYYMPAIAGCREAIKRIAYEFVEMKAKEGVVYVEVRYSPHLLANSKVEPIPWNQAEGDLTPDEVVALVGQGLQEGERDFGVKARSILCCMRHQPNDVLNGPPFPHLRMGISLP*
## 41                                                                                                                                                                                             MSTPDRHLVLCPAPTECSVNGSSFVRQRYGGERQGAQVELHVHLDGSIKPETILYYGRRRGIALPANTAEGLLNVIGMDKPLTLPDFLAKFDYYMPAIAGCREAIKRIAYEFVEMKAKEGVVYVEVRYSPHLLANSKVEPIPWNQAEGDLTPDEVVALVGQGLQEGERDFGVKARSILCCMRHQPNWSPKVVELCKKYQQQTVVAIDLAGDETIPGSSLLPGHVQAYQAVKSGIHRTVHAGEVGSAEVVKEAVDILKTERLGHGYHTLEDQALYNRLRQENMHFEICPWSSYLTGAWKPDTEHAVIRLKNDQANYSLNTDDPLIFKSTLDTDYQMTKRDMGFTEEEFKRLNINAAKSSFLPEDEKRELLDLLYKAYGMPPSASAGQNL*
## 42                                                                                                                                                                                                                        MAQTPAFDKPKVELHVHLDGSIKPETILYYGRRGIALPANTAEGLLNVIGMDKPLTLPDFLAKFDYYMPAIAGCREAIKRIAYEFVEMKAKEGVVYVEVRYSPHLLANSKVEPIPWNQAEGDLTPDEVVALVGQGLQEGERDFGVKARSILCCMRHQPNWSPKVVELCKKYQQQTVVAIDLAGDETIPGSSLLPGHVQAYQAVKSGIHRTVHAGEVGSAEVVKEAVDILKTERLGHGYHTLEDQALYNRLRQENMHFEICPWSSYLTGAWKPDTEHAVIRLKNDQANYSLNTDDPLIFKSTLDTDYQMTKRDMGFTEEEFKRLNINAAKSSFLPEDEKRELLDLLYKAYGMPPSASAGQNL*
## 43                                                                                                                                        MAQTPAFDKPKVELHVHLDGSIKPETILYYGRRRGIALPANTAEGLLNVIGMDKPLTLPDFLAKFDYYMPAIAGCREAIKRIAYEFVEMKAKEGVVYVEVRYSPHLLANSKVEPIPWNQAEGDLTPDEVVALVGQGLQEGERDFGVKARSILCCMRHQPNWSPKVVELCKKYQQQTVVAIDLAGDETIPGSSLLPGHVQAYQHGQGRACRGPPARAPMTWLSPFQEAVKSGIHRTVHAGEVGSAEVVKEAVDILKTERLGHGYHTLEDQALYNRLRQENMHFEICPWSSYLTGAWKPDTEHAVIRLKNDQANYSLNTDDPLIFKSTLDTDYQMTKRDMGFTEEEFKRLNINAAKSSFLPEDEKRELLDLLYKAYGMPPSASAGRFLSGLLGSCPVLAPVWLSVGLLARCPSILGQRHECVMTPWFLGPGWEQRLIRSVCFL*
## 44                                                                                                                                                                                                                                                                                                                                                                                                                                                                                                              MAQTPAFDKPKVELHVHLDGSIKPETILYYGRRRGIALPANTAEGLLNVIGMDKPLTLPDFLAKFDYYMPAIGLPGGYQKDRL*
## 45                                                                                                                                                                                                                       MAQTPAFDKPKVELHVHLDGSIKPETILYYGRRRGIALPANTAEGLLNVIGMDKPLTLPDFLAKFDYYMPAIAGCREAIKRIAYEFVEMKAKEGVVYVEVRYSPHLLANSKVEPIPWNQAEGDLTPDEVVALVGQGLQEGERDFGVKARSILCCMRHQPNWSPKVVELCKKYQQQTVVAIDLAGDETIPGSSLLPGHVQAYQAVKSGIHRTVHAGEVGSAEVVKEAVDILKTERLGHGYHTLEDQALYNRLRQENMHFEICPWSSYLTGAWKPDTEHAVIRLKNDQANYSLNTDDPLIFKSTLDTDYQMTKRDMGFTEEEFKRLNINAAKSSFLPEDEKRELLDLLYKAYGMPPSASAGQNL*
## 46                                                                                                                                                                                              MAQTPAFDKPKVELHVHLDGSIKPETILYYGRRRGIALPANTAEGLLNVIGMDKPLTLPDFLAKFDYYMPAIAGCREAIKRIAYEFVEMKAKEGVVYVEVRYSPHLLANSKVEPIPWNQAEGDLTPDEVVALVGQGLQEGERDFGVKARSILCCMRHQPNWSPKVVELCKKYQQQTVVAIDLAGDETIPGSSLLPGHVQAYQEAVKSGIHRTVHAGEVGSAEVVKEAVDILKTERLGHGYHTLEDQALYNRLRQENMHFEICPWSSYLTGAWKPDTEHAVIRLKNDQANYSLNTDDPLIFKSTLDTDYQMTKRDMGFTEEEFKRLNINAAKSSFLPEDEKRELLDLLYKAYGMPPSASAGRSSFLVLVFYDFYNILGTTSCLIFLLL*
## 47                                                                                                                                                                                 MAQTPAFDKPKVELHVHLDGSIKPETILYYGRRRGIALPANTAEGLLNVIGMDKPLTLPDFLAKFDYYMPAIAGCREAIKRIAYEFVEMKAKEGVVYVEVRYSPHLLANSKVEPIPWNQAEGDLTPDEVVALVGQGLQEGERDFGVKARSILCCMRHQPNWSPKVVELCKKYQQQTVVAIDLAGDETIPGSSLLPGHVQAYQEAVKSGIHRTVHAGEVGSAEVVKEAVDILKTERLGHGYHTLEDQALYNRLRQENMHFEICPWSSYLTGAWKPDTEHAVIRLKNDQANYSLNTDDPLIFKSTLDTDYQMTKRDMGFTEEEFKRLNINAAKSSFLPEDEKRELLDLLYKAYGMPPSASAEPLKTPLLQAFTLWSHPNSVGLSNIFTFIPSKKTMISIVSY*
## 48                                                                                                                                                                                               MAQTPAFDKPKVELHVHLDGSIKPETILYYGRRRGIALPANTAEGLLNVIGMDKPLTLPDFLAKFDYYMPAIAGCREAIKRIAYEFVEMKAKEGVVYVEVRYSPHLLANSKVEPIPWNQAEGDLTPDEVVALVGQGLQEGERDFGVKARSILCCMRHQPNWSPKVVELCKKYQQQTVVAIDLAGDETIPGSSLLPGHVQAYQAVKSGIHRTVHAGEVGSAEVVKEAVDILKTERLGHGYHTLEDQALYNRLRQENMHFEICPWSSYLTGAWKPDTEHAVIRLKNDQANYSLNTDDPLIFKSTLDTDYQMTKRDMGFTEEEFKRLNINAAKSSFLPEDEKRELLDLLYKAYGMPPSASAGRSSFLVLVFYDFYNILGTTSCLIFLLL*
## 49 LERGGEAAAAAAAAAAAPGRGSESPVTISRAGNAGELVSPLLLPPTRRRRRRHIQGPGPVLNLPSAAAAPPVARAPEAAGGGSRSEDYSSSPHSAAAAARPLAAEEKQAQSLQPSSSRRSSHYPAAVQSQAAAERGASATAKSRAISILQKKPRHQQLLPSLSSFFFSHRLPDMTAIIKEIVSRNKRRYQEDGFDLDLTYIYPNIIAMGFPAERLEGVYRNNIDDVVRFLDSKHKNHYKIYNLCAERHYDTAKFNCRVAQYPFEDHNPPQLELIKPFCEDLDQWLSEDDNHVAAIHCKAGKGRTGVMICAYLLHRGKFLKAQEALDFYGEVRTRDKKGVTIPSQRRYVYYYSYLLKNHLDYRPVALLFHKMMFETIPMFSGGTCNPQFVVCQLKVKIYSSNSGPTRREDKFMYFEFPQPLPVCGDIKVEFFHKQNKMLKKDKMFHFWVNTFFIPGPEETSEKVENGSLCDQEIDSICSIERADNDKEYLVLTLTKNDLDKANKDKANRYFSPNFKVKLYFTKTVEEPSNPEASSSTSVTPDVSDNEPDHYRYSDTTDSDPENEPFDEDQHTQITKV*
##    entrezgene_id
## 1           5728
## 2           5728
## 3           5728
## 4           5728
## 5           5728
## 6           5728
## 7           5728
## 8           5728
## 9           5728
## 10          5728
## 11          5728
## 12           100
## 13           100
## 14           100
## 15           100
## 16           100
## 17           100
## 18           100
## 19           100
## 20           100
## 21           100
## 22           100
## 23           100
## 24           100
## 25           100
## 26           100
## 27           100
## 28           100
## 29           100
## 30           100
## 31           100
## 32           100
## 33           100
## 34           100
## 35           100
## 36           100
## 37           100
## 38           100
## 39           100
## 40           100
## 41           100
## 42           100
## 43           100
## 44           100
## 45           100
## 46           100
## 47           100
## 48           100
## 49          5728
```

## 6.10 Retrieve known SNPs located on the human chromosome 8 between positions 148350 and 148420

For this example we’ll first have to connect to a different BioMart database, namely snp.

```
snpmart = useEnsembl(biomart = "snp", dataset="hsapiens_snp")
```

The `listAttributes()` and `listFilters()` functions give us an overview of the available attributes and filters.  
From these we need: *refsnp\_id*, *allele*, *chrom\_start* and *chrom\_strand* as attributes; and as filters we’ll use: *chrom\_start*, *chrom\_end* and *chr\_name*.  
666 Note that when a chromosome name, a start position and an end position are jointly used as filters,
the BioMart webservice interprets this as return everything from the given chromosome between the given start and end positions. Putting our selected attributes and filters into `getBM()` gives:

```
getBM(attributes = c('refsnp_id','allele','chrom_start','chrom_strand'), 
      filters = c('chr_name','start','end'), 
      values = list(8, 148350, 148420), 
      mart = snpmart)
```

```
##       refsnp_id allele chrom_start chrom_strand
## 1  rs1450830176    G/C      148350            1
## 2  rs1360310185    C/T      148352            1
## 3  rs1434776028    A/T      148353            1
## 4  rs1413161474    C/T      148356            1
## 5  rs1410590268    A/G      148365            1
## 6  rs1193735780  T/A/C      148368            1
## 7  rs1409139861    C/T      148371            1
## 8   rs868546642    A/G      148372            1
## 9   rs547420070    A/C      148373            1
## 10 rs1236874674    C/T      148375            1
## 11 rs1207902742    C/T      148376            1
## 12 rs1437239557    T/C      148377            1
## 13 rs1160135941    T/G      148379            1
## 14 rs1229249227    A/T      148380            1
## 15 rs1584865972    C/G      148381            1
## 16 rs1328678285    C/G      148390            1
## 17   rs77274555  G/A/C      148391            1
## 18  rs567299969  T/A/C      148394            1
## 19 rs1457776094  A/C/G      148395            1
## 20 rs1292078334    C/T      148404            1
## 21 rs1456392146    A/T      148405            1
## 22  rs368076569    G/A      148407            1
## 23 rs1166604298    A/G      148408            1
## 24 rs1393545673  A/G/T      148410            1
## 25 rs1180076939    A/T      148413            1
## 26 rs1476313471    A/G      148414            1
## 27 rs1248424402    T/C      148417            1
## 28 rs1207939741    A/T      148419            1
```

## 6.11 Given the human gene TP53, retrieve the human chromosomal location of this gene and also retrieve the chromosomal location and RefSeq id of its homolog in mouse.

The `getLDS()` (Get Linked Dataset) function provides functionality to link 2 BioMart datasets which each other and construct a query over the two datasets. In Ensembl, linking two datasets translates to retrieving homology data across species.

The usage of getLDS is very similar to `getBM()`. The linked dataset is provided by a separate `Mart` object and one has to specify filters and attributes for the linked dataset. Filters can either be applied to both datasets or to one of the datasets. Use the listFilters and listAttributes functions on both `Mart` objects to find the filters and attributes for each dataset (species in Ensembl). The attributes and filters of the linked dataset can be specified with the attributesL and filtersL arguments. Entering all this information into `getLDS()` gives:

```
human <- useEnsembl("ensembl", dataset = "hsapiens_gene_ensembl")
mouse <- useEnsembl("ensembl", dataset = "mmusculus_gene_ensembl")
getLDS(attributes = c("hgnc_symbol","chromosome_name", "start_position"),
       filters = "hgnc_symbol", values = "TP53",
       mart = human,
       attributesL = c("refseq_mrna","chromosome_name","start_position"), 
       martL = mouse)
```

```
## Error: biomaRt has encountered an unexpected server error.
## Consider trying one of the Ensembl mirrors (for more details look at ?useEnsembl)
```

# 7 Connection troubleshooting

It is not uncommon to encounter connection problems when trying to access online resources such as the Ensembl BioMart.
In this section we list error messages that have been reported by users, along with suggested code to fix the problem. If a suggested solution doesn’t work, or you have a new error not listed here, please reported it on the Bioconductor Support Site.

## 7.1 *biomaRt* specific solutions

If you are using *biomaRt* directly make sure you are using `useEnsembl()` to create the **Mart** object, rather than `useMart()`. `useEnsembl()` is aware of some specific connection details required to connect to Ensembl, and using it may fix any connection problems without requiring you to do anything further.

## 7.2 Global connection settings

If you are unable to modify the *biomaRt* code (for example if you are using another package that calls *biomaRt* as part of one if its functions) it’s still possible to modify the connection settings for your R session. Below are some reported error messages and code that has been known to resolve them. You will need to execute this code only once in an R session, but the settings will not persist between R sessions.

### 7.2.1 Error: “SSL certificate problem”

**Error message**

```
Error in curl::curl_fetch_memory(url, handle = handle) :
SSL certificate problem: unable to get local issuer certificate
```

**Fix**

```
httr::set_config(httr::config(ssl_verifypeer = FALSE))
```

### 7.2.2 Error: “sslv3 alert handshake failure”

**Error message**

```
Error in curl::curl_fetch_memory(url, handle = handle) : 
error:14094410:SSL routines:ssl3_read_bytes:sslv3 alert handshake failure
```

**Fix**

If you’re running Ubuntu 20.04 or newer the following command should fix the issue.

```
httr::set_config(httr::config(ssl_cipher_list = "DEFAULT@SECLEVEL=1"))
```

If you encounter this error on Fedora 33, the code above doesn’t seem to work. At the moment, the only workaround we have discovered is to change the security settings at the system level. Please see more information at fedoraproject.org and trouble shooting discussion at GitHub. This change can be applied by running the following command in a terminal outside of R, but please consider whether this is something you want to change. You could also consider alerting Ensembl to this issue.

```
update-crypto-policies --set LEGACY
```

# 8 Session Info

```
sessionInfo()
```

```
## R version 4.2.3 (2023-03-15)
## Platform: x86_64-pc-linux-gnu (64-bit)
## Running under: Ubuntu 20.04.6 LTS
## 
## Matrix products: default
## BLAS:   /home/biocbuild/bbs-3.16-bioc/R/lib/libRblas.so
## LAPACK: /home/biocbuild/bbs-3.16-bioc/R/lib/libRlapack.so
## 
## locale:
##  [1] LC_CTYPE=en_US.UTF-8       LC_NUMERIC=C               LC_TIME=en_GB             
##  [4] LC_COLLATE=C               LC_MONETARY=en_US.UTF-8    LC_MESSAGES=en_US.UTF-8   
##  [7] LC_PAPER=en_US.UTF-8       LC_NAME=C                  LC_ADDRESS=C              
## [10] LC_TELEPHONE=C             LC_MEASUREMENT=en_US.UTF-8 LC_IDENTIFICATION=C       
## 
## attached base packages:
## [1] stats     graphics  grDevices utils     datasets  methods   base     
## 
## other attached packages:
## [1] biomaRt_2.54.1   BiocStyle_2.26.0
## 
## loaded via a namespace (and not attached):
##  [1] prettyunits_1.1.1      png_0.1-8              Biostrings_2.66.0      digest_0.6.31         
##  [5] utf8_1.2.3             BiocFileCache_2.6.1    R6_2.5.1               GenomeInfoDb_1.34.9   
##  [9] stats4_4.2.3           RSQLite_2.3.0          evaluate_0.20          httr_1.4.5            
## [13] highr_0.10             pillar_1.8.1           zlibbioc_1.44.0        rlang_1.1.0           
## [17] progress_1.2.2         curl_5.0.0             jquerylib_0.1.4        blob_1.2.4            
## [21] S4Vectors_0.36.2       rmarkdown_2.20         stringr_1.5.0          RCurl_1.98-1.10       
## [25] bit_4.0.5              compiler_4.2.3         xfun_0.37              pkgconfig_2.0.3       
## [29] BiocGenerics_0.44.0    htmltools_0.5.4        tidyselect_1.2.0       KEGGREST_1.38.0       
## [33] tibble_3.2.1           GenomeInfoDbData_1.2.9 bookdown_0.33          codetools_0.2-19      
## [37] IRanges_2.32.0         XML_3.99-0.14          fansi_1.0.4            crayon_1.5.2          
## [41] dplyr_1.1.0            dbplyr_2.3.1           withr_2.5.0            bitops_1.0-7          
## [45] rappdirs_0.3.3         jsonlite_1.8.4         lifecycle_1.0.3        DBI_1.1.3             
## [49] magrittr_2.0.3         cli_3.6.0              stringi_1.7.12         cachem_1.0.7          
## [53] XVector_0.38.0         xml2_1.3.3             bslib_0.4.2            ellipsis_0.3.2        
## [57] filelock_1.0.2         vctrs_0.6.0            generics_0.1.3         tools_4.2.3           
## [61] bit64_4.0.5            Biobase_2.58.0         glue_1.6.2             purrr_1.0.1           
## [65] hms_1.1.2              fastmap_1.1.1          yaml_2.3.7             AnnotationDbi_1.60.2  
## [69] BiocManager_1.30.20    memoise_2.0.1          knitr_1.42             sass_0.4.5
```
