# Supplementary material for: Computational reconstruction of evolutionary selection in human brain networks
Source: Front Neuroinform. 2026 Jan 26;19:1623174. doi: 10.3389/fninf.2025.1623174 (PMC12883834; doi:10.3389/fninf.2025.1623174)
Supplement: Supplementary file 1 [file Data_Sheet_1.zip › Piszczek et al Genetic-Trait-Mining/R_package_sources/biomaRt_2.54.1.tar.gz!/biomaRt/inst/doc/accessing_other_marts.html]

Using a BioMart other than Ensembl


# Using a BioMart other than Ensembl

Steffen Durinck, Wolfgang Huber, Mike Smith

#### 20 March 2023

#### Package

biomaRt 2.54.1

# Contents

- 1 Introduction
- 2 Using a BioMart other than Ensembl
  - 2.1 Wormbase
  - 2.2 Phytozome
    - 2.2.1 Version 12
    - 2.2.2 Version 13
- 3 Session Info

# 1 Introduction

In recent years a wealth of biological data has become available in public data repositories. Easy access to these valuable data resources and firm integration with data analysis is needed for comprehensive bioinformatics data analysis. The *biomaRt* package, provides an interface to a growing collection of databases implementing the BioMart software suite. The package enables retrieval of large amounts of data in a uniform way without the need to know the underlying database schemas or write complex SQL queries. Examples of BioMart databases are Ensembl, Uniprot and HapMap. These major databases give *biomaRt* users direct access to a diverse set of data and enable a wide range of powerful online queries from R.

# 2 Using a BioMart other than Ensembl

There are a small number of non-Ensembl databases that offer a BioMart interface to their data. The *biomaRt* package can be used to access these in a very similar fashion to Ensembl. The majority of *biomaRt* functions will work in the same manner, but the construction of the initial Mart object requires slightly more setup. In this section we demonstrate the setting requires to query Wormbase ParaSite and Phytozome. First we need to load *biomaRt*.

```
library(biomaRt)
```

## 2.1 Wormbase

To demonstrate the use of the *biomaRt* package with non-Ensembl databases the next query is performed using the Wormbase ParaSite BioMart. In this example, we use the `listMarts()` function to find the name of the available marts, given the URL of Wormbase. We use this to connect to Wormbase BioMart using the `useMart()` function.111 Note that we use the `https` address and must provide the port as `443`. Queries to WormBase will fail without these options.

```
listMarts(host = "parasite.wormbase.org")
```

```
##         biomart      version
## 1 parasite_mart WBPS 17 Mart
```

```
wormbase <- useMart(biomart = "parasite_mart", 
                    host = "https://parasite.wormbase.org", 
                    port = 443)
```

We can then use functions described earlier in this vignette to find and select the gene dataset, and print the first 6 available attributes and filters. Then we use a list of gene names as filter and retrieve associated transcript IDs and the transcript biotype.

```
listDatasets(wormbase)
```

```
##     dataset          description version
## 1 wbps_gene All Species (WBPS17)      17
```

```
wormbase <- useDataset(mart = wormbase, dataset = "wbps_gene")
head(listFilters(wormbase))
```

```
##                  name     description
## 1     species_id_1010          Genome
## 2 nematode_clade_1010  Nematode Clade
## 3     chromosome_name Chromosome name
## 4               start           Start
## 5                 end             End
## 6              strand          Strand
```

```
head(listAttributes(wormbase))
```

```
##                      name        description         page
## 1          species_id_key      Internal Name feature_page
## 2    production_name_1010     Genome project feature_page
## 3       display_name_1010        Genome name feature_page
## 4        taxonomy_id_1010        Taxonomy ID feature_page
## 5 assembly_accession_1010 Assembly accession feature_page
## 6     nematode_clade_1010     Nematode clade feature_page
```

```
getBM(attributes = c("external_gene_id", "wbps_transcript_id", "transcript_biotype"), 
      filters = "gene_name", 
      values = c("unc-26","his-33"), 
      mart = wormbase)
```

```
##   external_gene_id wbps_transcript_id transcript_biotype
## 1           his-33         F17E9.13.1     protein_coding
## 2           unc-26          JC8.10a.1     protein_coding
## 3           unc-26          JC8.10a.2     protein_coding
## 4           unc-26          JC8.10b.1     protein_coding
## 5           unc-26          JC8.10c.1     protein_coding
## 6           unc-26          JC8.10c.2     protein_coding
## 7           unc-26          JC8.10d.1     protein_coding
```

## 2.2 Phytozome

### 2.2.1 Version 12

The Phytozome 12 BioMart was retired in August 2021 and can not longer be accessed.

### 2.2.2 Version 13

Version 13 of Phyotozome can be found at https://phytozome-next.jgi.doe.gov/ and if you wish to query that version the URL used to create the Mart object must reflect that.

```
phytozome_v13 <- useMart(biomart = "phytozome_mart", 
                dataset = "phytozome", 
                host = "https://phytozome-next.jgi.doe.gov")
```

Once this is set up the usual *biomaRt* functions can be used to interrogate the database options and run queries.

```
getBM(attributes = c("organism_name", "gene_name1"), 
      filters = "gene_name_filter", 
      values = "82092", 
      mart = phytozome_v13)
```

```
##          organism_name gene_name1
## 1 Smoellendorffii_v1.0      82092
```

# 3 Session Info

```
sessionInfo()
```

```
## R version 4.2.3 (2023-03-15)
## Platform: x86_64-pc-linux-gnu (64-bit)
## Running under: Ubuntu 20.04.6 LTS
## 
## Matrix products: default
## BLAS:   /home/biocbuild/bbs-3.16-bioc/R/lib/libRblas.so
## LAPACK: /home/biocbuild/bbs-3.16-bioc/R/lib/libRlapack.so
## 
## locale:
##  [1] LC_CTYPE=en_US.UTF-8       LC_NUMERIC=C               LC_TIME=en_GB             
##  [4] LC_COLLATE=C               LC_MONETARY=en_US.UTF-8    LC_MESSAGES=en_US.UTF-8   
##  [7] LC_PAPER=en_US.UTF-8       LC_NAME=C                  LC_ADDRESS=C              
## [10] LC_TELEPHONE=C             LC_MEASUREMENT=en_US.UTF-8 LC_IDENTIFICATION=C       
## 
## attached base packages:
## [1] stats     graphics  grDevices utils     datasets  methods   base     
## 
## other attached packages:
## [1] biomaRt_2.54.1   BiocStyle_2.26.0
## 
## loaded via a namespace (and not attached):
##  [1] prettyunits_1.1.1      png_0.1-8              Biostrings_2.66.0      digest_0.6.31         
##  [5] utf8_1.2.3             BiocFileCache_2.6.1    R6_2.5.1               GenomeInfoDb_1.34.9   
##  [9] stats4_4.2.3           RSQLite_2.3.0          evaluate_0.20          httr_1.4.5            
## [13] highr_0.10             pillar_1.8.1           zlibbioc_1.44.0        rlang_1.1.0           
## [17] progress_1.2.2         curl_5.0.0             jquerylib_0.1.4        blob_1.2.4            
## [21] S4Vectors_0.36.2       rmarkdown_2.20         stringr_1.5.0          RCurl_1.98-1.10       
## [25] bit_4.0.5              compiler_4.2.3         xfun_0.37              pkgconfig_2.0.3       
## [29] BiocGenerics_0.44.0    htmltools_0.5.4        tidyselect_1.2.0       KEGGREST_1.38.0       
## [33] tibble_3.2.1           GenomeInfoDbData_1.2.9 bookdown_0.33          codetools_0.2-19      
## [37] IRanges_2.32.0         XML_3.99-0.14          fansi_1.0.4            crayon_1.5.2          
## [41] dplyr_1.1.0            dbplyr_2.3.1           withr_2.5.0            bitops_1.0-7          
## [45] rappdirs_0.3.3         jsonlite_1.8.4         lifecycle_1.0.3        DBI_1.1.3             
## [49] magrittr_2.0.3         cli_3.6.0              stringi_1.7.12         cachem_1.0.7          
## [53] XVector_0.38.0         xml2_1.3.3             bslib_0.4.2            ellipsis_0.3.2        
## [57] filelock_1.0.2         vctrs_0.6.0            generics_0.1.3         tools_4.2.3           
## [61] bit64_4.0.5            Biobase_2.58.0         glue_1.6.2             purrr_1.0.1           
## [65] hms_1.1.2              fastmap_1.1.1          yaml_2.3.7             AnnotationDbi_1.60.2  
## [69] BiocManager_1.30.20    memoise_2.0.1          knitr_1.42             sass_0.4.5
```

```
warnings()
```
